# CRISPR and biochemical screens identify MAZ as a cofactor in CTCF-mediated insulation at *Hox* clusters

**DOI:** 10.1038/s41588-021-01008-5

**Published:** 2022-02-10

**Authors:** Havva Ortabozkoyun, Pin-Yao Huang, Hyunwoo Cho, Varun Narendra, Gary LeRoy, Edgar Gonzalez-Buendia, Jane A. Skok, Aristotelis Tsirigos, Esteban O. Mazzoni, Danny Reinberg

**Affiliations:** 1 Howard Hughes Medical Institute, NYU Grossman School of Medicine, New York, NY USA; 2 Department of Biochemistry and Molecular Pharmacology, NYU Grossman School of Medicine, New York, NY USA; 3 Department of Pathology, NYU Grossman School of Medicine, New York, NY USA; 4 Applied Bioinformatics Laboratories, NYU Grossman School of Medicine, New York, NY USA; 5 Department of Radiation Oncology, NYU Grossman School of Medicine, New York, NY USA; 6 Department of Medicine, Memorial Sloan Kettering Cancer Center, New York, NY USA; 7 Institute for Computational Medicine, NYU Grossman School of Medicine, New York, NY USA; 8 Department of Biology, New York University, New York, NY USA

**Keywords:** Epigenetics, Gene regulation, Gene targeting, Stem cells, Pattern formation

## Abstract

CCCTC-binding factor (CTCF) is critical to three-dimensional genome organization. Upon differentiation, CTCF insulates active and repressed genes within *Hox* gene clusters. We conducted a genome-wide CRISPR knockout (KO) screen to identify genes required for CTCF-boundary activity at the *HoxA* cluster, complemented by biochemical approaches. Among the candidates, we identified Myc-associated zinc-finger protein (MAZ) as a cofactor in CTCF insulation. MAZ colocalizes with CTCF at chromatin borders and, similar to CTCF, interacts with the cohesin subunit RAD21. MAZ KO disrupts gene expression and local contacts within topologically associating domains. Similar to CTCF motif deletions, MAZ motif deletions lead to derepression of posterior *Hox* genes immediately after CTCF boundaries upon differentiation, giving rise to homeotic transformations in mouse. Thus, MAZ is a factor contributing to appropriate insulation, gene expression and genomic architecture during development.

## Abstract (web summary)

Genome-wide screens identify several genes, including MAZ, required for CTCF-mediated insulation. MAZ interacts with cohesin, and MAZ motif deletions derepress posterior *Hox* gene expression, leading to homeotic transformations in mouse.

## Main

The precise regulation of gene expression is required to ensure proper embryonic development. Beyond the DNA sequence, the chromatin structure and spatial organization of the genome regulate transcriptional output. The genomes of higher eukaryotes are tightly folded and packaged within the nucleus^1^. The partitioning of the genome into independent chromatin domains occurs via insulators. Although several insulators are present in *Drosophila*^2^, CTCF is the main insulator protein in vertebrates^3–5^. CTCF is a highly conserved, ubiquitously expressed, 11-zinc-finger protein^6^ that is critical for development^7,8^ and enriched at the borders of topologically associating domains (TADs)^9–11^. Among the many proteins associated with CTCF at different loci^4,12^, only the cohesin complex colocalizes to most CTCF binding sites and is required for CTCF function^13,14^. CTCF-boundary activity is context dependent^15^. CTCF functions as a boundary between active and repressed chromatin domains, decorated by Trithorax and Polycomb, respectively, at *Hox* clusters upon differentiation of mouse embryonic stem cells (ESCs) into cervical motor neurons (MNs)^16,17^. This dynamic compartmentalization of *Hox* clusters into antagonistic domains allows CTCF-mediated looping to reshape regulatory interactions. Although there is a cell-type-specific modulation of CTCF-boundary activity, CTCF and cohesin occupancy appears stable across *Hox* clusters during the differentiation of ESCs into cervical MNs^16,18^. Thus, during differentiation, additional regulatory factors appear to be necessary to foster CTCF-mediated insulation properties.

**Fig. 1:**
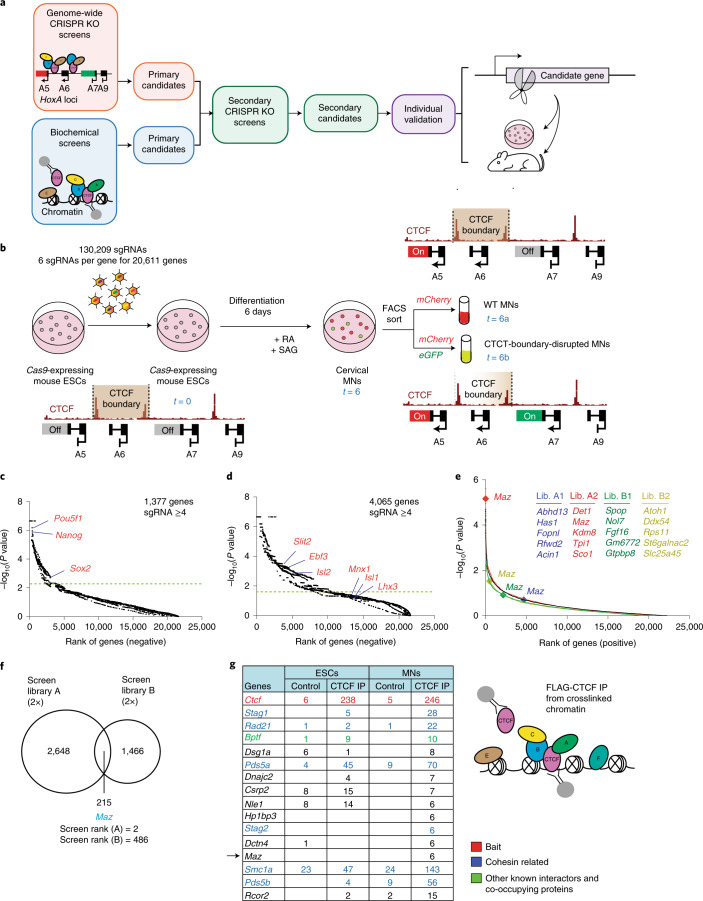
Genome-wide CRISPR loss-of-function screen to identify factors that affect the insulator function of CTCF, complemented with biochemical approaches. **a**, Layout of genetic and biochemical approaches for identification of candidates influencing the insulation function of CTCF. **b**, Layout of the genetic loss-of-function screen that separates MNs with a CTCF-boundary disruption from those with an intact boundary. RA, all-*trans*-retinoic acid; SAG, smoothened agonist. **c**, Rank of genes underrepresented in ESCs compared to the plasmid library. Cutoff line indicates FDR < 0.05. **d**, Rank of genes underrepresented in MNs compared to ESCs. Cutoff line indicates FDR < 0.05. **e**, Rank of genes overrepresented in double-positive MNs compared to *mCherry*-positive MNs in four genome-wide screens. Top candidates are listed for each screen (all candidates are listed in Supplementary Dataset 2). One of the top candidates is indicated on the plot in each independent screen. Lib., library. **f**, Venn diagram showing the overlap of CTCF-boundary-related candidates identified in four independent screens (two for library A and two for library B). *P* value cutoff = 0.05. **g**, Crosslinked FLAG-CTCF ChIP-MS in ESCs and MNs results in identification of known CTCF interactors and novel proteins. The peptide counts in FLAG-CTCF immunoprecipitations were normalized to control FLAG immunoprecipitations in untagged cells. The list is ranked based on CTCF immunoprecipitation/control ratios in MNs. IP, immunoprecipitation.

To identify such putative factors affecting CTCF-boundary activity, we devised an unbiased genome-wide loss-of-function genetic screen involving a functional CTCF boundary within the *HoxA* cluster in cervical MNs. We complemented this screen with biochemical approaches to identify CTCF partners and colocalizing proteins on chromatin in ESCs and MNs (Fig. 1a.). We identified MAZ as a CTCF cofactor functioning to insulate active chromatin boundaries from spreading into repressive regions at *Hox* clusters, among other candidates that were narrowed down via secondary loss-of-function screens. Through a series of functional assays performed in vitro and in vivo during development, we demonstrate that MAZ is integral to appropriate gene expression and architectural genome organization in the context of CTCF and cohesin.

## Results

### A dual reporter of *Hox* gene expression at the *HoxA* cluster

**Extended Data Fig. 1:**
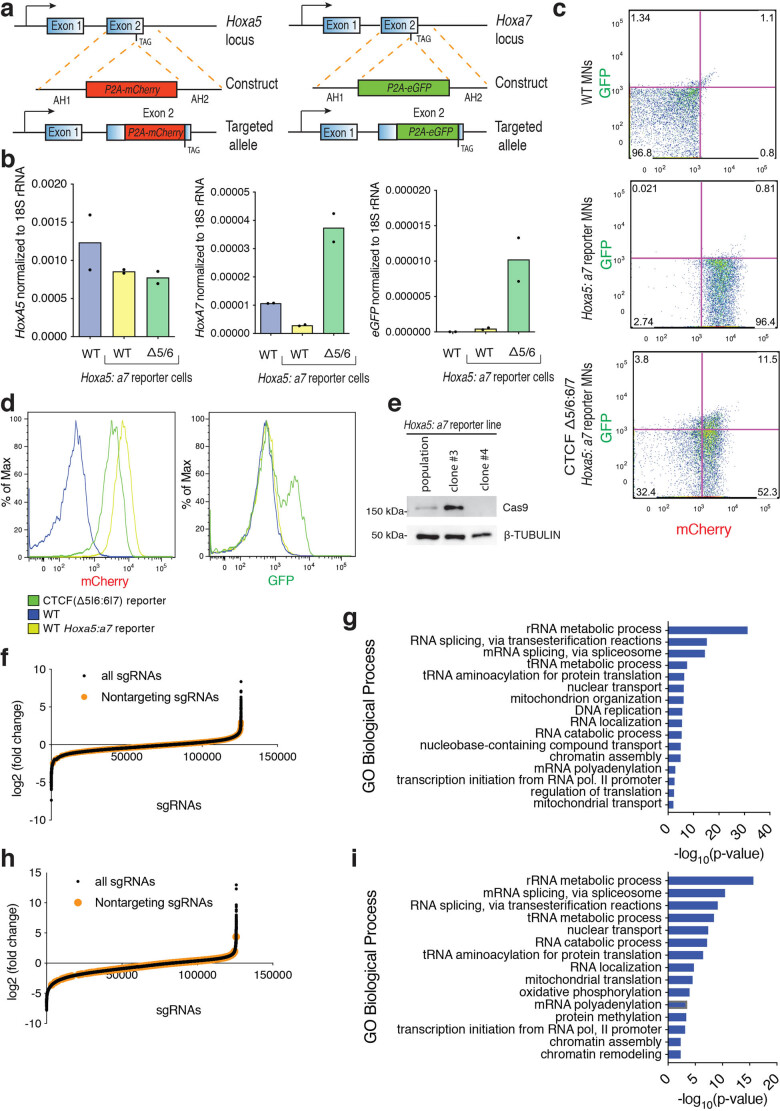
Genome-wide CRISPR KO screen shows loss of essential genes. **a**, Strategy for generating the *Hoxa5:a7* reporter mESC line via CRISPR. AH1, AH2: arm of homology 1, 2, respectively. **b**, RT-qPCR signal for *Hoxa5, Hoxa7*, and *eGFP* normalized to 18S ribosomal RNA in WT MNs, *Hoxa5:a7* reporter MNs, and CTCF(Δ5|6) *Hoxa5:a7* reporter MNs. MNs were obtained through *in vitro* differentiation of ESCs in two biological replicates. Δ5|6 denotes a CTCF binding site deletion between *Hoxa5*-*6*. **c**, FACS data showing mCherry and GFP reporter expression in WT MNs (top), *Hoxa5:a7* reporter MNs (middle), and CTCF(Δ5|6:6|7) *Hoxa5:a7* reporter MNs (bottom). These plots are one representative of three biological replicates. Percentage of positive population in each quadrant is indicated (see Supplementary Fig. 7a for gating of cells). **d**, Flow cytometry analysis of mCherry and GFP reporter expression in MNs with the indicated genotypes: WT, *Hoxa5:a7* reporter, and CTCF(Δ5|6:6|7) reporter (see Extended Data Fig. 4 for RT-qPCR of *Hox* genes and Supplementary Fig. 7a for gating of cells). These plots are one representative of three biological replicates. Δ5|6:6|7 denotes 2 CTCF binding site deletions. **e**, Cas9 protein expression in dual-reporter mESCs transduced with *Cas9* lentivirus and selected with blasticidin. β-TUBULIN serves as loading control. **f**, Fold change of sgRNAs in the primary screens comparing ESCs to plasmid library. **g**, Gene Ontology (GO) analysis of biological processes in negatively selected genes in ESCs compared to plasmid library (FDR < 0.05). PANTHER overrepresentation test tool was used for GO analysis. **h**, Fold change of sgRNAs in the primary screens comparing MN population to ESC population. **i**, GO analysis of biological processes in negatively selected genes in MNs compared to ESCs (FDR < 0.05). PANTHER overrepresentation test tool was used for GO analysis. Source data

We aimed to identify boundary-associated factors that function to insulate the anterior from the posterior region of the *HoxA* cluster. To this end, we focused on the CTCF boundary that forms upon ESC differentiation into cervical MNs^16,17^. This CTCF boundary insulates active and repressed chromatin domains at the *HoxA* cluster, and its loss gives rise to defined transcriptional repercussions in cervical MNs^16,17^. We constructed a dual-reporter ESC line (*Hoxa5:a7* ESCs) containing distinct fluorescent reporters of endogenous *Hox* gene expression on each side of the CTCF-demarcated boundary in the *HoxA* cluster using CRISPR technology^19^ (Fig. 1b and Extended Data Fig. 1a). The relative expression of *Hox* genes can be assayed in single cells, and any activity mediating CTCF-boundary formation can be assessed in this *Hoxa5:a7* dual-reporter system. As expected based on previous studies^16,20,21^, *Hoxa5-P2A-mCherry* reporter expression was induced during cervical MN differentiation, whereas *Hoxa7-P2A-eGFP* remained repressed (Extended Data Fig. 1b–d). To confirm that *Hoxa7-P2A-eGFP* could report defects in the formation of the CTCF-dependent boundary, we deleted the CTCF binding sites between *Hoxa5* and *Hoxa7* genes in ESCs (CTCF (Δ5|6) or CTCF (Δ5|6:6|7), respectively) and demonstrated the derepression of *Hoxa7-P2A-eGFP* by fluorescence-activated cell sorting (FACS) analysis and reverse transcription quantitative polymerase chain reaction (RT-qPCR) (Extended Data Fig. 1b–d), as previously reported^16^ (Supplementary Note 1). The ~10–15% *Hoxa7-P2A-eGFP*-positive cells (Extended Data Fig. 1c,d) allowed for enough of a dynamic range to identify mutants that decreased or increased CTCF insulating properties.

### Genome-wide CRISPR loss-of-function screen for CTCF-boundary function

To identify factors required for the integrity of the CTCF boundary, we performed an unbiased loss-of-function genetic screen on the *Hoxa5:a7* dual-reporter ESCs using a pooled genome-wide library of single-guide RNAs (sgRNAs)^22^, as shown schematically in Fig. 1b. A *Hoxa5:a7* ESC clone expressing *Cas9* (Extended Data Fig. 1e) was transduced with the pooled lentiviral sgRNAs at a low multiplicity of infection (~0.4), as applied previously^22,23^, such that each transduced cell expressed a single sgRNA. The reporter ESCs (*t* = 0) were then differentiated into cervical MNs (*t* = 6) via the addition of all-*trans*-retinoic acid/smoothened agonist^24^ and sorted by FACS into two MN populations: (1) wild-type (WT) MNs (*mCherry-*positive/*eGFP-*negative cells; *t* = 6a) and (2) CTCF-boundary-disrupted MNs (double-positive cells; t = 6b). By preparing libraries at each time point, the relative sgRNA representation at *t* = 0, 6, 6a and 6b were compared using next-generation sequencing, as described previously^22,23,25,26^. This screen setup enabled identification of three sets of genes: (1) essential genes in ESCs (negative selection), (2) essential/differentiation-related genes in MNs (negative selection) and (3) genes affecting CTCF-boundary function (positive selection) (Supplementary Note 2).

### Identification of factors affecting CTCF insulation function

As expected from a functional screen, we observed selective loss of essential genes in the starting population (ESCs, *t* = 0) compared to plasmid library (Fig. 1c, Extended Data Fig. 1f,g and Supplementary Dataset 1a), and further loss of genes essential/required for MN differentiation (MN, *t* = 6) compared to the ESC population (*t* = 0) (Fig. 1d, Extended Data Fig. 1h,i and Supplementary Dataset 1b), indicating the success of the screen. Among genes underrepresented in MNs compared to ESCs (false discovery rate (FDR) < 0.05), we observed Polycomb group genes, CTCF, cohesin components and several components related to the MN differentiation pathway. Our genome-wide screens were performed in duplicates by using independent genome-wide sublibraries (library A and library B) containing three sgRNAs per gene, resulting in four independent screens. In each screen, we identified ~1,000 genes positively selected in double-positive cells (CTCF-boundary-disrupted MNs, *t* = 6b) compared to *mCherry*-positive cells (WT MNs, *t* = 6a) using MAGeCK tools^27,28^ (Fig. 1e and Supplementary Dataset 2). Based on the four independent sublibrary screens, we narrowed down the list of candidates in CTCF-boundary-disrupted MNs compared to WT MNs to 215 genes (Fig. 1e,f and Supplementary Dataset 3). Notably, *Maz* was identified as a top candidate (rank = 2) in one of the genome-wide screens with library A and also detected in a similar screen (rank = 486) with library B containing an independent set of sgRNAs (Fig. 1e,f and Supplementary Datasets 2 and 3).

### Identification of proteins colocalizing with CTCF on chromatin

**Extended Data Fig. 2:**
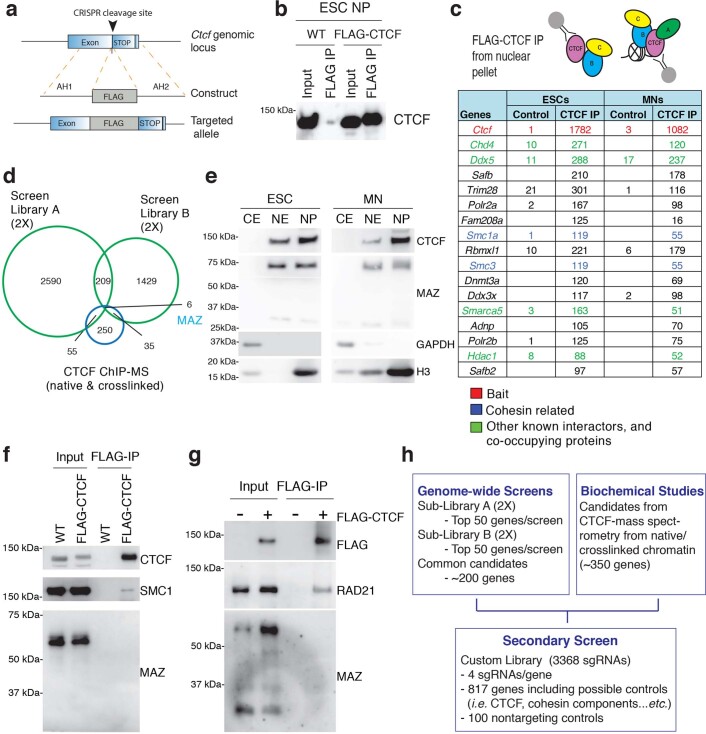
Native ChIP-MS to identify CTCF colocalizing proteins. **a**, FLAG-tag integrated at the C-terminus of CTCF via CRISPR genome editing. AH1, 2: arm of homology 1, 2, respectively. **b**, FLAG pull-down followed by CTCF western blot in benzonase solubilized nuclear pellet (NP) of ESCs. IP, immunoprecipitation. **c**, Native FLAG-CTCF immunoprecipitation in ESCs and MNs results in identification of known CTCF interactors and novel proteins. The mean peptide counts from two biological replicates of FLAG-CTCF immunoprecipitations were normalized to the control FLAG immunoprecipitation from untagged cells. Candidates filtered from the top of the list are shown (see Supplementary Dataset 4 for all). IP, immunoprecipitation. **d**, Venn diagram showing the overlap of CTCF-boundary related candidates identified in 4 independent (two for library A and two for library B) screens and CTCF ChIP-MS (native and crosslinked) approaches (see Supplementary Dataset 5 for overlapping candidates). *P* value cutoff = 0.05 for screens. See Supplementary Dataset 2 and Supplementary Dataset 4 for statistics in each screen and mass-spectrometry experiments. **e**, Western blot analysis of CTCF and MAZ in different cellular fractions in mESCs and MNs. CE: cytoplasmic extract, NE: nuclear extract, NP: nuclear pellet. **f**, Western blot analysis of CTCF, SMC1, and MAZ upon FLAG-CTCF immunoprecipitation from nuclear pellet of mESCs. IP, immunoprecipitation. **g**, Western blot analysis of FLAG, RAD21, and MAZ upon FLAG-CTCF immunoprecipitation from 293FT nuclear extract. IP, immunoprecipitation. **h**, Schematic of candidate selection from genetic and biochemical approaches for secondary loss-of-function screens. Source data

We complemented the locus-specific genetic screen with orthogonal biochemical approaches for the identification of proteins colocalizing with CTCF on chromatin. Unlike previous studies that aimed to identify CTCF partner proteins in soluble cellular fractions through the use of overexpression-based systems^12,29^, we identified proteins colocalizing with endogenous CTCF on chromatin that may or may not interact with CTCF but nonetheless may be important for its insulation properties in situ. To pull down CTCF under endogenous conditions, we generated an ESC line containing C-terminal FLAG-tagged CTCF via CRISPR technology^19^ (Extended Data Fig. 2a) and confirmed successful FLAG-CTCF immunoprecipitation from the nuclear fraction of ESCs (Extended Data Fig. 2b–f and Extended Data Fig. 2g for the immunoprecipitation in 293FT cells). To expand and identify factors colocalizing with CTCF on chromatin, we applied two biochemical methods: (1) FLAG-CTCF immunoprecipitation from native chromatin in ESCs and MNs (Extended Data Fig. 2c) and (2) FLAG-CTCF immunoprecipitation from crosslinked chromatin in ESCs and MNs (Fig. 1g), an adapted version of the chromatin immunoprecipitation (ChIP) mass spectrometry (MS) approach described previously^30–33^ (Supplementary Note 3). In both FLAG-CTCF ChIP-MS approaches, we identified known interactors and novel proteins interacting or cobinding with CTCF (Fig. 1g and Extended Data Fig. 2c; all candidates are listed in Supplementary Dataset 4). As expected, we recovered CTCF, cohesin components and accessory subunits and other chromatin remodelers (Fig. 1g and Extended Data Fig. 2c). Although the overlap between genetic and biochemical approaches is limited (Extended Data Fig. 2d and Supplementary Dataset 5; Supplementary Dataset 2 versus Supplementary Dataset 4 and Fig. 1g), the candidates identified in both approaches have the potential to be critical for CTCF function at the *HoxA* cluster and genome-wide, respectively. Interestingly, MAZ was identified uniquely in the crosslinked-based CTCF ChIP-MS, thereby constituting a representative candidate that overlaps with those identified from the *Hox*-related functional screens. Thus, MAZ might serve a role in regulating the CTCF boundary at the *Hox* loci. MAZ was also reported to colocalize with CTCF at ~48% of binding sites based on ENCODE ChIP sequencing (ChIP-seq) data in K562 cells^34^, as recently confirmed^35^_._ In a systematic study investigating DNA binding proteins at chromatin loops, the combinations of MAX-MYC-MAZ-CHD2 and CTCF-RAD21-SMC3 were reported^36^. Moreover, an algorithm detecting combinatorial motifs for transcription factors has revealed the presence of MAZ and CTCF along with others within the X chromosome^37^, reinforcing our observation here of their proximal binding on crosslinked chromatin.

### Candidates after secondary CRISPR loss-of-function screens

**Fig. 2:**
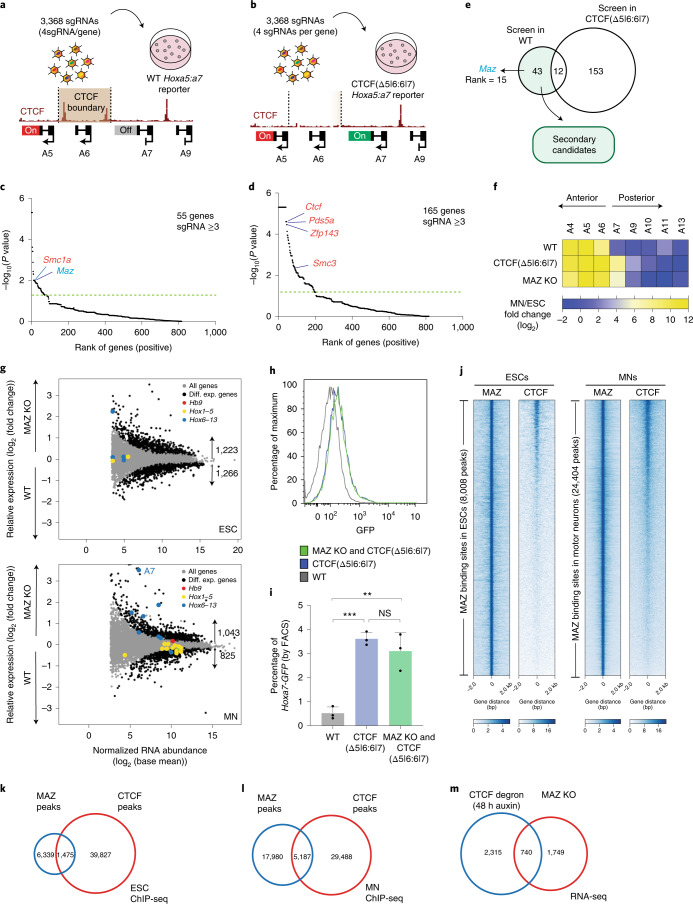
Secondary CRISPR loss-of-function screens and individual validation of MAZ as an insulator-like factor functioning at CTCF boundaries in *Hox* clusters. **a**, Scheme of secondary screen performed in the WT background. **b**, Scheme of secondary screen performed in the CTCF (Δ5|6:6|7) background. **c**, Rank of genes overrepresented in boundary-disrupted MNs versus WT MNs in one biological replicate. Cutoff line indicates *P* < 0.05. The statistics were derived based on MAGeCK tools (Methods). **d**, Rank of genes overrepresented in the dual-positive *Hoxa5:a7* MN population (further disrupted boundary) versus *the Hoxa5-mCherry-*positive population (WT) in two biological replicates. Cutoff line indicates *P* < 0.05. The statistics were derived based on MAGeCK tools (Methods). **e**, Venn diagram depicting overlap of secondary genetic screens in WT versus CTCF(Δ5|6:6|7) background. **f**, Heat map of relative gene expression in WT, CTCF(Δ5|6:6|7) and MAZ KO at the *HoxA* cluster in MNs versus ESCs from three biological replicates. *Maz* KO represents three independent clones. **g**, RNA sequencing (RNA-seq) MA plot of WT versus MAZ KO ESCs (top), and MNs (bottom) from three biological replicates. Differentially expressed (Diff. Exp.) genes are selected as *P* value adjusted < 0.05 using the Wald test built into DESeq2. *Hox* genes in four *Hox* clusters are colored based on their position with respect to the previously demonstrated CTCF boundary in MNs. *Hb9* is an MN marker. **h**, Flow cytometry analysis of MNs with the indicated genotypes: WT, CTCF(Δ5|6:6|7) and MAZ KO & CTCF(Δ5|6:6|7). This plot is one representation of three biological replicates quantified in Fig. 2i (gating of cells is shown in Supplementary Fig. 7a). **i**, Percentage of *Hoxa7-eGFP* cells quantified based on FACS analysis in MNs with the indicated genotypes: WT, CTCF(Δ5|6:6|7) and MAZ KO & CTCF(Δ5|6:6|7). Data are represented as mean values, and error bars indicate standard deviation across three biological replicates. Results from MAZ KO and CTCF(Δ5|6:6|7) represent three independent clones. A two-sided Student’s *t* test (unpaired) was used without multiple-testing correction (black dots represent individual data points; ***P = 0.0002; **P = 0.0052; NS (not significant), *P* = 0.3379). **j**, Heat maps of CTCF and MAZ ChIP-seq read density in ESCs and MNs within a 4-kb window centered on the maximum value of the peak signal. bp, base pair. **k**,**l**, Overlap of CTCF and MAZ binding sites in ESCs and MNs, respectively. ChIP-seq experiments are from one representative of two biological replicates. **m**, Overlap of differentially expressed genes in ESCs upon CTCF degradation^42,43^ and MAZ KO. Differentially expressed genes are selected as *P* value adjusted < 0.05.

**Extended Data Fig. 3:**
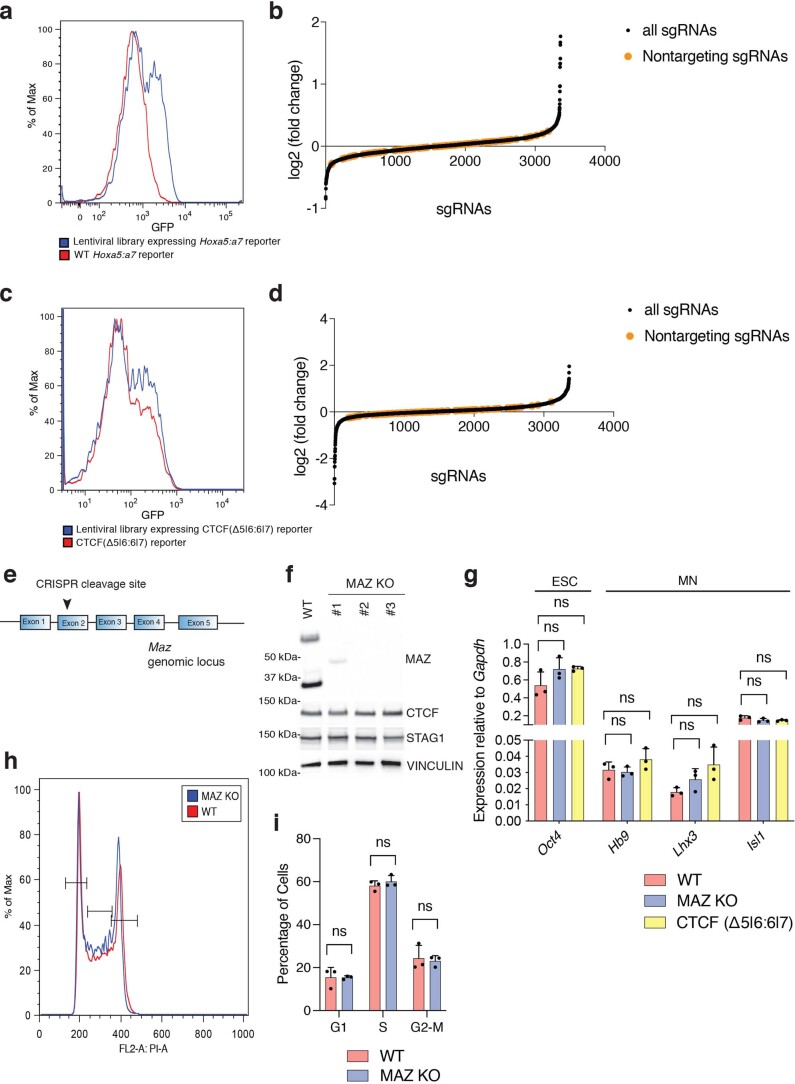
Secondary loss-of-function screen to narrow down candidates. **a**, FACS analysis of GFP expression in lentivirus library expressing MNs in WT background (with intact CTCF binding sites) versus untransduced WT MNs. **b**, Fold change of sgRNAs in the secondary screen performed in WT background. **c**, FACS analysis of GFP expression in lentivirus library expressing MNs in CTCF(Δ5|6:6|7) background versus untransduced CTCF(Δ5|6:6|7) MNs. **d**, Fold change of sgRNAs in the secondary screen performed in CTCF(Δ5|6:6|7) background. **e**, Strategy for generating the MAZ KO ESC line via CRISPR. **f**, Western blot analysis of indicated proteins in WT and MAZ KO ESCs. Two bands likely indicate two isoforms for MAZ. The large isoform (~60 kDa) has been described previously^40^, while the small isoform (~30 kDa) was detected in this study upon analysis of MAZ KO. **g**, RT-qPCR analysis for the indicated ESC and MN markers in WT, MAZ KO, and CTCF(Δ5|6:6|7) cells. RT-qPCR signal is normalized to *Gapdh* levels. RT-qPCR results are represented as mean values and error bars indicating SD across three biological replicates (two-sided Student’s *t*-test without multiple testing correction; black dots: individual data points). *Maz* KO represents three independent clones. **h**, Cell cycle analysis by FACS performed in WT versus MAZ KO ESCs (see Supplementary Fig. 7b for gating of cells). **i**, Quantification of cell cycle analysis by FACS in WT versus MAZ KO ESCs (see Supplementary Fig. 7b for gating of cells). Data are represented as mean values and error bars indicating SD across three biological replicates (two-sided Student’s *t*-test without multiple testing correction; black dots: individual data points). *Maz* KO represents three independent clones. Source data

Both the genetic and biochemical approaches revealed a large list of candidates, which were further narrowed down and validated through independent secondary genetic screens. In order to systematically narrow down candidates from the primary genome-wide screens (Supplementary Dataset 2) and check whether CTCF partners identified in Fig. 1g and Extended Data Fig. 2c (Supplementary Dataset 4) have a role at the CTCF boundary at the *HoxA* cluster, we performed secondary loss-of-function screens with a small custom library (Supplementary Dataset 6, Extended Data Fig. 2h and Supplementary Note 4). Importantly, these secondary screens were performed with increased statistical power in ESCs having either the WT *Hoxa5:7* reporter (Fig. 2a and Extended Data Fig. 3a,b) or the CTCF (Δ5|6:6|7) *Hoxa5:7* reporter (Fig. 2b and Extended Data Fig. 3c,d) to focus on candidates uniquely impacting the CTCF boundary in the WT background. Based on the rank of genes overrepresented in the *Hoxa5:7* dual-positive MN population compared to *Hoxa5*-positive cells, we identified 55 genes that disrupt the CTCF boundary in the WT background having intact CTCF binding sites (Fig. 2c and Supplementary Dataset 7). Similarly, we identified 165 genes that influence CTCF-boundary/*Hoxa7* gene expression from screens performed in the CTCF (Δ5|6:6|7) background (Fig. 2d and Supplementary Dataset 8). Thus, the secondary screens resulted in a small list of 43 genes, which when mutated phenocopied the CTCF (Δ5|6) motif deletion in the presence of intact CTCF binding sites (Fig. 2e shows a comparison of secondary screens in both backgrounds; Supplementary Dataset 9). Importantly, the secondary screens also confirmed the identification of *Maz* uniquely in the WT background. Other genes shown in Fig. 2c,d are expected positive controls such as *Ctcf*, cohesin components/accessory subunits and *Znf143*, which encodes a protein that colocalizes with CTCF at TADs^38,39^ (Supplementary Note 4).

### Validation of MAZ function at CTCF boundaries in *Hox* clusters

**Extended Data Fig. 4:**
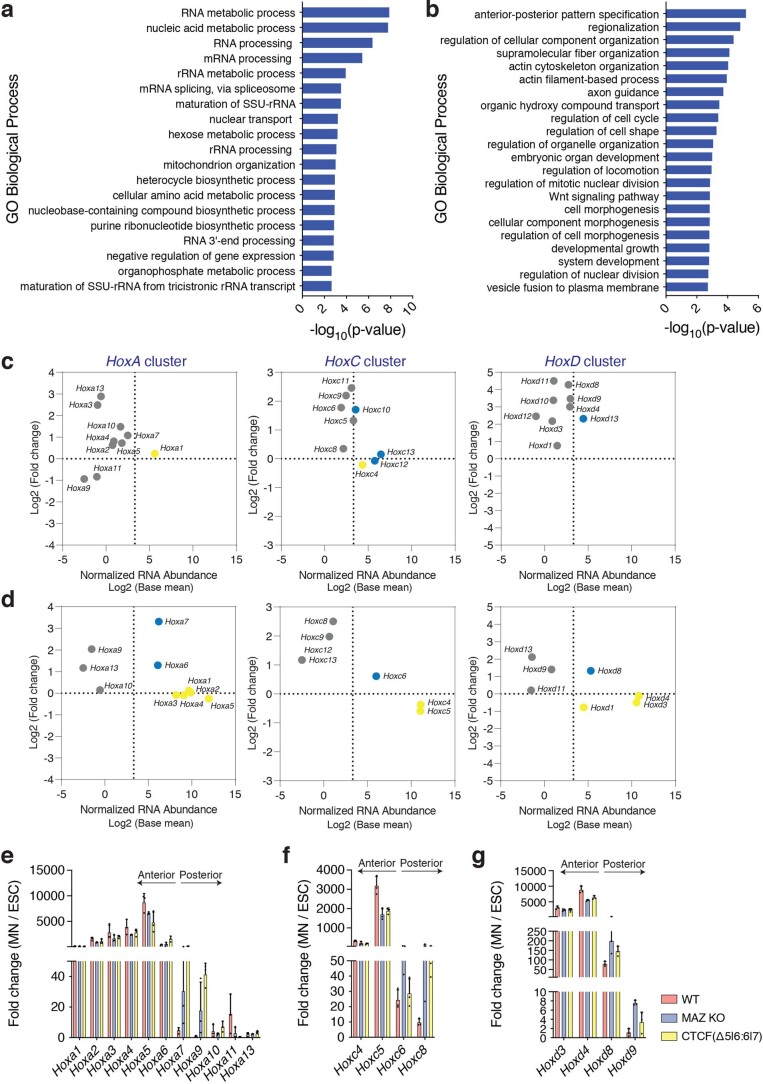
*Maz* loss in MNs engenders features of CTCF-boundary disruption. **a**, GO analysis of biological processes in differentially expressed genes in MAZ KO ESCs compared to WT ESCs. PANTHER overrepresentation test tools were used (see Methods) and GO processes with a fold enrichment > 2 were presented (FDR < 0.05). **b**, GO analysis of biological processes in differentially expressed genes in MAZ KO MNs compared to WT MNs. PANTHER overrepresentation test tools were used (see Methods) and GO processes with a fold enrichment > 2 were presented (FDR < 0.05). **c**, RNA-seq MA plots of WT versus MAZ KO ESCs at the *HoxA, HoxC*, and *HoxD* clusters. **d**, RNA-seq MA plots of WT versus MAZ KO MNs at the *HoxA, HoxC*, and *HoxD* clusters. RNA-seq results represent three biological replicates. *Hox* genes with RNA abundance ≥ 10 are represented in colors, and the rest are represented in gray (see Supplementary Data 10-11). *Hox* genes in 3 *Hox* clusters are colored based on their position with respect to the previously demonstrated CTCF-boundary in MNs. **e**, RT-qPCR analysis for the indicated *Hox* genes in *HoxA* cluster, *HoxC* cluster (**f**), and *HoxD* cluster (**g**) in WT, MAZ KO, and CTCF(Δ5|6:6|7) cells. RT-qPCR signal is normalized to *Gapdh* levels. Fold change in expression in MNs is calculated relative to baseline expression in ESCs. All RT-qPCR results are represented as mean values and error bars indicating SD across three biological replicates. *Maz* KO represents three independent clones. Supplementary Data 12 shows raw data for RT-qPCR and the comparison for each gene in WT versus MAZ KO and WT versus CTCF(Δ5|6:6|7) in both ESCs and MNs.

Among the candidates we identified as mimicking CTCF (Δ5|6) at the *HoxA* cluster, MAZ was ranked high in multiple primary screens, identified as a colocalizing factor with CTCF on chromatin and further validated through secondary screens. MAZ is a ubiquitously expressed protein that was initially identified as a regulatory protein associated with *Myc* gene expression^40^ and also identified as a regulatory factor for the insulin promoter^41^. To validate the screen results, we generated a MAZ KO in ESCs through CRISPR editing^19^ (Extended Data Fig. 3e,f). The MAZ KO did not produce a profound change in gene markers associated with ESC and MN fate (Extended Data Fig. 3g). In addition, the MAZ KO did not result in cell cycle changes in ESCs (Extended Data Fig. 3h,i). Importantly, the MAZ KO did not affect overall CTCF and cohesin levels (Extended Data Fig. 3f). However, as shown in Fig. 2f, the MAZ KO in MNs mimicked the specific deletion of the CTCF sites (Δ5|6:6|7) at the *HoxA* cluster and disrupted the boundary between active and repressed genes. In addition, the MAZ KO resulted in differential expression of ~2,400 genes in ESCs (Fig. 2g, top; Extended Data Fig. 4a for Gene Ontology (GO) analysis and Supplementary Dataset 10) and ~1,800 genes in MNs compared to WT (Fig. 2g, bottom, and Supplementary Dataset 11). GO analysis indicated that developmental processes, particularly anterior–posterior pattern specification, are enriched in MAZ KO MNs compared to WT MNs (Extended Data Fig. 4b). Consistent with MAZ having a role in CTCF-boundary integrity and the MAZ KO mimicking the CTCF binding site deletions, the MAZ KO led to a derepression of mainly posterior *Hox* genes after CTCF boundaries in MNs, but not in ESCs with the exception of *Hoxc10* and *Hoxd13* (Fig. 2g, Extended Data Fig. 4c–g and Supplementary Datasets 10–12). Notably, we did not observe further derepression of *Hoxa7-eGFP* upon differentiation of ESCs into MNs when comprising both CTCF (Δ5|6:6|7) and a MAZ KO (Fig. 2h,i), confirming our secondary screen results (Fig. 2c–e).

### MAZ colocalizes with CTCF on chromatin

**Fig. 3:**
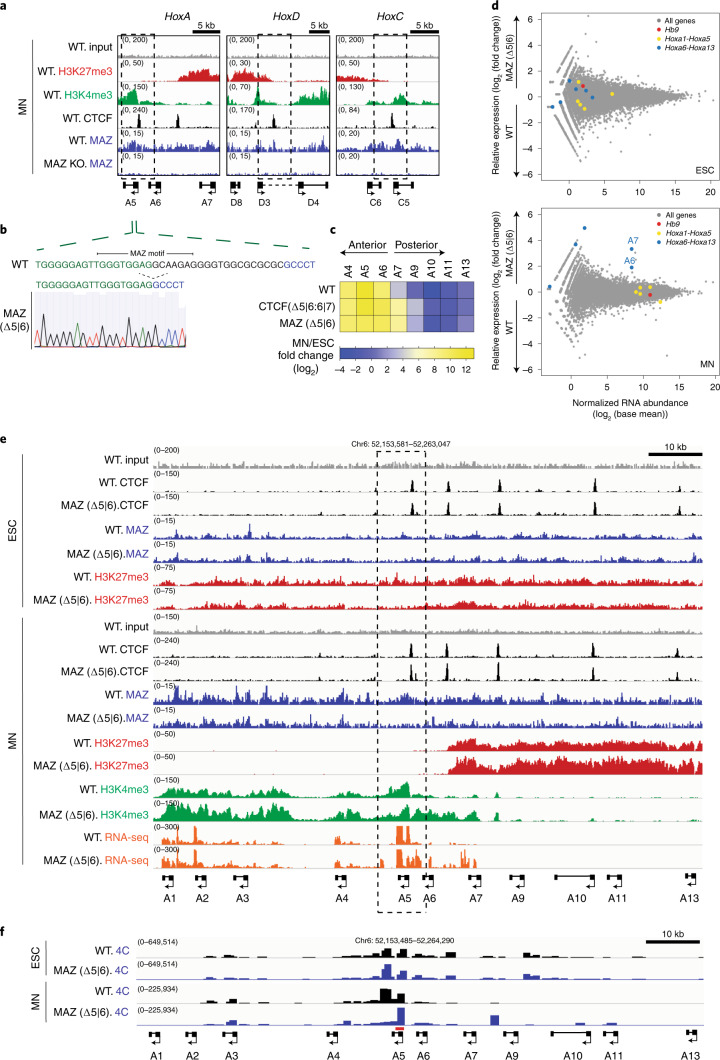
Loss of the MAZ binding site alters *Hox* gene expression pattern, chromatin domains and topological organization at *Hox* clusters. **a**, ChIP-seq for H3K27me3, H3K4me3, CTCF and MAZ in WT MNs and ChIP-seq for MAZ in MAZ KO MNs in the chromatin boundary of *HoxA, HoxD* and *HoxC* clusters. **b**, MAZ binding site deletion via CRISPR is depicted for the 5|6 site at the *HoxA* cluster. **c**, Heat map of relative gene expression in WT, CTCF(Δ5|6:6|7) and MAZ (Δ5|6) at the *HoxA* cluster in MNs versus ESCs from three biological replicates. **d**, RNA-seq MA plot of WT versus MAZ (∆5|6) ESCs (left) and MNs (right) from three biological replicates. *HoxA* genes are colored based on their position with respect to the previously demonstrated CTCF boundary in MNs. *Hb9* is an MN marker. Differentially expressed genes are selected as *P* value adjusted < 0.05 using the Wald test built into DESeq2 (Supplementary Datasets 13 and 14). **e**, ChIP-seq for CTCF, MAZ, indicated histone modifications and RNA-seq tracks in WT and MAZ (∆5|6) ESCs and MNs in the *HoxA* cluster. ChIP-seq tracks are from one representative of two biological replicates for CTCF and MAZ and one replicate for the histone modifications. RNA-seq tracks are from one representative of three biological replicates. **f**, 4C contact profiles in WT versus MAZ (Δ5|6) ESCs and MNs using a viewpoint shown in red at indicated region at *Hoxa5*. One representative of three biological replicates is shown for all except for two replicates for WT MNs.

**Extended Data Fig. 5:**
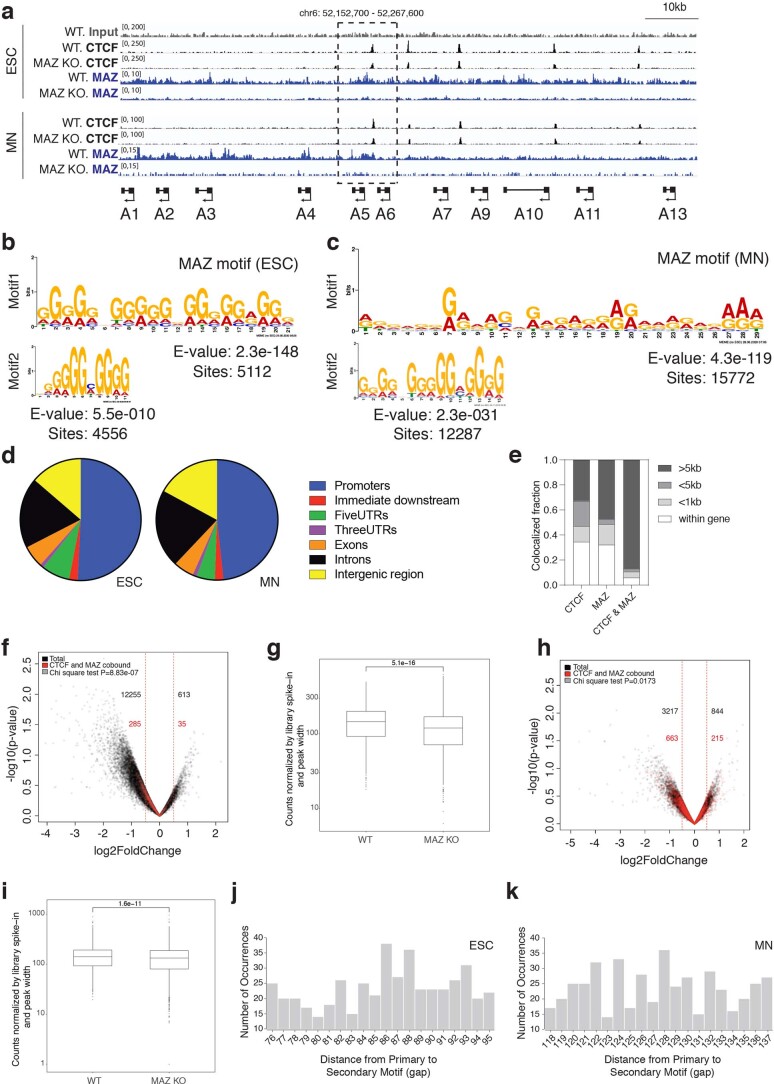
Global alteration of CTCF binding upon MAZ KO. **a**, ChIP-seq for CTCF or MAZ in WT and MAZ KO ESCs and MNs in the *HoxA* cluster. **b**, Motif analysis of MAZ ChIP-seq in ESCs, and MNs (**c**) by using MEME. Top two motifs are shown with the number of MAZ binding sites where the motifs are detected in each cell type. **d**, Distribution of MAZ binding sites across genomic features. **e**, Analysis of CTCF and MAZ occupancy at commonly impacted genes in MAZ KO and CTCF-degron ESCs. **f**, Volcano plot showing the magnitude of change in CTCF binding in WT versus MAZ KO ESCs. The number of peaks was counted with the cutoff of ±0.5 log_2_(fold change). All CTCF binding sites are shown in black and CTCF-MAZ co-bound sites are shown in red (n = 3 biologically independent experiments). **g**, Boxplot demonstrating the normalized counts of CTCF ChIP-seq in WT versus MAZ KO in ESCs (n = 3 biologically independent experiments), *P* = 5.1e-16. *P* value is shown for unpaired one-sided Wilcoxon rank sum test. The center bar displays the median value, and the box boundaries were drawn at the 25th and the 75th percentiles, respectively. Whiskers were defined by 1.5 times the interquartile range from the box. **h**, Volcano plot showing the magnitude of change in CTCF binding in WT versus MAZ KO MNs. The number of peaks was counted with the cutoff of ±0.5 log_2_(fold change). All CTCF binding site are shown in black and CTCF-MAZ co-bound sites are shown in red (n = 3 biologically independent experiments). **i**, Boxplot demonstrating the normalized counts of CTCF ChIP-seq in WT versus MAZ KO in MNs (n = 3 biologically independent experiments), *P* = 1.6e-11. *P* value is shown for unpaired one-sided Wilcoxon rank sum test. The center bar, the box boundaries, and whiskers are as in **g**. **j**, Analysis of distance from CTCF motif (primary) to the MAZ motif (secondary) within a 500 bp window centered at CTCF peaks in ESCs and MNs (**k**) by using SpaMo tools.

**Extended Data Fig. 6:**
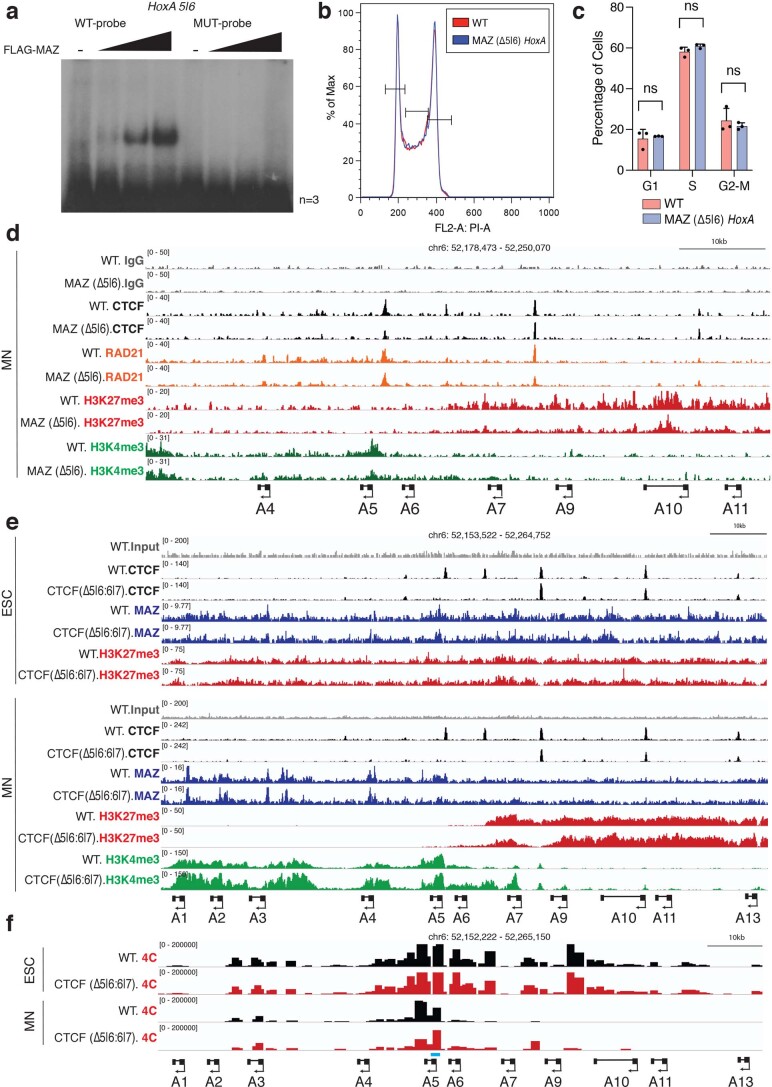
Loss of MAZ motifs perturbs CTCF-boundary features at *HoxA*. **a**, EMSA assay indicating FLAG-MAZ binding to MAZ motifs at *Hoxa5*|*6* MAZ binding sites in WT probes in comparison to mutant (MUT) probes. One representative of three biological replicates is shown. **b**, FACS analysis of cell cycle in WT versus MAZ (∆5|6) ESCs (see Supplementary Fig. 7b for gating of cells). **c**, Quantification of cell cycle analysis by FACS in WT versus MAZ (∆5|6) ESCs (see Supplementary Fig. 7b for gating of cells). Data are represented as mean values and error bars indicating SD across three biological replicates. Two-sided Student’s *t*-test (unpaired) was used without multiple testing correction (black dots: individual data points). **d**, Normalized CUT&RUN signals for CTCF, RAD21, and indicated histone modifications in the *HoxA* cluster in WT versus MAZ (∆5|6) MNs that were sorted for double-positives (*Hoxa5-P2A-mCherry* and *Hoxa7-P2A-eGFP*). CUT&RUN tracks are from one representative of two biological replicates for H3K4me3, and one replicate for others**. e**, Normalized ChIP-seq densities for CTCF, MAZ, and indicated histone modifications in WT and CTCF (∆5|6:6|7) ESCs and MNs in the *HoxA* cluster. ChIP-seq tracks are from one representative of two biological replicates for all except for one replicate for MAZ, and histone modifications in CTCF (∆5|6:6|7) ESCs and MNs. **f**, 4C contact profiles in WT versus CTCF (Δ5|6:6|7) ESCs and MNs, using a viewpoint shown in blue at indicated region at *Hoxa5*. One representative of three biological replicates is shown for all except for two replicates for WT MNs. Source data

**Extended Data Fig. 7:**
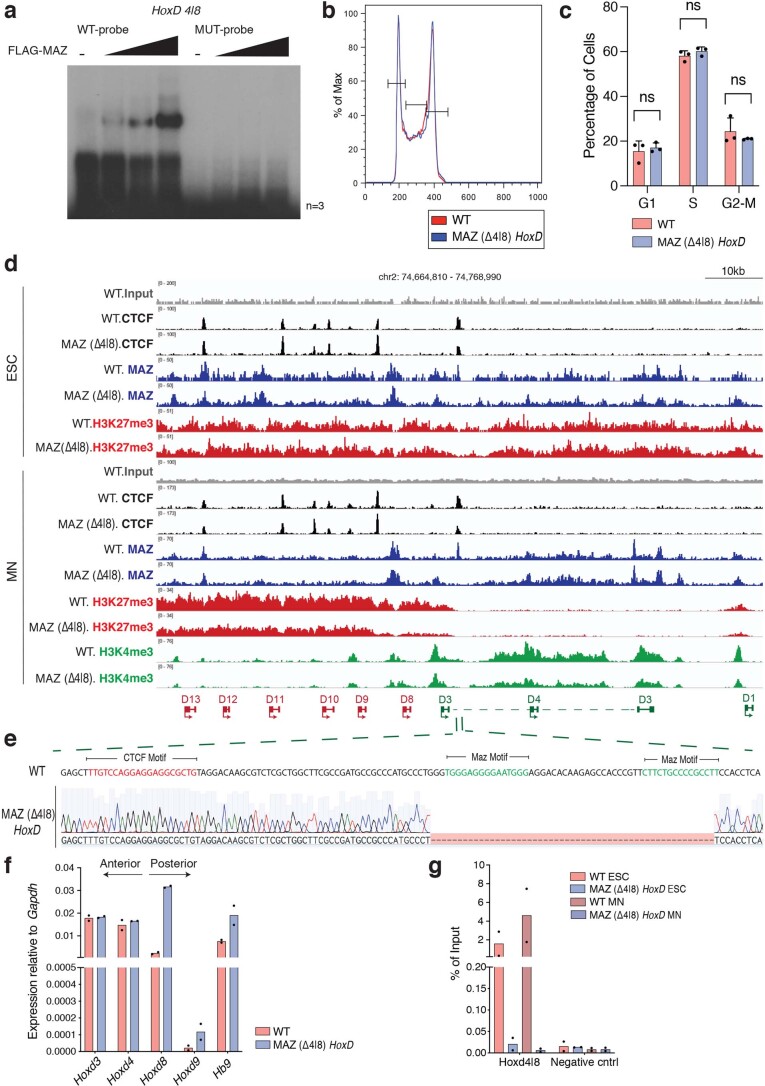
Loss of MAZ motif alters *Hox* gene expression and *HoxD* chromatin domains. **a**, EMSA assay for FLAG-MAZ binding to MAZ motifs at *Hoxd4*|*8* MAZ binding sites in WT probes in comparison to mutant (MUT) probes. One representative of three biological replicates is shown. **b**, FACS analysis of cell cycle in WT versus MAZ (∆4|8) ESCs (see Supplementary Fig. 7b for gating of cells). **c**, Quantification of cell cycle analysis by FACS in WT versus MAZ (∆4|8) ESCs (see Supplementary Fig. 7b for gating of cells). Results are represented as mean values and error bars indicating SD across three biological replicates. Two-sided Student’s *t*-test (unpaired) was used without multiple testing correction (black dots: individual data points). **d**, Normalized ChIP-seq densities for CTCF, MAZ, and indicated histone post-translational modifications (PTMs) in WT, and MAZ (∆4|8) ESCs and MNs in the *HoxD* cluster. ChIP-seq tracks are from one representative of two biological replicates for CTCF, and one replicate for MAZ and histone PTMs. **e**, MAZ binding site deletion via CRISPR is depicted for the 4|8 site at the *HoxD* cluster. **f**, RT-qPCR for the indicated *Hox* genes in the *HoxD* cluster in MNs upon MAZ (∆4|8). Results are represented as mean values of two independent experiments with individual data points overlaid. **g**, MAZ ChIP-qPCR analysis in the *HoxD* cluster in mESCs and MNs upon MAZ (∆4|8). Results are represented as mean values of two independent experiments with individual data points overlaid. Source data

Based on our ChIP-seq analysis, ~20% of MAZ binding sites colocalize with CTCF in ESCs and MNs (Fig. 2j–l). The MAZ signal is specific given its loss in MAZ KO cells (Extended Data Fig. 5a and Supplementary Fig. 1) and the de novo detection of the MAZ motif within its binding sites in ESCs and MNs (Extended Data Fig. 5b,c). MAZ mostly binds to promoters in addition to introns, intergenic regions and other regions (Extended Data Fig. 5d). That CTCF and MAZ functionally cooperate beyond the *Hox* clusters is supported by our findings that 740 genes are commonly impacted when comparing differentially expressed genes reported in the case of CTCF loss (auxin treatment, 48 h) in ESCs^42,43^ and those in the case of MAZ loss (Fig. 2m and Extended Data Fig. 5e for CTCF and MAZ occupancies at these genes). As we initially identified the MAZ KO as influencing the CTCF boundary at the *HoxA* cluster (Fig. 2f), we compared ChIP-seq tracks of MAZ at the *HoxA* cluster to those of CTCF. MAZ appears to bind to DNA in proximity to CTCF as MAZ and CTCF colocalized at CTCF borders in *Hox* clusters (Fig. 3a; Fig. 3e and Extended Data Figs. 5a and 6 for *HoxA*; and Extended Data Fig. 7 for *HoxD*). MAZ KO in ESCs and MNs resulted in a slight decrease in CTCF binding at the boundary in the *HoxA* cluster (Extended Data Fig. 5a). We also observed a similar global decrease in CTCF binding in the MAZ KO (Extended Data Fig. 5f–i), suggesting a possible role of MAZ in stabilizing CTCF on chromatin (Supplementary Note 5).

### Repercussions of MAZ motif deletion at the *Hox* clusters

MAZ binds to a GC-rich motif on DNA (GGGAGGG) through its zinc fingers^44^ (Extended Data Fig. 5b,c shows MAZ motifs detected in ESCs and MNs). The distance analysis between MAZ and CTCF motifs indicates ~70–140 bp within a window of 500 bp centered on CTCF binding regions in ESCs and MNs (Extended Data Fig. 5j,k). We found MAZ binding motifs close to CTCF at the *Hox* boundaries (Fig. 3a), which were confirmed as such through FLAG-MAZ binding in vitro (Extended Data Figs. 6a and 7a). We also tested whether deletion of MAZ binding motifs at a specific *Hox* cluster mimics that of a CTCF binding site in the respective *Hox* cluster (Supplementary Note 6). As expected, MAZ binding site deletions at *Hox* clusters did not influence the cell cycle in ESCs (Extended Data Fig. 6b,c for *HoxA* and Extended Data Fig. 7b,c for *HoxD* clusters). Interestingly, MAZ binding site deletions generated at *Hoxa5*|*6* (Fig. 3b, Extended Data Fig. 6 and Supplementary Fig. 2) and *Hoxd4*|*8* (Extended Data Fig. 7) phenocopied the transcriptional repercussions of CTCF binding site deletions at the respective boundaries without loss of CTCF binding at the boundary (Fig. 3c–e, Extended Data Fig. 6d,e and Supplementary Datasets 13 and 14 for *HoxA;* Extended Data Fig. 7d–f for *HoxD;* and published results for the loss of the CTCF boundary^16^). These results pointed to a specific role of MAZ binding in regulating *Hox* gene expression at CTCF boundaries in multiple *Hox* clusters during differentiation (Supplementary Note 6). When we analyzed how chromatin domains were influenced upon deletion of the MAZ binding site at the *Hoxa5*|*6* boundary, we observed spreading of active chromatin (H3K4me3) into the repressed region (H3K27me3) at the boundary (Fig. 3e and Extended Data Fig. 6d), similar to the CTCF binding site deletions shown in Extended Data Fig. 6e and that reported previously^16^. In accordance, CTCF depletion was also reported to impact transcriptional activity, but not the spread of H3K27me3 domains^42^. Similar to MAZ (Δ5|6) being ineffectual with respect to neighboring CTCF binding (Fig. 3e), CTCF (Δ5|6:6|7) did not affect adjacent MAZ binding at the *HoxA* cluster (Extended Data Fig. 6e). Nevertheless, we note that based on cleavage under targets and release using nuclease (CUT&RUN) analysis of the double-positive sorted population (*Hoxa5-P2A-mCherry* and *Hoxa7-P2A-eGFP*) in MNs, MAZ (Δ5|6) did not affect RAD21 binding, yet it modestly decreased CTCF binding and H3K27me3 (Extended Data Fig. 6d). Although H3K4me3 spreading (Fig. 3e) and decreased H3K27me3 were observed for MAZ *Hoxa5*|*6* motif deletion (Extended Data Fig. 6d), the MAZ motif deletion at *Hoxd4*|*8* exhibited only decreased H3K27me3 (Extended Data Fig. 7d). Thus, our results suggest that MAZ acts as a chromatin border factor alone, being partially additive with CTCF, and that alterations of the active and repressive chromatin marks can be context dependent.

According to the analysis of topological organization by circular chromosome conformation capture (4C), the interaction signal covers the *HoxA* cluster in ESCs as a single architectural domain not altered upon MAZ deletion (Δ5|6) (Fig. 3f), in accordance with the CTCF motif deletion shown in Extended Data Fig. 6f, and as reported previously^16^. However, upon differentiation into MNs, the *HoxA* cluster partitions into active and repressed regions (Fig. 3e)^16^, as reflected by the 4C interactions observed exclusively within the rostral part of the *HoxA* cluster (Fig. 3f). In contrast to ESCs, deletion of the MAZ *Hoxa5*|*6* binding site affects the topological organization of the *HoxA* cluster in MNs (Fig. 3f), similar to that observed for CTCF(Δ5|6:6|7) in MNs (Extended Data Fig. 6f), and as reported previously^16^. Thus, MAZ (Δ5|6) impacts not only the partitioning of active and repressed chromatin domains and *Hox* gene expression, but also the structural organization of the *HoxA* cluster.

### Effect of MAZ depletion on global genome organization

**Fig. 4:**
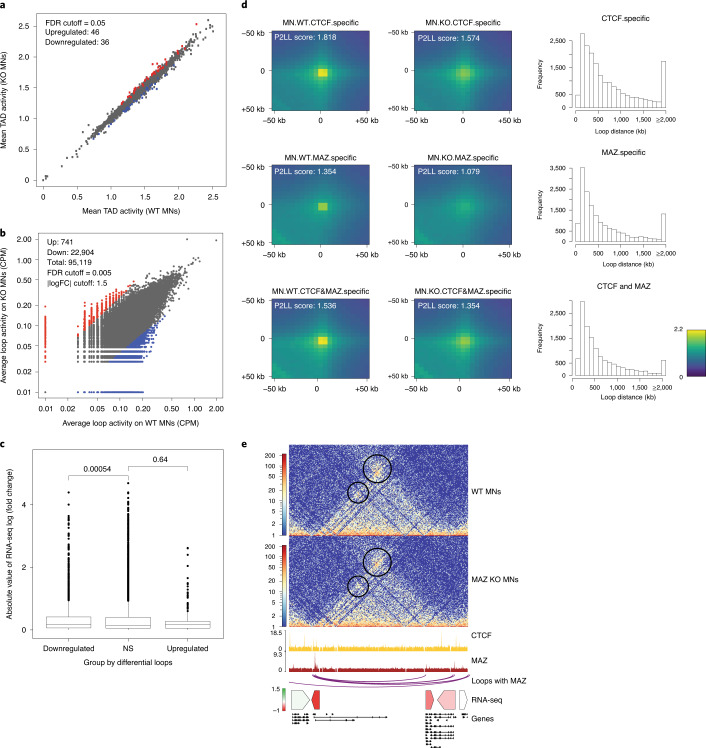
Effect of MAZ on global genome organization. **a**, Scatter plot showing differential intra-TAD activity in WT versus MAZ KO MNs (FDR cutoff = 0.05). Down, downregulated; Up, upregulated. **b**, Scatter plot showing differential loop activity in WT versus MAZ KO MNs (all loops, *n* = 95,119, FDR cutoff = 0.005, | log (fold change) | cutoff = 1.5, upregulated = 741, downregulated: 22,904). CPM, counts per million reads mapped. **c**, Boxplot of absolute value of RNA-seq log (fold change) of genes within the differential loops (down-/upregulated) versus nonsignificant (NS) loops in WT versus MAZ KO MNs. *P* values are shown for unpaired one-sided Wilcoxon rank sum tests. The median is shown at the center line, and the whisker extends up to 1.5 times the interquartile range using the default parameters. **d**, APA of loops in WT versus MAZ KO MNs showing ChIP-seq signals of CTCF, MAZ or both at any region covered by them. The resolution of APA is 5 kb. Histograms show the distribution of loop distance in MAZ KO compared to WT related to the binding level of ChIP-seq. P2LL, (Peak to Lower Left) is the ratio of the central pixel to the mean of the mean of the pixels in the lower left corner. **e**, Visualization of Hi-C contact matrices for a zoomed-in region around the *Shh* locus in WT versus MAZ KO MNs. Shown below are ChIP-seq read densities for CTCF and MAZ, loops with MAZ at both anchors in MNs, heat map of RNA-seq log_2_ (fold change) of underlying genes and all gene annotations.

**Extended Data Fig. 8:**
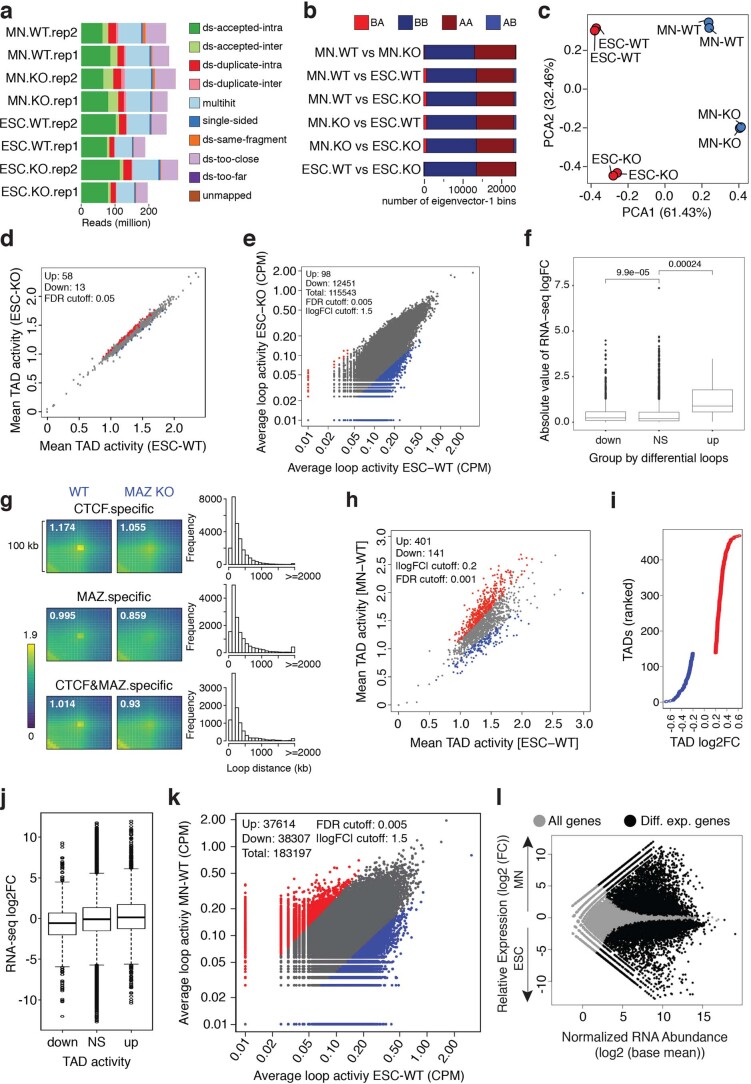
Global genome organization as a function of MAZ KO or differentiation. **a**, Bar plot of Hi-C read counts across the samples. **b**, Bar plot of compartment switches between active (A) and inactive (B) compartments in ESCs and MNs. **c**, Principal Component Analysis (PCA) of boundary scores in WT ESCs, MAZ KO ESCs, WT MNs, and MAZ KO MNs. **d**, Scatter plot showing differential intra-TAD activity in WT versus MAZ KO ESCs (FDR cutoff = 0.05). Down, downregulated; Up, upregulated. **e**, Scatter plot showing differential loop activity in WT versus MAZ KO ESCs (all loops, n = 115,543, FDR cutoff = 0.005, | log (fold change) | cutoff = 1.5, Upregulated: 98, Downregulated: 12,451). Down, downregulated; Up, upregulated. **f**, Boxplot of absolute value of RNA-seq log (fold change) of genes within the differential loops (down/up) versus nonsignificant (NS) loops in WT versus MAZ KO ESCs. *P* values are shown for unpaired one-sided Wilcoxon rank sum test. The median is shown at the center line and the whisker extends up to 1.5 times the interquartile range, using the default parameters (n = 3 and n = 2 biologically independent experiments for RNA-seq and Hi-C, respectively). Down, downregulated; Up, upregulated. **g**, APA of loops in WT versus MAZ KO ESCs showing ChIP-seq signals of CTCF, MAZ, or both at any occupied regions. The resolution of APA is 5 kb. The APA score is reported on top of each plot. Histograms show the distribution of loop distance in MAZ KO compared to WT related to the binding level of ChIP-seq. **h**, Scatter plot showing differential intra-TAD activity in WT ESC versus MNs (FDR cutoff = 0.001, log (fold change) cutoff = 0.2). Down, downregulated; Up, upregulated. **i**, TADs (n = 467) ranked by TAD activity change in ESCs versus MNs. **j**, Boxplot of RNA-seq log_2_(fold change) in TADs with up/downregulated activity compared to nonsignificant (NS) activity in ESCs versus MNs (n = 3 and n = 2 biologically independent experiments for RNA-seq and Hi-C, respectively). The center bar displays the median value and the box boundaries were drawn at the 25th and the 75th percentiles, respectively. Whiskers were defined by 1.5 times the interquartile range from the box. Down, downregulated; Up, upregulated. **k**, Scatter plot showing differential loop activity in WT ESCs versus MNs (all loops, n = 183,197, FDR cutoff = 0.005, | log (fold change) | cutoff = 1.5, Upregulated: 37,614, Downregulated: 38,307). Down, downregulated; Up, upregulated. **l**, RNA-seq MA plot of WT ESCs versus MNs from three biological replicates. Differentially expressed genes are selected as *P* value adjusted (padj) < 0.001 by using the Wald test built into DEseq2.

**Extended Data Fig. 9:**
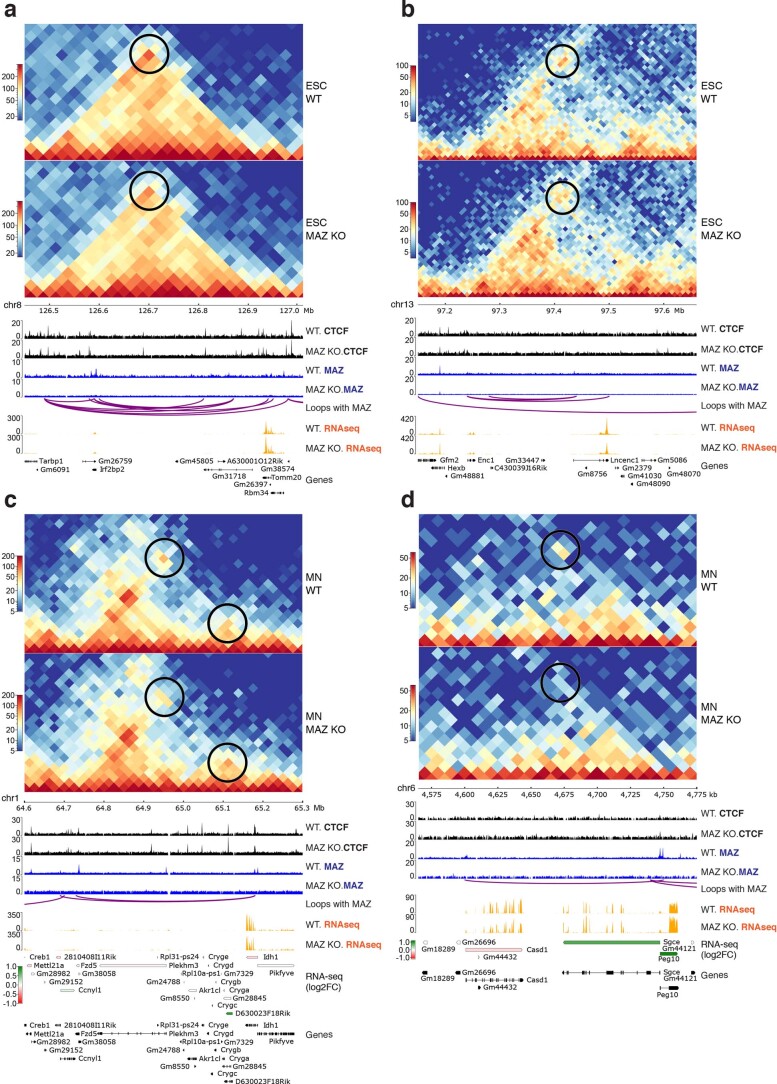
Visualization of Hi-C analysis at the indicated regions in WT versus MAZ KO ESCs and MNs. **a**, Visualization of Hi-C contact matrices in WT versus MAZ KO for zoomed-in regions around *Rbm34* on chromosome 8 in ESCs, *Enc1* on chromosome 13 in ESCs (**b**), *Idh1* on chromosome 1 in MNs (**c**), and *Peg10* on chromosome 6 in MNs (**d**). Shown below are ChIP-seq read densities for CTCF and MAZ, loops with MAZ at both anchors, RNA-seq tracks, heat map of the relative expression (log_2_ fold change) in WT versus MAZ KO for the underlying genes, and all gene annotations. Black circles highlight alterations in loop densities.

**Extended Data Fig. 10:**
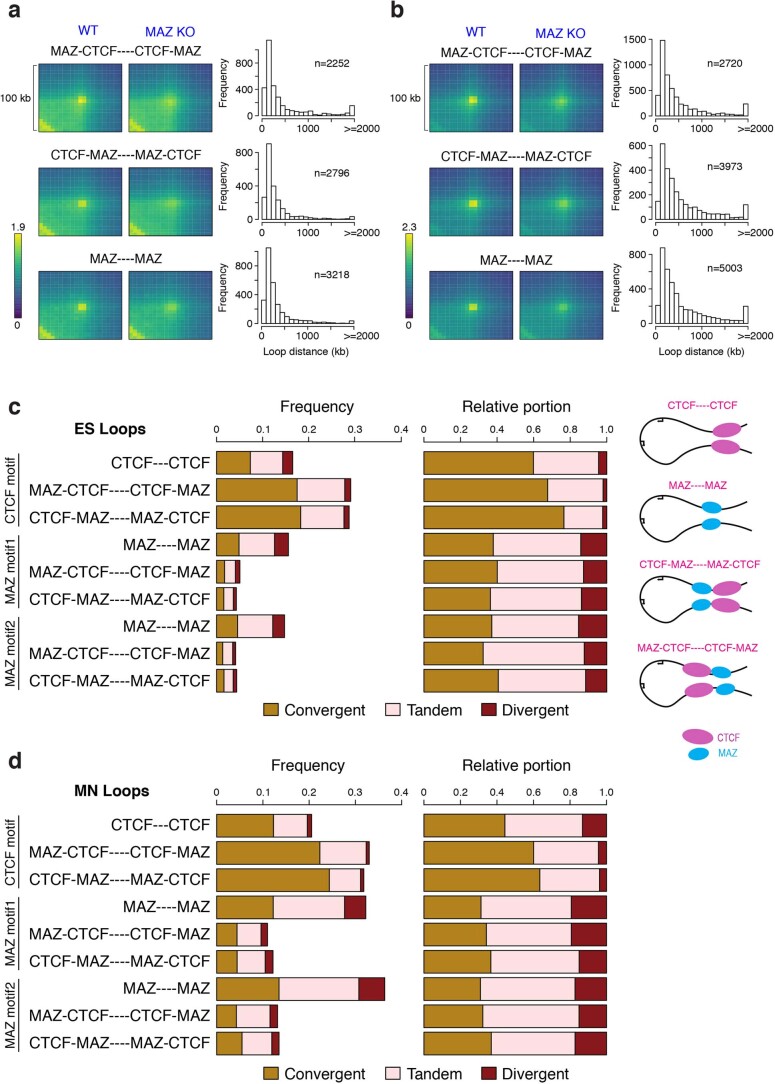
Directionality of CTCF and MAZ motifs at loop anchors. **a**, APA of loops in WT versus MAZ KO ESCs and MNs (**b**) showing ChIP-seq signals of CTCF & MAZ at both anchors (CTCF being towards the inside of the loop), CTCF & MAZ at both anchors (MAZ being towards the inside of the loop), and MAZ at both loop anchors. The resolution of APA is 5 kb. Histograms show the distribution of loop distance in MAZ KO compared to WT related to the binding level of ChIP-seq. **c**, Bar graph showing the distribution of convergent, divergent, and tandem motifs for CTCF and MAZ at loop anchors in Hi-C in ESCs and MNs (**d**). The two different MAZ motifs analyzed are the most enriched motifs based on ChIP-seq (see Extended Data Fig. 5b,c). The x-axis of the bar plot is first shown as the frequency over all the loops (left) and secondly, as the proportion of the three orientations when cases with no motif hits were excluded (middle). The analyzed orientations of CTCF and MAZ motifs at loop anchors are depicted schematically on the right.

Besides its boundary role at *Hox* clusters, CTCF plays a pleiotropic role in three-dimensional (3D) genome structure. As shown here, MAZ colocalizes with CTCF at ~20% of MAZ binding sites in ESCs and MNs (Fig. 2j–l), and MAZ KO reduces CTCF binding (Extended Data Fig. 5f–i) and results in differential expression of ~2,000 genes (Fig. 2g). Thus, we examined the effect of MAZ KO on global 3D genomic organization by performing Hi-C in WT versus MAZ KO ESCs and MNs in biological duplicates (Fig. 4, Extended Data Figs. 8 to 10 and Supplementary Note 7). MAZ depletion resulted in a modest alteration of local contacts within TADs in ESCs (Extended Data Fig. 8d) and MNs (Fig. 4a), indicating its contribution to TAD integrity. In addition, analysis of differential loop activity showed alterations upon MAZ KO in both cell types (Fig. 4b and Extended Data Fig. 8e), indicating a global reduction in loop interactions relative to WT. Such loop changes were accompanied by significant alterations in the expression of genes that localize within these differential loops in both ESCs (Extended Data Fig. 8f) and MNs (Fig. 4c). In accordance, aggregate peak analysis (APA) showed that contact frequencies were decreased in MAZ KO ESCs (Extended Data Fig. 8g) and MNs (Fig. 4d) compared to WT and that the majority of loops examined had less than 2 Mb between the anchors. Interestingly, loops having CTCF, MAZ or CTCF and MAZ at loop anchors exhibited decreased contact frequencies upon MAZ KO (Fig. 4d and Extended Data Fig. 8g). These decreased signals observed at loop anchors containing CTCF (Fig. 4d and Extended Data Fig. 8g, top and bottom plots) could be attributable to the global decrease in CTCF binding in the absence of MAZ (Extended Data Fig. 5f–i). In particular, upon MAZ KO, we observed a mild downregulation of MAZ-containing loops in the *Shh* locus accompanied by its downregulation (Fig. 4e and Supplementary Dataset 11; and Extended Data Fig. 9, Supplementary Figs. 3–5 and Supplementary Note 7 for other loci). We also observed a larger-scale effect on intra-TAD activity and looping interactions accompanied by gene expression changes upon differentiation of ESCs into MNs (Extended Data Fig. 8h–l). As CTCF motifs are known to be convergent at loop anchors^45–47^, we analyzed the directionality for the CTCF motif and MAZ motifs shown in Extended Data Figure 5b,c. Both ESCs and MNs exhibited MAZ towards the inside of the loop with respect to CTCF in a slightly higher number of loop anchors (Extended Data Fig. 10a,b). Moreover, the convergence observed for CTCF and MAZ motifs at Hi-C loop anchors in Extended Data Fig. 10c,d demonstrated that MAZ motifs can be mostly convergent and tandem at loop anchors; however, the frequency of convergence observed for MAZ is smaller than that for CTCF. Collectively, these results demonstrate that MAZ participates in the maintenance of local interactions within the TADs and other looping interactions.

### RAD21 relocalization to MAZ binding sites upon loss of CTCF

**Fig. 5:**
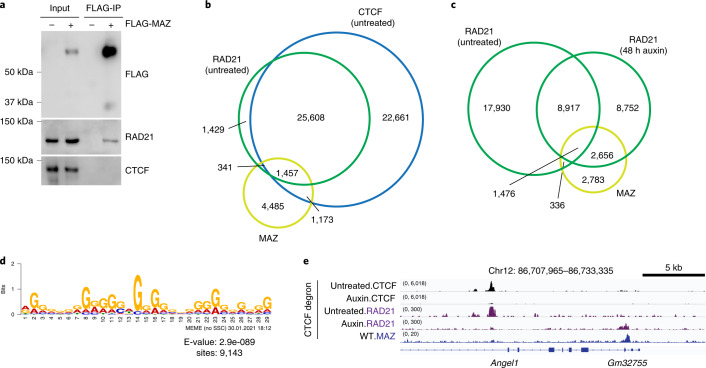
MAZ interaction with RAD21 and relocalization of RAD21 to MAZ binding sites in the absence of CTCF. **a**, Western blot analysis of FLAG, RAD21 and CTCF upon FLAG-MAZ immunoprecipitation from nuclear extract of 293FT cells (*n* = 2 for FLAG and RAD21, *n* = 1 for CTCF). **b**, Venn diagram showing RAD21, CTCF, and MAZ binding in ESCs. ChIP-seq data are from one representative of two biological replicates for CTCF, RAD21, and MAZ. **c**, Venn diagram showing RAD21 binding in CTCF intact (untreated) versus CTCF degraded (auxin treatment, 48 h) ESCs, and the overlap of RAD21 binding with MAZ. ChIP-seq data are from one representative of two biological replicates. **d**, Top motif detected in RAD21 relocalized peaks (*n* = 11,862) in CTCF degraded (auxin, 48 h) ESCs by using MEME tools matches to the MAZ motif based on motif comparison via Tomtom (Extended Data Fig. 5b,c). The number of RAD21 sites (*n* = 9,143) where a de novo motif was detected is shown below the motif. **e**, ChIP-seq for CTCF and RAD21 reanalyzed in CTCF intact (untreated) and CTCF degraded (auxin treated, 48 h) ESCs at the indicated region in comparison to MAZ. ChIP-seq for MAZ is shown in WT ESCs. ChIP-seq tracks are from one representative of two biological replicates. Source data

We observed that similar to CTCF^12^, MAZ coimmunoprecipitates with the cohesin component RAD21 (Fig. 5a and Extended Data Fig. 2f,g), as demonstrated recently by Xiao et al^35^. CTCF, MAZ and RAD21 appear to colocalize at ~1,500 binding sites in ESCs (Fig. 5b), as described previously^35^. As cohesin was reported to be redistributed away from CTCF sites in the absence of CTCF^47^ (Fig. 5c and Supplementary Note 8) supporting the loop-extrusion model^48,49^, we explored the underlying DNA motifs in these regions of cohesin relocalization (Fig. 5c). Interestingly, the most enriched motif in the majority of relocalized RAD21 binding sites upon CTCF degradation resembled the MAZ binding motif (Fig. 5d and Extended Data Fig. 5b,c). Moreover, such redistributed RAD21 binding sites colocalized with MAZ binding in ESCs (Fig. 5c–e). Thus, our analyses suggest that RAD21 relocalizes to MAZ binding sites in the absence of CTCF in ESCs, implying a possible barrier function for MAZ.

### Skeletal pattern defects upon MAZ motif deletion at *HoxA* cluster

**Fig. 6:**
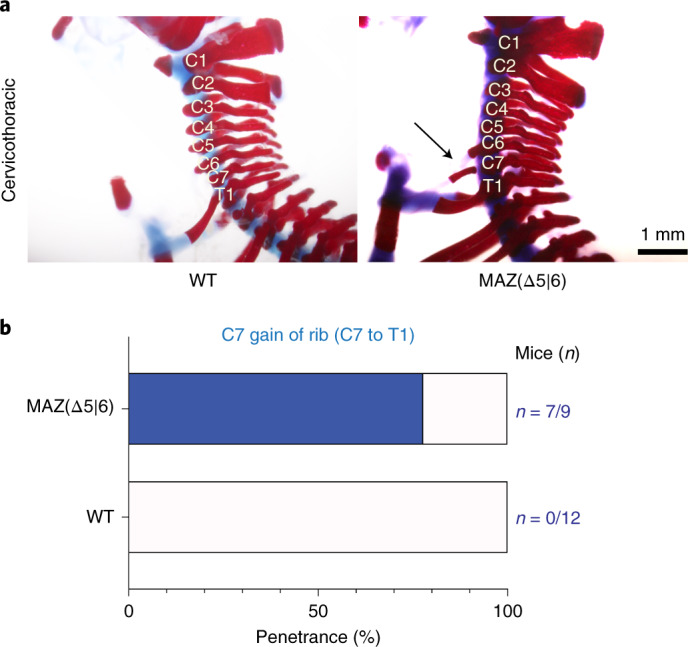
MAZ-delimited chromatin boundary at the *HoxA* cluster corresponds to boundaries in skeletal patterning in vivo. **a**, Representative Alcian blue–Alizarin red staining of axial skeletons indicating homeotic transformation (C7-to-T1 transformation) in WT versus MAZ *Hoxa5*|*6* mice at postnatal day 0.5. **b**, Bar plot demonstrating the percentage of pups (postnatal day 0.5) with the cervicothoracic transformation phenotype in MAZ *Hoxa5*|*6* compared to WT. Raw numbers of mice are shown in blue (Supplementary Fig. 6 for genetic deletions).

Our findings point to MAZ being critical for the proper establishment of positional identity and topological organization in ESC-derived cervical MNs. Thus, we hypothesized that MAZ motif deletions would produce homeotic transformations in vivo, similar to that shown for CTCF^16,17^. We generated embryos with MAZ *Hoxa5*|*6* motif deletions that ranged from 20 to 64 bp in *cis* to the MAZ motif using CRISPR (Supplementary Fig. 6) and investigated their skeletal development. In WT mice, there are 7 cervical (C1–C7), 13 thoracic (T1–T13), 6 lumbar (L1–L6) and 4 sacral (S1–S4) vertebrae^50^. Compared to WT mice, MAZ (Δ5|6) mouse embryos showed cervicothoracic C7-to-T1 transformation (Fig. 6a), similar to the homeotic transformations reported previously in the case of CTCF binding site deletions at the *Hox* clusters^17^. The observed phenotype indicates different levels of expressivity, mostly unilateral extension and ~78% penetrance (Fig. 6b). Thus, MAZ functions as a boundary factor in the *HoxA* cluster during development of the axial skeleton.

## Discussion

In this study, we demonstrated that an unbiased genome-wide CRISPR screen coupled with biochemical approaches enabled the identification of factors that function similarly to and in conjunction with CTCF. Our results place MAZ as a boundary factor that functions in partitioning *Hox* clusters into insulated domains wherein Trithorax and Polycomb activities are important in maintaining the distinct *Hox* gene expression patterns critical to anterior–posterior positioning during development. MAZ KO or MAZ binding site deletions at active and repressed gene borders in *Hox* clusters phenocopy the effect of CTCF binding site deletions at *Hox* clusters^16,17^. In particular, the transcriptional effect of MAZ motif deletions in *Hox* clusters points to their requirement for transcriptional insulation. This scenario may constitute a precedent in which DNA neighboring a CTCF site can influence boundary activity^51^, not only by indicating the requirement for a distinct DNA motif but also by revealing an insulation factor, MAZ, at *Hox* clusters.

**Fig. 7:**
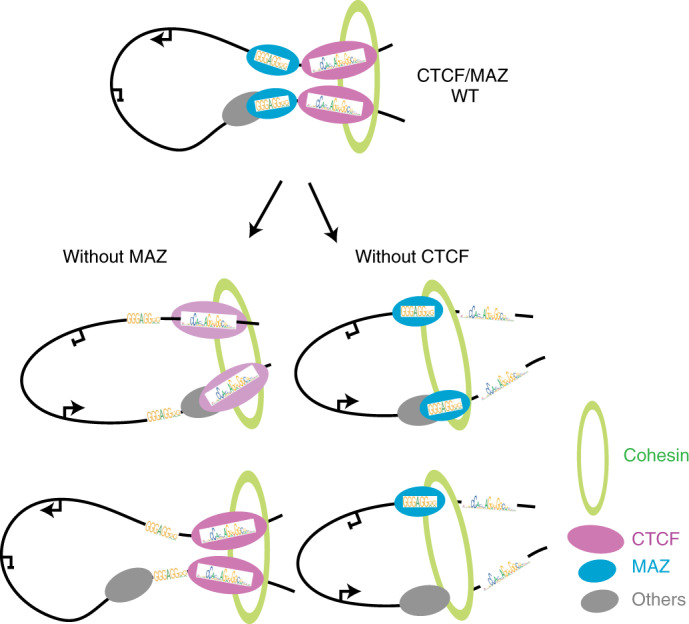
Model of the role of MAZ and CTCF in genome structure and function. Proposed model for the effect of MAZ loss (left) and CTCF depletion (right) on loops, as well as the existing loop-extrusion model of CTCF and cohesin^53,54^. MAZ interacts with RAD21 and localizes to loop anchors similar to CTCF (top). In the absence of MAZ, some looping interactions are impacted, accompanied by decreased CTCF occupancy (bottom left). In the absence of CTCF, TADs and more looping interactions are disrupted^44^ (bottom right). Based on the regulatory interactions impacted and the underlying chromatin context, such topological changes can be accompanied by alterations in gene expression as evident at the identified *Hox* clusters. The relative orientations of MAZ and CTCF motifs are depicted based on analyses of their presence at loop anchors, as shown in Extended Data Fig. 10.

In addition to CTCF and cohesin, MAZ also contributes to the integrity of TADs and contacts within TADs, as recently reported in K562 cells^35^. Looping interactions are impacted upon loss of MAZ, although the effects are not as large scale as the loss of essential architectural proteins such as CTCF^42^ or cohesin^52^ (Supplementary Note 9). Based on our current model, MAZ binding adjacent to CTCF and interaction of each with cohesin support their function at loops, possibly with other proteins (discussed below), such that disruption of these loops is accompanied by altered gene expression (Fig. 7). Moreover, although our results suggest that in the absence of CTCF, MAZ might serve as a possible block to cohesin during loop extrusion, possibly with other factors (Fig. 7, right), this model remains to be tested (Supplementary Note 9).

Consistent with our findings, *Maz*^−/−^ mice show perinatal lethality and developmental defects in the kidney and urinary track^53^ and eye development^54^, although other phenotypes remain to be investigated (Supplementary Note 10). Deletion of a critical CTCF site separating chromatin domains resulted in *Hoxd13* misexpression in the developing kidneys^55^. The cervicothoracic transformation we observed in the context of axial-skeleton development in mice with a MAZ motif deletion at *Hoxa5*|*6* is similar to that observed for a CTCF motif deletion at the *Hoxc5*|*6* region^17^. Although the transformation phenotype of the CTCF *Hoxc5*|*6* mice has been shown to be fully penetrant, MAZ *Hoxa5*|*6* motif deleted mice show similar penetrance levels to CTCF *Hoxa5*|*6:7*|*9* motif deletions^17^. Our findings are in agreement with those obtained in loss-of-function studies for *Hoxa5* and *Hoxa6* exhibiting a similar ectopic rib at C7^50,56,57^ and others for *Hoxc5* and *Hoxc6* showing cervicothoracic transformations^50,58,59^. Indeed, our observation of homeotic transformations in skeleton with the MAZ motif deletion at *Hoxa5*|*6* reinforces the importance of MAZ during normal development.

Our findings point to MAZ functioning as an insulator-like factor at *Hox* clusters in vitro and in vivo, sharing other properties with CTCF such as cohesin interaction and being critical to global gene regulation and genome organization (Supplementary Note 11). Such regulation is critical for the spatial and temporal progression of gene expression to ensure proper development. We note that this report has identified other candidates that may be required for the integrity of the CTCF boundary at the *HoxA* cluster as well as chromatin-based CTCF partners or colocalizing proteins under endogenous conditions during differentiation. These candidates were systematically narrowed down based on their insulation function at the *HoxA* cluster. Although our CRISPR loss-of-function screens are limited to the identification of those genes that are mainly nonessential, our biochemical approaches identified both essential and nonessential CTCF partners in undifferentiated versus differentiated cells. Similar to MAZ, some of these other candidates could potentially contribute to CTCF, cohesin and/or MAZ function, reflecting their impact on gene regulation during development.

## Methods

This study was performed under compliance with ethical regulations and approved by New York University (NYU)/NYU Grossman School of Medicine’s Institutional Biosafety Committee.

### Cell culture and MN differentiation

E14TG2a ﻿mouse ESCs (ES-E14TG2a,﻿ ATCC, CRL-1821) were cultured in standard medium supplemented with LIF and 2i conditions (1 mM MEK1/2 inhibitor (PD0325901, Stemgent) and 3 mM GSK3 inhibitor (CHIR99021, Stemgent)). For MN differentiation, a previously described protocol was applied^16^. Briefly, ESCs were differentiated into embryoid bodies in 2 days, and further patterning was induced with addition of 1 μM all-*trans*-retinoic acid (Sigma) and 0.5 μM smoothened agonist (Calbiochem). Biological replicates stand for independent differentiation experiments performed. 293FT cells (R70007, Thermo Fisher Scientific) were cultured in standard medium as described in the manufacturer’s protocol.

### CRISPR genome editing

sgRNAs were designed using CRISPR design tools (http://crispr.mit.edu/; currently available at https://benchling.com). All sgRNAs were cloned into SpCas9-2AGFP vector (Addgene: PX458) or into a lentiviral vector lentiGuide-puro (52963, Addgene). The sgRNAs were transfected into ESCs using Lipofectamine 2000 (Invitrogen) as described previously^16^ or infected into an ESC clone expressing lentiCas9-blast (52962, Addgene). In the case of CRISPR knockin cell lines, donor DNA (1 μl of 10 μM single-stranded DNA oligonucleotide or 3 μg pBluescriptSK (+) plasmid containing donor DNA) were transfected with 1 μg px458-sgRNAs. Single clones from GFP-positive FACS-sorted cells or puromycin (InvivoGen)-resistant cells were genotyped and confirmed by sequencing. When necessary, PCR products were further assessed by TOPO cloning (Invitrogen) and sequencing to distinguish the amplified products of different alleles. The sequencing chromatograms were aligned in Benchling. All sgRNAs, donors and genotyping primers are shown in Supplementary Table 1.

### Cell line generation for *Hoxa5:a7* dual reporter in WT and CTCF (Δ5|6:6|7) backgrounds

To generate *Hoxa5*:*a7* dual-reporter cells, ESCs were sequentially targeted at *Hoxa5* and *Hoxa7* loci, respectively. ESCs were initially transfected with sgRNA and donor pBluescriptSK (+) plasmid for *Hoxa5-P2A-mCherry* cell line generation using Lipofectamine (Invitrogen). *Hoxa5-mCherry* cell line was confirmed through genotyping, sequencing, and FACS analysis upon MN differentiation for the homozygous insertion of reporter. Next, the *Hoxa5-mCherry* cell line was transfected with sgRNA and donor pBluescriptSK (+) plasmid for generation of the dual *Hoxa5:a7* knock-in cell line, which was confirmed by genotyping, sequencing, and FACS analysis for the homozygous insertion of reporter. To demonstrate *Hoxa7-P2A-eGFP* expression in MNs, CTCF binding sites at *Hoxa5*|*6* and *Hoxa6*|*7* were removed via sequential CRISPR genome editing using respective sgRNAs, generating CTCF (Δ5|6) and CTCF (Δ5|6:6|7) deletion lines in the *Hoxa5:a7* dual-reporter background. For CRISPR library screen experiments, WT or CTCF (Δ5|6:6|7) dual-reporter lines were transduced with lentiCas9-blast (Addgene, 52962), and *Cas9*-expressing clones were obtained after selection with blasticidin (InvivoGen).

### Cell line generation for FLAG-CTCF-tagged cells

To generate the CTCF C-terminal FLAG-tagged cell line, E14TG2a mouse ESCs were targeted with sgRNA in SpCas9-2AGFP vector (Addgene, PX458) and single-stranded donor oligonucleotide at the *Ctcf* locus. The cell line was confirmed by genotyping, sequencing, and western blot for FLAG-CTCF.

### Cell line generation for MAZ KO cells

WT or CTCF (Δ5|6:6|7) *Hoxa5:a7* dual-reporter cells expressing *Cas9* were targeted with sgRNAs in lentiGuide-puro vector for *Maz*. Knock-out of *Maz* was confirmed by genotyping, sequencing, and western blot.

### Cell line generation for MAZ binding site deletions

*Hoxa5:a7* dual-reporter cells were targeted with sgRNAs in SpCas9-2AGFP vector (Addgene, PX458) for MAZ binding sites at *HoxA, HoxD* or *HoxC* clusters. Specific MAZ binding site deletions were confirmed by genotyping and sequencing.

### CRISPR screens

CRISPR genome-wide screens were done using methods described previously^22,23^. Briefly, GeCKO genome-wide pooled CRISPR libraries (Addgene, 1000000053) were amplified and deep-sequenced to confirm sgRNA representations, as shown previously^22^. A *Cas9*-expressing *Hoxa5:a7* ESC clone was transduced with the pooled lentiviral sgRNAs at a low multiplicity of infection (~0.4). The reporter ESCs were selected with puromycin, cultured for 7 days, differentiated into MNs in 6 days and sorted by FACS into two MN populations: (1) WT MNs (*mCherry*-positive/*eGFP-*negative cells) and (2) CTCF-boundary-disrupted MNs (double-positive cells). During the screens, 300× and 1,000× coverage was applied for genome-wide screens and secondary screens, respectively. CRISPR libraries were prepared at each time point and/or sorted population, and the relative sgRNA representation was assessed using next-generation sequencing, as described previously^22,23^.

### Custom library construction for secondary CRISPR screens

sgRNAs for custom library used in the secondary CRISPR screens were retrieved from a previously designed genome-wide mouse CRISPR KO pooled library (Brie)^60^. When required for several genes, sgRNAs were designed by using the Broad Institute CRISPRko gRNA design tools (https://portals.broadinstitute.org/gpp/public/analysis-tools/sgrna-design). All sgRNAs in the custom library in Supplementary Dataset 6 were synthesized as a pool by Twist Bioscience. The custom library was cloned into lentiGuide-puro vector, amplified and verified in terms of representation of all constructs using methods described previously^61^.

### Flow cytometry

Cells were trypsinized, filtered and stained with 4,6-diamidino-2-phenylindole (Sigma) to eliminate dead cells during analysis of *Hoxa5:a7* reporters in ESCs and MNs. *Hoxa5:a7* dual fluorescent reporter cells in WT versus other backgrounds were assessed by using single-color fluorescent reporters as controls in the same cell type as analyzed (i.e., MNs). *Hb9-T2A-eGFP* reporter cells (not shown) were used as GFP control in MNs (Supplementary Fig. 7a). For cell cycle analysis, ESCs were fixed in 75% ethanol, and DNA was stained with propidium iodide (Thermo Fisher Scientific) after RNase A (Thermo Fisher Scientific) treatment. FlowJo (version 8.7) was used for all FACS analysis (Supplementary Fig. 7b).

### Expression analysis

RNA was purified from cells with RNAeasy Plus Mini kit (Qiagen), and RT was performed on 1 μg RNA by using Superscript III (Life Technologies) and random hexamers (Thermo Fisher Scientific). RT-qPCRs were performed in replicates on 100 ng cDNA using PowerUp SYBR Green Master Mix (Thermo Fisher Scientific). The primers are listed in Supplementary Table 2. For RNA-seq analysis, 1 μg RNA was used to prepare ribo-minus RNA-seq libraries according to the manufacturer’s protocols by the NYU Genome Technology Center.

### ChIP-seq

ChIP-seq experiments were performed as described previously^62^ (see details regarding ESC fixation in Oksuz et al.^62^ and MN fixation in Narendra et al.^16^). Briefly, cells were fixed with 1% formaldehyde, nuclei were isolated and chromatin was fragmented to ~250 bp using a Diagenode Bioruptor. ChIP was performed using antibodies listed in Supplementary Table 2. Chromatin from *Drosophila* (1:100 ratio to ESC- or MN-derived chromatin), and *Drosophila*-specific H2Av antibody was used as a spike-in control in each sample. For ChIP-seq, libraries were prepared as described previously^16^ using 1–30 ng immunoprecipitated DNA. ChIP-qPCRs were performed with PowerUp SYBR Green Master Mix (Thermo Fisher Scientific) and detected by the Stratagene Mx3005p or QuantStudio 5 (Thermo Fisher Scientific) instrument. All ChIP-qPCR primers are listed in Supplementary Table 2.

### CUT&RUN

This method was performed as described previously^63,64^ using 100 000–200 000 cells that were sorted for double-positive (*Hoxa5-P2A-mCherry* and *Hoxa7-P2A-eGFP*) populations. WT MNs were treated similarly and collected through FACS. The cells were re-counted after sorting and the published protocol^65^ detailed in https://www.protocols.io by Janssens and Henikoff was followed. CUT&RUN experiments were analyzed with the methods described for ChIP-seq below.

### Preparation of 4C template

Cells were processed for 4C sequencing (4C-seq) as described previously^16,66^. Cells were trypsinized and counted, and 1 × 10^7^ cells were crosslinked with the crosslinking solution (2% formaldehyde and 10% FBS in 1× PBS) for 10 min at room temperature. After the reaction was quenched with glycine, cells were lysed on ice with 1 ml lysis buffer (50 mM Tris, pH 7.3, 150 mM NaCl, 5 mM EDTA, 0.5% NP-40 and 1% Triton X-100) for 15 min. Nuclei were spun down and frozen at −80 °C. Upon thawing on ice, nuclei were resuspended in 360 µl H_2_O. 60 µl of 10× *Dpn*II restriction buffer and 15 µl 10% SDS were added to the samples and left to shake for 1 h at 37 °C. Afterwards, 150 µl of 10% Triton X-100 was then added, and the samples were incubated for 1 h at 37 °C. After taking 5 µl undigested control, the remaining nuclei were incubated overnight with 200 U *Dpn*II restriction enzyme (New England Biolabs, R0543M). Then, 200 U fresh *Dpn*II was additionally added the next morning for 6 h. After digestion, *Dpn*II was inactivated with 80 µl 10% SDS, and a proximity ligation reaction was performed in a 7-ml volume using 4,000 U T4 DNA Ligase (Roche, M0202M). Then, 300 µg Proteinase K was added, and the crosslinks were reversed at 65^o^C overnight. Samples were treated with 300 µg RNase A for 45 min at 37 °C the next day, and DNA was precipitated with ethanol. A second restriction digestion was performed with 50 U Csp6l (Fermentas, ER0211) in 500 µl reaction volume. The enzyme was then inactivated at 65 °C for 25 min, and a proximity ligation reaction was done in 14-ml volume with 6,000 U T4 DNA ligase. Finally, the resulting DNA was precipitated with ethanol and purified using the QIAquick PCR purification kit.

### Preparation of Hi-C samples

Cells were removed, and 1 M cells were fixed in 2% formaldehyde (Fisher Chemical) according to the ARIMA-Hi-C protocol. Samples were prepared and sequenced according to the manufacturer’s protocol by the NYU Grossman School of Medicine’s Genome Technology Center.

### Cellular fractionation, immunoprecipitation and recombinant protein purification

All cellular fractionation and immunoprecipitation experiments were performed at 4 °C or on ice with buffers containing 1 μg ml^−1^ pepstatin, 1 μg ml^−1^ aprotonin, 1 μg ml^−1^ leupeptin, 0.3 mM PMSF, 10 mM sodium fluoride and 5 mM sodium orthovanadate. For FLAG affinity purification from native chromatin (native ChIP-mass spectrometry), nuclear extracts from ESCs and MNs were prepared using Buffer A and Buffer C, as described^67^. Cytosolic fraction was removed by buffer A (10 mM Tris, pH 7.9, 1.5 mM MgCl_2_, 10 mM KCl, and 0.5 mM dithiothreitol (DTT)). The pellet was resuspended in buffer C (20 mM Tris, pH 7.9, 25% glycerol, 420 mM NaCl, 1.5 mM MgCl_2_, 0.2 mM EDTA and 0.5 mM DTT) and incubated for 1 h to obtain nuclear extract. After removing the nuclear extract, the remaining nuclear pellet was solubilized by benzonase (Millipore) digestion in a buffer containing 50 mM Tris, pH 7.9, and 2 mM MgCl_2_. For FLAG affinity purification from native chromatin and MS, 20 mg nuclear pellet was incubated with 200 μl FLAG M2 beads in BC250 overnight and washed six times with BC250 containing 0.05% NP40, as described elsewhere^68^. Two elutions were performed with 0.5 mg ml^−1^ FLAG peptide in BC50 (without any protease inhibitors) with rotation at 4 °C for 2 h for a total of 4 h. The eluate was sent to the Biological Mass Spectrometry Facility of Robert Wood Johnson Medical School and Rutgers and analyzed by liquid chromatography tandem MS. Peptide counts are shown for the native ChIP-MS experiments in Supplementary Dataset 4.

For FLAG affinity purification from crosslinked chromatin (crosslinked ChIP-MS), a modified version of a previously reported protocol was applied^32,33^. Briefly, cells were crosslinked and sonicated as described above for ChIP-seq with the exception to obtain a larger fragment size that includes approximately three to five nucleosomes. Then, 3 mg chromatin was used for FLAG affinity purification, and FLAG elutions were performed after stringent washes as described previously^32^, but excluding the second step in the protocol wherein DNA is biotinylated. After decrosslinking, samples were sent to the Biological Mass Spectrometry Facility of Robert Wood Johnson Medical School and Rutgers and analyzed by liquid chromatography tandem MS.

For extraction in 293FT cells, CβF expression vectors containing cDNAs for CTCF (mouse) or MAZ (mouse) were transfected into 293FT cells using polyethylenimine (PEI), and nuclei was prepared using TMSD and BA450 buffers, as described previously^69,70^. Briefly, TMSD buffer (20 mM HEPES, 5 mM MgCl_2_, 85.5 g l^−1^ sucrose, 25 mM NaCl and 1 mM DTT) was used for cytosol removal, and nuclear extraction was done in BA450 buffer (20 mM HEPES, 450 mM NaCl, 5% glycerol and 0.2 mM EDTA). FLAG affinity purification and FLAG peptide elution were performed similarly in the nuclear fraction.

The FLAG-MAZ recombinant protein used in electrophoretic mobility shift assays (EMSA) was purified from 293FT cells expressing CβF expression vectors containing cDNA for MAZ as described before^69,70^. The nuclear extraction was performed as detailed above with TMSD buffer followed by BA450 buffer. FLAG affinity purification was performed under the wash conditions with BA450. FLAG peptide elution was done to elute FLAG-MAZ. The purity of FLAG-MAZ was ensured by Coomassie blue staining (~ %95 purity).

### Library construction

All libraries were prepared according to the manufacturer’s instructions (Illumina). CRISPR libraries were prepared by performing two-step PCRs as described elsewhere^23^. Briefly, sgRNAs were amplified from genomic DNA by keeping the coverage maintained throughout the screens (300× for the GeCKO v2 library and 1,000× for the custom library in secondary screens) and performing secondary amplifications using Phusion polymerase (New England Biolabs) to attach Illumina adaptors (Supplementary Table 3). ChIP-seq libraries were prepared as described previously^16^. RNA-seq libraries were prepared using KAPA library preparation kits. Libraries for 4C-seq were constructed by attaching barcoded Illumina adapters to the 5ʹ end of the primer as described previously^16^ (Supplementary Table 6). PCR reactions were performed using the Expand Long Template PCR System (Roche), and approximately 100–700 bp DNA was gel purified and quantified before sequencing. Hi-C libraries were prepared according to the ARIMA standard Hi-C protocols by the NYU Grossman School of Medicine’s Genome Technology Center.

### Electrophoretic mobility shift assays

Single-stranded oligonucleotides with MAZ DNA binding sites from the mouse *HoxA* and *HoxD* loci were annealed and radioisotope-labeled using 400 pmol double-stranded DNA, T4 PNK (Thermo Fisher Scientific, EK0031) and [γP^32^]-ATP (Supplementary Table 5). The probes were purified by G-25 columns (GE Healthcare, 27532501). After the labeling reaction, 40 pM probe was resuspended in binding buffer (25 mM HEPES, 50 mM NaCl, 5% glycerol, 5 mM MgCl_2_, 1 mM ZnSO_4_ and 2 μg salmon sperm DNA). The reactions were then incubated with increasing amounts of mouse recombinant MAZ (0.25, 0.5 and 0.75 μg) for 4 h at 30 °C. After the incubation, the reactions were run on 5% acrylamide gels for 30 min at room temperature, 200 V and 0.25× TAE buffer. Finally, the acrylamide gel was dried and exposed overnight.

### CRISPR zygotic injection

MAZ *Hoxa5*|*6* mutant mice were generated by zygotic injection^71^ as described previously^17^. Briefly, 50 ng µl^−1^ gRNA template (Synthego) and 100 ng µl^−1^ *Cas9* mRNA were injected into the cytoplasm of ~150 C57BL/6 zygotes in the NYU Grossman School of Medicine’s Rodent Genetic Engineering Laboratory. Surviving embryos were transferred to four pseudopregnant females, and a total of 27 pups were born. These pups were genotyped by PCR using genotyping primers (Supplementary Table 1) and Sanger sequencing, indicating the genomic alterations as summarized in Supplementary Figure 6. When required, TOPO cloning was applied to reveal different alleles by Sanger sequencing (Supplementary Fig. 6). Mouse studies were approved by NYU Grossman School of Medicine’s Institutional Animal Care and Use Committee. Housing conditions were as follows: dark/light cycle, 6:30 pm to 6:30 am (off) / 6:30 am to 6:30 pm (on); temperature, 21 °C ± 1 or 2 °C; and humidity range, 30–70%.

### Alcian blue–Alizarin red staining of skeleton

The neonates (postnatal day 0.5) were dissected by removing the skin and organs, and skeletal staining was performed as described previously^17^. Embryos were fixed for 4 days in 95% ethanol with rocking at room temperature. Ethanol was replaced with Alcian blue stain (0.03% Alcian blue, 80% ethanol and 20% acetic acid) for 24 h with rocking at room temperature. Embryos were washed with 95% ethanol twice for 1 h each time with rocking at room temperature and transferred to 2% KOH solution for 24 h. The specimens were then stained with Alizarin red solution (0.03% Alizarin red and 1% KOH in water) for 24 h. Finally, the skeleton was further washed in 1% KOH/20% glycerol for 6 days, 1% KOH/50% glycerol for 10 days and passed to 100% glycerol. In case of long-term storage, the skeletons were transferred to 4:1 glycerol/ethanol.

### Data analysis of CRISPR screens

MAGeCK tools (version 0.5.7) was used for all primary and secondary CRISPR screen analyses^27,28^. Genome-wide screens with GeCKO v2 library A (three sgRNAs per gene) and GeCKO v2 library B (three sgRNAs per gene) were analyzed together in total populations of ESCs and MNs to identify essential/differentiation-related genes (negative selections). The analysis was done separately for library A (two screens) and library B (two screens) in sorted MN populations to identify genes affecting CTCF-boundary function (positive selection). When there is no replicate in a CRISPR screen, MAGeCK estimates the mean and variance of all samples from both control and treated conditions, assuming that most sgRNAs have no effect on selection^27^. The PANTHER database was used for GO analysis^72^, and the PANTHER overexpression test tool was utilized for statistical analysis^73^. To generate Venn diagrams in CRISPR screens, web tools (http://genevenn.sourceforge.net) were used.

### Data analysis of RNA-seq

RNA-seq data were analyzed as described previously^16^. Briefly, sequence reads were mapped to mm10 reference genome with bowtie2 (version 2.3.4.1) (ref. ^74^), and normalized differential gene expression was obtained with DESeq2 (version 1.26.0) (refs. ^75,76^). Differential gene expression analysis was performed using the Wald test built into DESeq2 with an FDR cutoff of 0.05. Relevant expression and *P* values are listed for differentially expressed genes in Supplementary Datasets 10, 11, 13 and 14. The PANTHER database was used for GO analysis^72^.

### Data analysis of ChIP-seq

ChIP-seq experiments were analyzed as described previously^62^. In brief, sequence reads were mapped to mm10 reference genome with bowtie2 (version 2.3.4.1) using default parameters^74^. Quality filtering and removal of PCR duplicates were performed by using SAMtools (version 1.9) (ref. ^77^). After normalization with the spike-in *Drosophila* read counts, normalized ChIP-seq read densities were visualized in Integrative Genomics Viewer version 2.4.14 (ref. ^78^). MACS (version 1.4.2) was used for narrow peak calling using default parameters of macs2 (ref. ^79^). Heat maps were generated using deepTools in R (version 3.1.2) (ref. ^80^). The ChIPpeakAnno package (version 3.20.1) from Bioconductor^81^ was used to draw Venn diagrams to visualize the overlap among ChIP-seq samples. In addition, BEDTools (version 2.27.1) was also used for the assessment of overlaps^82^. The replicates were assessed similarly by visualizing at Integrative Genomics Viewer (version 2.4.14) and generating heat maps. ChIP-seq BED file coordinates were converted into fasta using fetch sequences tool within Regulatory Sequence Analysis Tools^83^; MEME (version 5.4.1) was used for motif analysis of MAZ in ESCs and MNs^84^, SpaMo (version 5.4.1) was used for distance analysis between CTCF and MAZ motifs in ESCs and MNs^85^ and Tomtom (version 5.4.1) was used as a motif comparison tool^86^. CTCF and MAZ occupancies in the subset of genes shown in Extended Data Fig. 5e were analyzed by using EaSeq software (version 1.111)^87^.

### Data analysis of 4C-seq

4C-seq data were analyzed using the 4C-ker (version 0.0.0.9000) pipeline^88^. Briefly, reads were mapped to mm10 reduced genome, and undigested and self-ligated fragments were removed. Near-bait analysis was generated in R by using 4C-ker tools.

### Data analysis of Hi-C

All samples were prepared in two biological replicates. All Hi-C data were analyzed by the Hi-C bench platform (version 0.1) (ref. ^89^). Throughout our comprehensive analysis, the following operations were done using Hi-C bench. Internally, bowtie2 (ref. ^90^) was used to align the paired reads using mm10 reference genome and only the read pairs uniquely mapped to the same chromosome with the mapping quality ≥20 and the pair distance ≥25 kb were used. Then, the interaction matrix was tabulated by reading the coordinates of aligned reads in 20-kb bins. To ensure that each interaction bin showed equal visibility, the iterative correction method^91^ was used to normalize the bins.

For the compartment analysis, the Hi-C interaction bins were divided into A and B compartments using the first principal component values from HOMER’s (version 4.11) runHiCpca^92,93^. Using Hi-C-bench, the compartment changes from comparison of two cell types for the bins in the interaction matrix were visualized by the stacked bar plot.

TADs were defined as shown before^89,94^ with the insulating window of 500 kb. The boundaries of TADs were called from the boundary score using the “ratio” method defined^89^, wherein each TAD boundary had a noticeably lower boundary score than the neighboring region. The score was calculated for each 20-kb bin using the window size of 250 kb, 500 kb and 1,000 kb. In the principal-component analysis to distinguish the differences, the boundary score for every replicate and cell type was combined, quantile normalized and plotted. Then, for each TAD, the magnitude of intra-TAD “activity” was defined as reported previously^94^. The cutoff for significantly differential TADs was Benjamini–Hochberg corrected *Q* value of 0.05 and no cutoff for the fold change.

Significantly enriched chromatin loops were called using FitHi-C (version 2.0.7) (ref. ^95^) with default parameters. To characterize the loops by CTCF and MAZ ChIP-seq levels, APA software^46^ was used to show the averaged profile. When filtering the Hi-C loops for the occupancy of CTCF and MAZ, a binary cutoff was placed such that the ChIP-seq signal at the anchors had values shown in Supplementary Table 4. The genome sequence that matched the transcription factor motifs of mouse CTCF and MAZ from the Catalog of Inferred Sequence Binding Preferences^96^ was found from PWMScan (version 1.1.9) (ref. ^97^). Visualization of Hi-C and associated ChIP-seq data were made with pyGenomeTracks (version 3.5) (ref. ^98^).

### Analysis of CTCF/MAZ motif orientation in Hi-C anchors

A chromatin loop found by Hi-C can have one or multiple motif hits of transcription factors such as CTCF or MAZ, in either the 5ʹ or 3ʹ anchors or both. The similarity of sequence between the loci and the known transcription factor motifs was calculated using the motifFinder feature of Juicer (version 1.5) (ref. ^99^), and the location and the direction of motif matches were produced. To reduce the complexity and the potential false positives, the sequences were compared only at the intersection of loop anchors and the ChIP-seq peaks for respective transcription factors. Find Individual Motif Occurrences of MEME suite (version 5.2.0) (refs. ^100,101^) was used with a *P* value cutoff of 10^−3^ to associate anchors with motifs. In case of multiple motif hits in the anchors, motifFinder found one with the highest score and reported it. One of the CTCF motifs was chosen from the M1 motif^102^ and downloaded from Juicer’s reference data. Also, the position-weight matrices of CTCF and MAZ motifs found by our study (Extended Data Fig. 5b,c) were used.

For the pairwise motif orientation from 5ʹ and 3ʹ anchors, only the cases wherein motifs were located in both anchors were considered. If a loop contained the motifs hits wherein its 5ʹ anchor harbored a positive direction and its 3ʹ anchor had a negative direction, the loop was defined as having a convergent motif hit. In case of the negative direction on 5ʹ and the positive direction on 3ʹ anchors, the loop was defined to contain a divergent motif hit. If the anchors contained all positive or all negative direction on both anchors, then the loop was defined as tandem. The proportion of convergent, tandem or divergent loops over the sum of loop groups was compared across experiment types.

### Statistical analysis

Statistical analyses related to experiments are described above in each section. Statistical analyses in bar plots were performed using GraphPad Prism (version 9.2.0). The R package pcr (ref. ^103^) was used in Extended Data Fig. 4e–g.

### Reporting Summary

Further information on research design is available in the Nature Research Reporting Summary linked to this article.

## Online content

Any methods, additional references, Nature Research reporting summaries, source data, extended data, supplementary information, acknowledgements, peer review information; details of author contributions and competing interests; and statements of data and code availability are available at 10.1038/s41588-021-01008-5.

## Supplementary information

Supplementary Information Supplementary Notes 1–11, Figures 1–7 (including figure legends), Tables 1–6, captions for Supplementary Datasets 1–14 and References

Reporting Summary

Supplementary Data 1 Excel workbook containing Supplementary Datasets 1–14.

## Data Availability

Sequencing data have been deposited at Gene Expression Omnibus (GSE157139). We used the publicly available datasets in Fig. 5b–e pertaining to CTCF-degron ESCs (GEO GSE98671 and GSE156868). The list of differentially expressed genes in CTCF-degron ESCs used in Fig. 2m was previously reported^42^. Proteomic data have been deposited to the ProteomeXchange Consortium via PRIDE (PXD030452 and PXD030543). Supplementary Datasets 1–14 are provided with this paper. Source data are provided with this paper.

## Source data

Source Data Fig. 5 Uncropped western blots.
Source Data Extended Data Fig. 1 Uncropped western blots.
Source Data Extended Data Fig. 2 Uncropped western blots.
Source Data Extended Data Fig. 3 Uncropped western blots.
Source Data Extended Data Fig. 6 Uncropped EMSA blots.
Source Data Extended Data Fig. 7 Uncropped EMSA blots.

## Code availability

Analysis tools used in this study have been published before as described in Methods and Reporting Summary.
