# Supplementary material for: CRISPR and biochemical screens identify MAZ as a cofactor in CTCF-mediated insulation at Hox clusters
Source: Nat Genet. 2022 Feb 10;54(2):202–12. doi: 10.1038/s41588-021-01008-5 (PMC8837555; doi:10.1038/s41588-021-01008-5)
Supplement: Supplementary file 1 — Supplementary Notes 1–11, Figures 1–7 (including figure legends), Tables 1–6, captions for Supplementary Datasets 1–14 and References [file 41588_2021_1008_MOESM1_ESM.pdf]

nature portfolio

https://doi.org/10.1038/s41588-021-01008-5

Supplementary information

CRISPR and biochemical screens identify MAZ as a cofactor in CTCF-mediated insulation at *Hox* clusters

In the format provided by the authors and unedited

Supplementary Materials for

CRISPR and biochemical screens identify MAZ as a cofactor in CTCF-mediated insulation at *Hox* Clusters

Havva Ortabozkoyun-Kara[1,2], Pin-Yao Huang[1,2], Hyunwoo Cho[3,4,5], Varun Narendra[6], Gary Leroy[1,2], Edgar Gonzalez-Buendia[1,2], Jane A. Skok[3], Aristotelis Tsirigos[3,4,7], Esteban O. Mazzoni[8] and Danny Reinberg[1,2,*]

Correspondence to: Danny.Reinberg@nyulangone.org

Supplementary Notes

Supplementary Note 1

The reduction in *Hoxa5-P2A-mCherry* upon de-repression of *Hoxa7-P2A-EGFP* observed in **Extended Data Figure 1c,d** is likely due to the phenomenon of posterior prevalence of *Hox* gene expression[1,2].

Supplementary Note 2

Throughout the screen, *Hox* gene reporter expression was not observed in ESCs upon perturbation of any gene, which increased our confidence in identifying CTCF-boundary related candidates rather than general repressors. Notably, the CRISPR loss-of-function screen setup in this differentiation system mainly allows for the identification of non-essential genes involved in CTCF-boundary function at the *HoxA* cluster due to depletion of essential genes/differentiation-related genes at earlier time-points.

Supplementary Note 3

Affinity purification was performed to enrich for CTCF-bound chromatin fragments using FLAG pull-down followed by FLAG peptide elution to minimize nonspecific interactions. Mass spectrometry (MS) was performed on eluted proteins. In the case of both ESCs and MNs, wild-type cells without the FLAG-tag served as background controls to normalize FLAG IPs.

Supplementary Note 4

This custom library included sgRNAs targeting the list of candidates from the primary screens, and other proteins that co-purified with CTCF (**Extended Data Fig. 2h**). Therefore, all candidates regardless of their essentiality were re-evaluated through these independent secondary screens.

The sgRNAs in the custom library were retrieved from another genome-wide library constructed with improved design tools[3].

The identification of several essential genes (*i.e. Ctcf,* cohesin components…*etc.*) in secondary CRISPR loss-of-function screens is likely due to heterozygous perturbation of these genes and the increased power of secondary screens with a smaller library.

Supplementary Note 5

Although MAZ and CTCF co-localize on the cross-linked chromatin (**Fig. 1g**, and **Fig. 2j-l**), they did not co-immunoprecipitate (co-IP) under native conditions (**Extended Data Fig. 2e-g**).

Supplementary Note 6

We reasoned that if MAZ binding close to a CTCF boundary has a direct effect on anterior-posterior patterning, deletion of its binding motif could alter *Hox* gene expression similar to MAZ KO in MNs. Importantly, the evidence generated by individual motif deletions of MAZ at *Hox* clusters eliminates the possibility that the MAZ KO phenotype on *Hox* was attributable to the dysfunction of other developmental genes or pathways (**Extended Data Fig. 4a-b**, also see **Fig. 2g** and **Fig. 3d**).

Supplementary Note 7

As expected, the active (A) and inactive (B) compartments were not affected upon MAZ KO in ESCs and MNs (**Extended Data Fig. 8b**). There were also changes in boundary scores upon MAZ KO in ESCs and MNs as shown by PCA (**Extended Data Fig. 8c**). Moreover, we observed that some loop domains in larger ranges are also slightly downregulated upon MAZ KO while we did not observe local effects of MAZ KO on *Hox* cluster organization with respect to the CTCF and MAZ boundary, within the resolution of HiC (**Supplementary Fig. 3** to **5**).

Supplementary Note 8

In previous studies, a subset of cohesin binding sites was described to co-localize with NIPBL, the cohesin loader protein, and mediator complex in mouse ESCs[4]. Indeed, 50% of the NIPBL binding sites contain the motif for SP1[5], another zinc finger protein binding to a GC-rich DNA motif 'GGGCGG' similar to MAZ[6]. In addition, transcription has been shown to re-locate cohesin to active promoters in the absence of CTCF[7].

Supplementary Note 9

In particular, MAZ appears to be present at the loop anchors with or without CTCF (**Fig. 4d**), possibly contributing to CTCF stability (**Extended Data Fig. 5f-i**), and both proteins interact with cohesin components independently (**Fig. 5a** and **Extended Data Fig. 2f-g**). These observations are consistent with a recent study showing changes to the global architecture upon MAZ knockdown in K562 cells[8], yet our work reveals the contribution of MAZ to the organization of the architectural genome during differentiation via KO studies.

Given that MAZ-containing loop anchors were detected based on HiC (**Fig. 4d** and **Extended Data Fig. 8g**), that MAZ and RAD21 interact (**Fig. 5a**), and that RAD21 re-localized to MAZ binding sites upon CTCF depletion (**Fig. 5c**), MAZ may function in concert with cohesin, either together with or independently of CTCF, which requires further testing (see **Fig. 7**). Interestingly, many short-range chromosomal contacts, including ones connecting active promoters with each other and with active enhancers are not affected by CTCF and cohesin depletion, suggesting the existence of alternative enhancer-promoter pairing mechanisms independent of these proteins[9]. We speculate that the contacts remaining after depleting the major architectural proteins such as CTCF and cohesin could correlate with MAZ and possibly other factors yet to be determined.

Supplementary Note 10

Although more than 25 out of 39 *Hox* genes are expressed in the kidney, the role of *Hox* genes other than the *Hox11* paralogous group seems to be under active investigation[10]. In addition, *HoxD* gene expression can be regulated through a putative mesenchyme enhancer (*Hoxd9* to *Hoxd12*) and ureteric bud enhancer (*Hoxd1* to *Hoxd9*) in kidney development[11]. Hence, it can be informative to study possible *Hox* gene misexpression patterns in the kidney or urinary track in *Maz* $^{-/-}$ mice.

Supplementary Note 11

Although gene expression changes have been correlated with alterations in topological organization upon loss of MAZ (**Fig. 4c**, **Extended Data Fig. 8f** and **Extended Data Fig. 9**), we emphasize that the effect will vary for different enhancer-promoter pairs, and as a function of other regulatory elements within their domain, including insulators (*i.e.* CTCF), partially redundant enhancers, and structural loop anchors[12]. We propose that both CTCF- and MAZ-mediated boundary activity are regulated in a cell-type specific manner in relation to the local chromatin environment. In ESCs, the insulation capacity of CTCF- and/or MAZ-containing loops is overridden by self-assembling Polycomb domains. As observed during differentiation, *Hox* clusters form active and repressed chromatin domains, allowing CTCF- and/or MAZ-mediated alteration of regulatory contacts.

Supplementary Figures

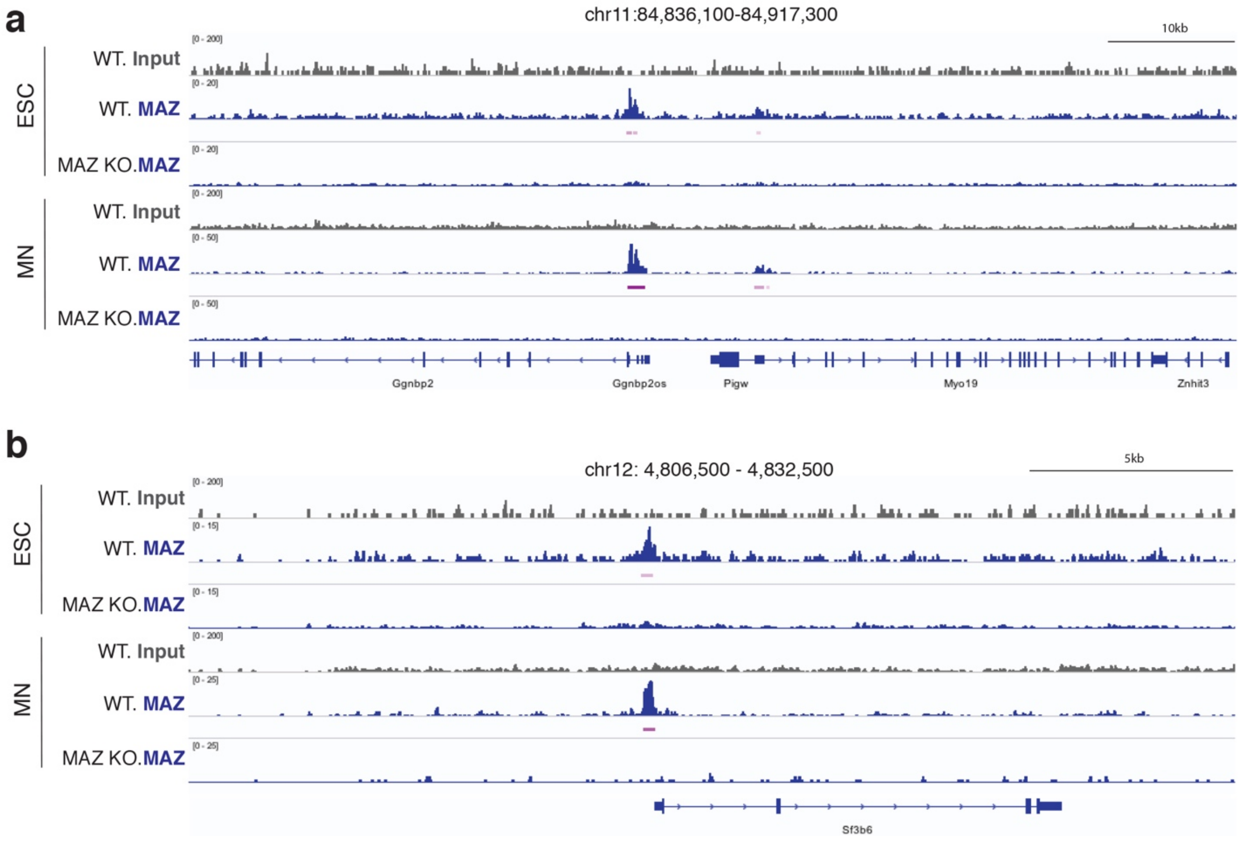

Supplementary Fig. 1 | MAZ ChIPseq in ESCs and MNs. a, MAZ ChIPseq peaks at *Ggnbp2os* gene locus and *Sf3b6* gene locus **(b)**. Purple is narrow peak files called by MACS2.

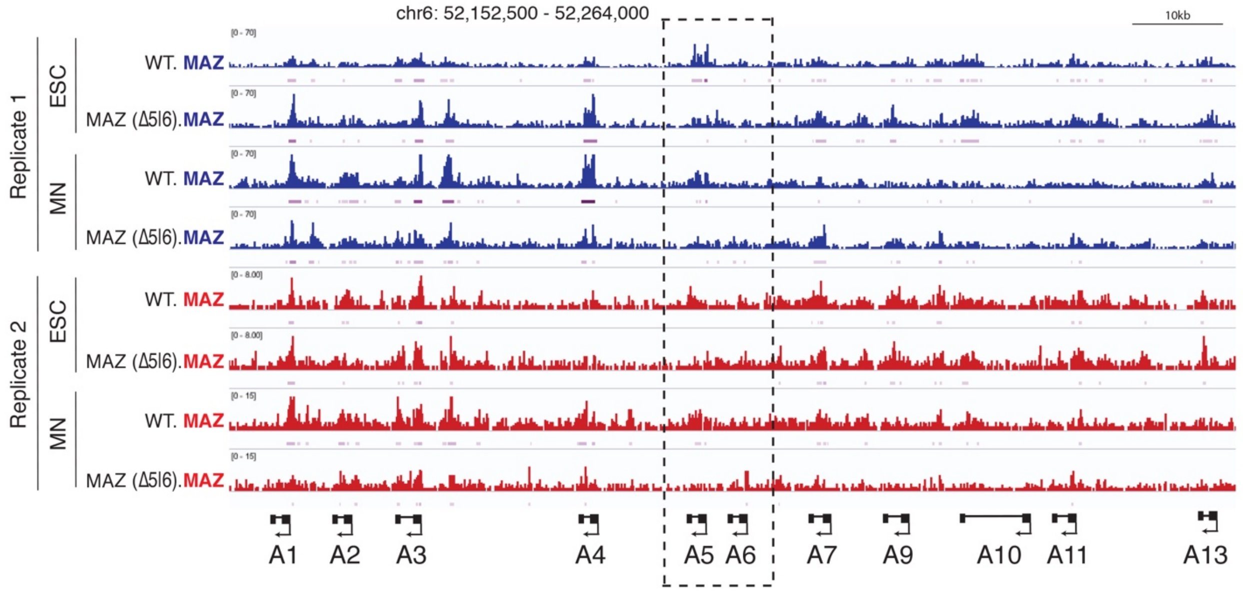

Supplementary Fig. 2 | Reduced MAZ ChIPseq signal at *Hoxa5|6* region upon MAZ motif deletion. MAZ ChIPseq signal in *HoxA* cluster in WT versus MAZ motif deletion in ESCs and MNs. Two biological replicates are indicated with blue and red colors. The peaks called by MACS2 are indicated under the ChIPseq tracks in purple.

131

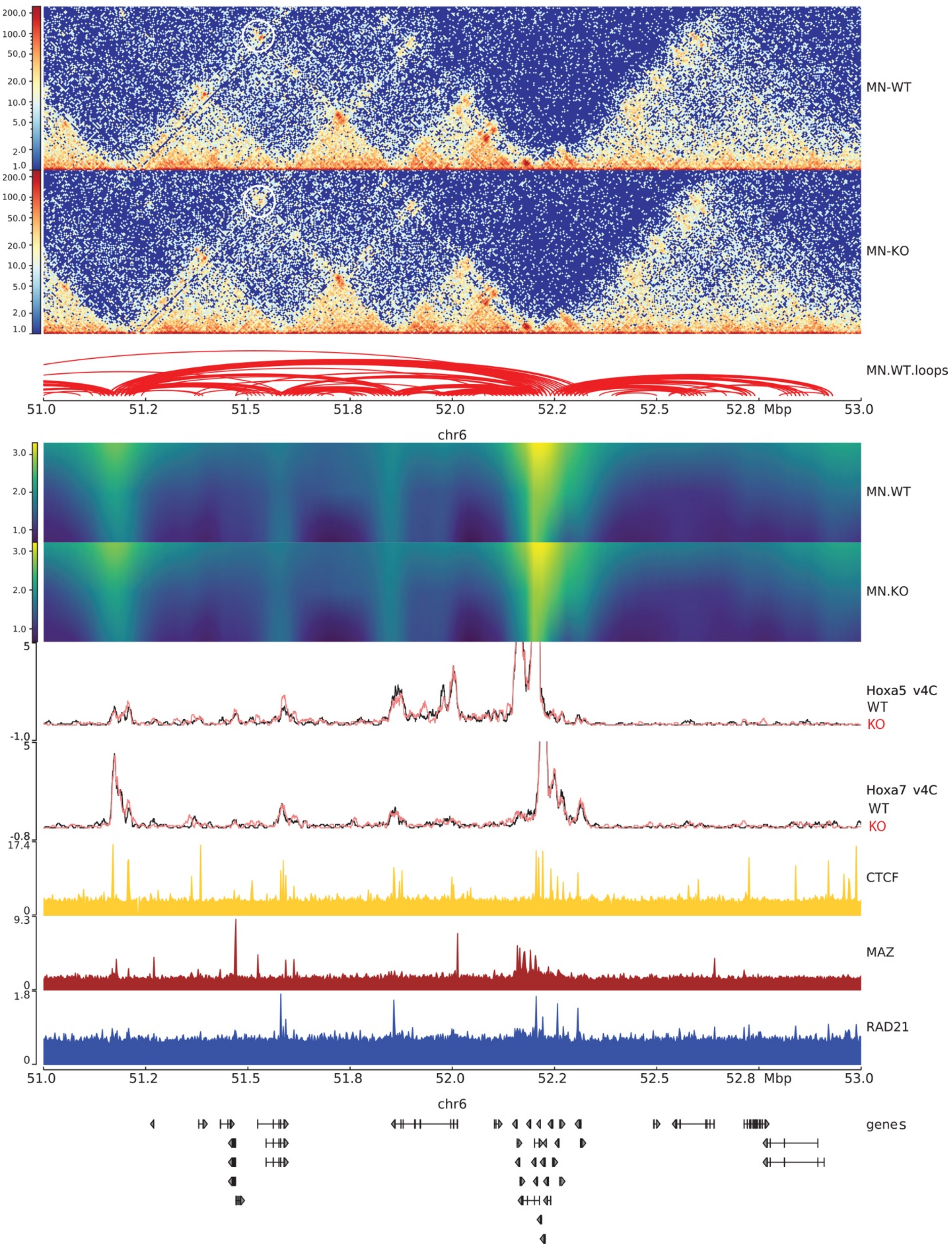

MN-WT
MN-KO
MN.WT.loops
chr6
MN.WT
MN.KO
Hoxa5 v4C
WT
KO
Hoxa7 v4C
WT
KO
CTCF
MAZ
RAD21
chr6
genes

Supplementary Fig. 3 | Visualization of HiC analysis at the indicated region in WT and MAZ KO MNs. Visualization of Hi-C contact matrices for a zoomed-in region on chromosome 6 with a change of loop activity in WT vs MAZ KO MNs. Shown below are insulation score heat maps in WT MNs and MAZ KO MNs, virtual 4C plots with *Hoxa5* as viewpoint and *Hoxa7* as viewpoint, and ChIP-seq read densities for CTCF, MAZ, and RAD21. The last track shows gene annotations.

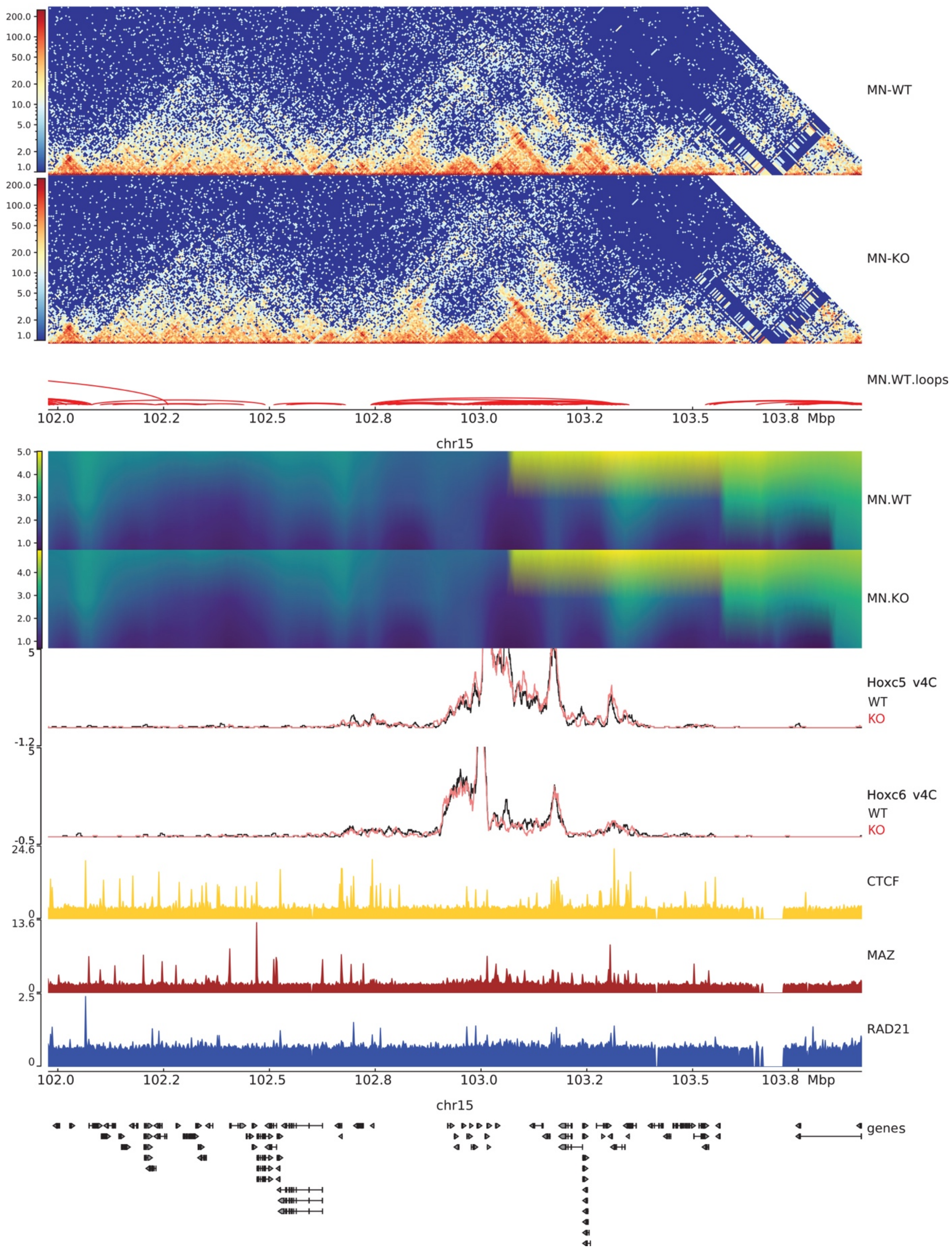

MN-WT
MN-KO
MN.WT.loops
102.0
102.2
102.5
102.8
103.0
103.2
103.5
103.8 Mbp
chr15
MN.WT
MN.KO
Hoxc5 v4C
WT
KO
Hoxc6 v4C
WT
KO
CTCF
MAZ
RAD21
genes

139

Supplementary Fig. 4 | Visualization of HiC analysis at indicated regions in WT and MAZ KO MNs. Visualization of Hi-C contact matrices for a zoomed-in region on chromosome 15 in WT vs MAZ KO MNs. Shown below are insulation score heat maps in WT MNs and MAZ KO MNs, virtual 4C plots with *Hoxc5* as viewpoint and *Hoxc6* as viewpoint, and ChIP-seq read densities for CTCF, MAZ, and RAD21. The last track shows gene annotations.

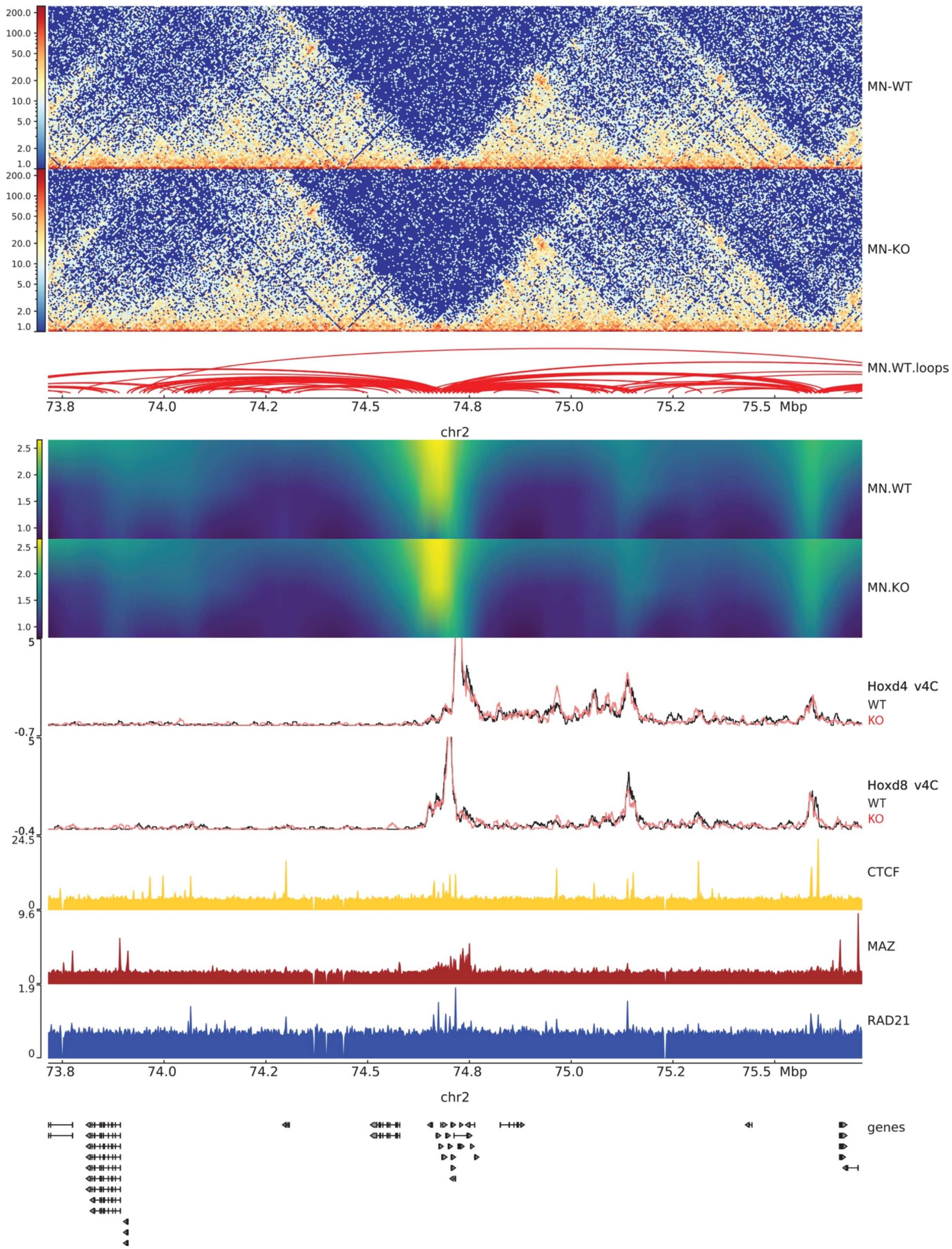
MN-WT
MN-KO
MN.WT.loops
73.8
74.0
74.2
74.5
74.8
75.0
75.2
75.5 Mbp
chr2
MN.WT
MN.KO
Hoxd4 v4C
WT
KO
Hoxd8 v4C
WT
KO
CTCF
MAZ
RAD21
genes

146

Supplementary Fig. 5 | Visualization of HiC analysis at indicated regions in WT and MAZ KO MNs. Visualization of Hi-C contact matrices for a zoomed-in region on chromosome 2 in WT vs MAZ KO MNs. Shown below are insulation score heat maps in WT MNs and MAZ KO MNs, virtual 4C plots with *Hoxd4* as viewpoint and *Hoxd8* as viewpoint, and ChIP-seq read densities for CTCF, MAZ, and RAD21. The last track shows gene annotations.

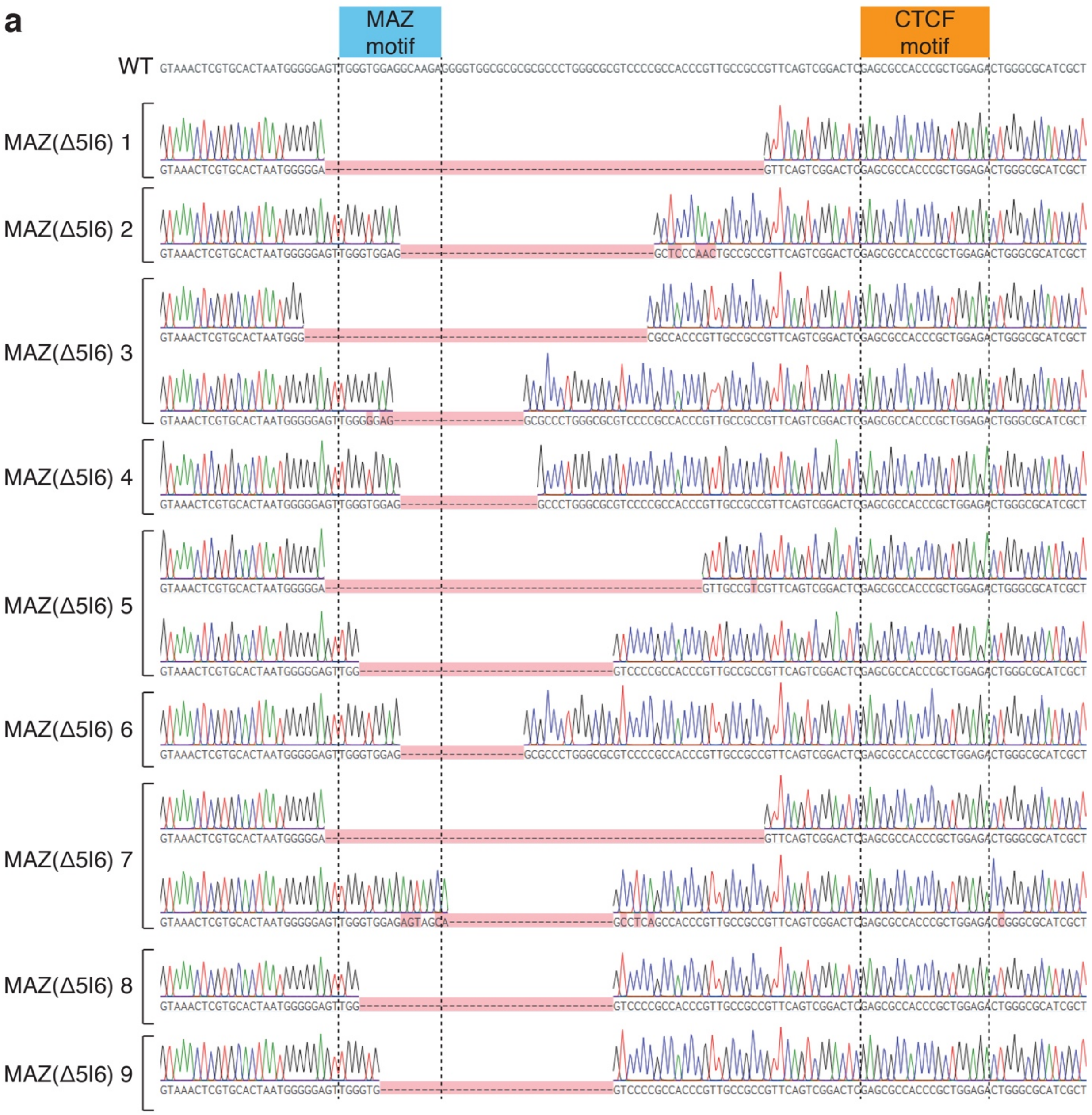

b

MAZ(Δ5|6) MICE	GENOTYPE	MAZ motif	CTCF motif
1	64 bp deletion	all motif deletion	intact
2	37 bp deletion	6 bp deletion	intact
3	50 bp deletion / 19 bp deletion & 3 bp mutation	all motif deletion/7bp deletion	intact
4	20 bp deletion	6 bp deletion	intact
5	37 bp deletion / 55 bp deletion	all motif deletion/12 bp deletion	intact
6	18 bp deletion	6 bp deletion	intact
7	64 bp deletion / 24 bp deletion	all motif deletion/4bp mutation	intact
8	37 bp deletion	12 bp deletion	intact
9	34 bp deletion	9 bp deletion	intact

Supplementary Fig. 6 | CRISPR-based zygotic injection results in deletions of a *HOXA* MAZ motif *in vivo*. a, CRISPR based deletions in 9 mice upon targeting of the MAZ motif region at *HoxA* cluster. MAZ motif and CTCF motifs are indicated. **b,** Table summarizing the length of CRISPR deletions in mice with respect to MAZ and CTCF motifs.

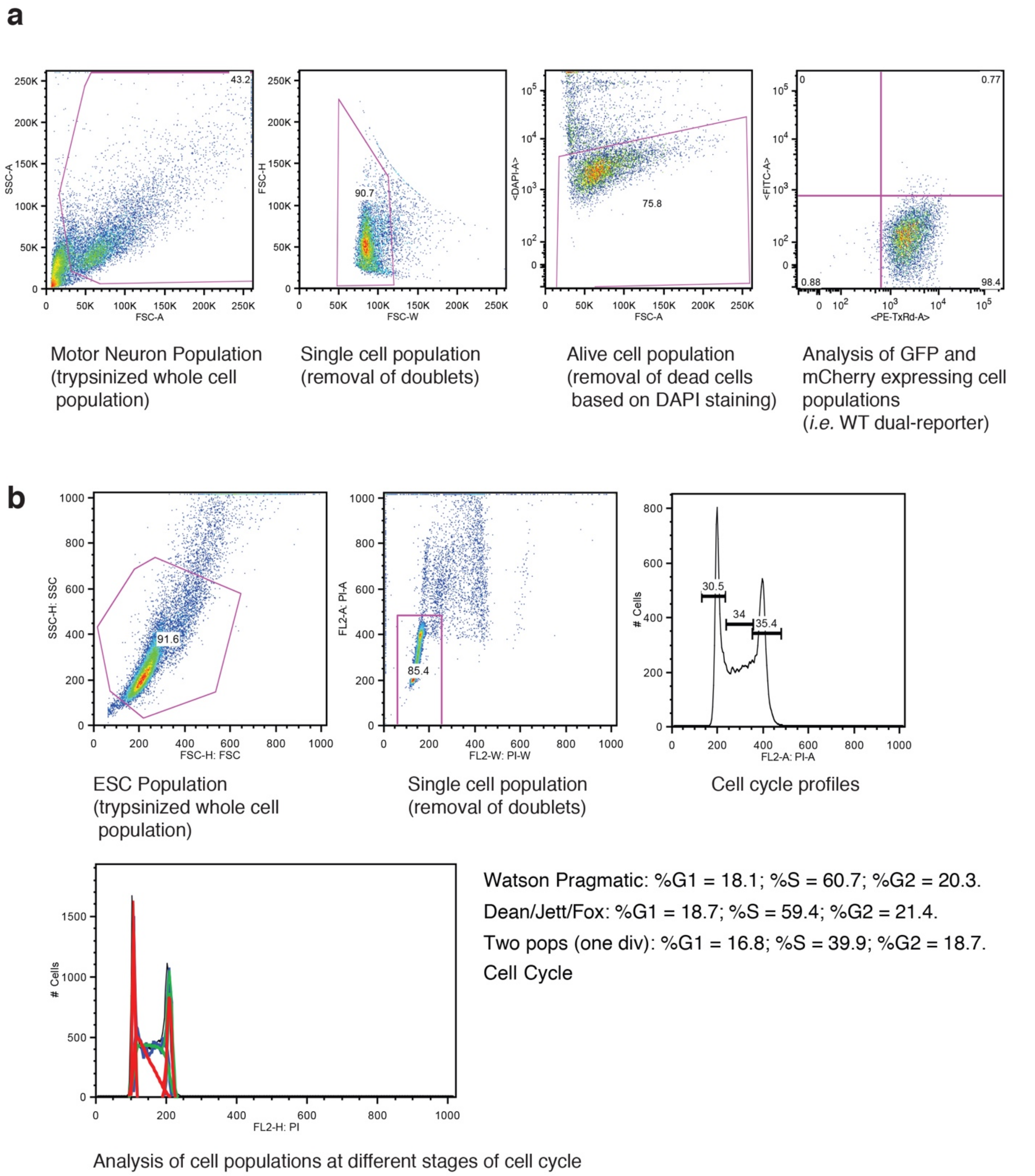

Supplementary Fig. 7 | FACS analysis scheme indicating gating of cellular populations. a, Analysis dual *Hox* reporter in MNs. **b,** Analysis of cell cycle in ESCs.

TABLE S1

CRISPR sgRNAs, DONORS, GENOTYPING PRIMERS

<table>
<tr><td colspan="5">sgRNA oligos</td></tr>
<tr><th>Primer Name</th><th>Forward Sequence</th><th>Reverse Sequence</th><th>sgRNA target</th><th>Aim</th></tr>
<tr><td>A5.Cterm.gRNA</td><td>CACCGTTTGGCCGCTCAGATGCTC</td><td>AAACGAGCATCTGAGCGGCCAAAC</td><td>Hoxa5</td><td>Hoxa5-P2A-mCherry knock-in</td></tr>
<tr><td>A7.Cterm.gRNA</td><td>CACCGGCTGACAAGGCGGACGAGG</td><td>AAACCCTCGTCCGCCTTGTCAGCC</td><td>Hoxa7</td><td>Hoxa7-P2A-eGFP knock-in</td></tr>
<tr><td>HOX5|6 gRNA.3</td><td>CACCGGGACTCGAGCGCCACCCGC</td><td>AAACGCGGGTGGCGCTCGAGTCCC</td><td>CTCF binding site at Hoxa5|6</td><td>CTCF binding site deletion</td></tr>
<tr><td>Hox6|7.gRNA.new</td><td>CACCGGCTCGCTGCTGCCACGCTG</td><td>AAACCAGCGTGGCAGCAGCGAGCC</td><td>CTCF binding site at Hoxa6|7</td><td>CTCF binding site deletion</td></tr>
<tr><td>CTCF.Cterm.gRNA</td><td>CACCGCATGATGGACCGGTGATGC</td><td>AAACGCATCACCGGTCCATCATGC</td><td>Ctcf</td><td>CTCF C-terminus FLAG tag knock-in</td></tr>
<tr><td>Maz_ex2_2</td><td>CACCGCCGAATGCGACAGCTTATGT</td><td>AAACACATAAGCTGTCGCATTCGGC</td><td>Maz</td><td>MAZ knock-out</td></tr>
<tr><td>Maz.a56.gRNA1</td><td>CACCGGAGTTGGGTGGAGGCAAGA</td><td>AAACTCTTGCCTCCACCCAACTCC</td><td>MAZ binding site at Hoxa5|6</td><td>MAZ binding site deletion</td></tr>
<tr><td>Maz.d48.gRNA2**</td><td>CACCGGATGCCGCCCATGCCCTGGG</td><td>AAACCCCAGGGCATGGGCGGCATCC</td><td>MAZ binding site at Hoxd4|8</td><td>MAZ binding site deletion</td></tr>
<tr><td>Maz.d48.gRNA3**</td><td>CACCGGCGAGCTGAGGTGGAAGGCG</td><td>AAACCGCCTTCCACCTCAGCTCGCC</td><td>MAZ binding site at Hoxd4|8</td><td>MAZ binding site deletion</td></tr>
</table>

**simultaneously transfected   *G for U6 promoter transcription initiation   *BbsI overhang
*BbsI overhang

<table>
<tr><td colspan="4">Genotyping Primers</td></tr>
<tr><th>Experiment</th><th>Primer Name</th><th>Forward Sequence</th><th>Reverse Sequence</th></tr>
<tr><td>Hoxa5:a7 dual reporter genotyping</td><td>A5.AH1.int.F/A5.AH2.int.R</td><td>GACAATATAGGTGGCCCAGAAG</td><td>GATGAATTAGGGCAACGAGAAC</td></tr>
<tr><td>Hoxa5:a7 dual reporter genotyping</td><td>A7.AH1.int.F/A7.AH2.int.R</td><td>TCCACATCCTAGTCTGCTTGTC</td><td>TCTTAAAGACGCTTTTCCAACTG</td></tr>
<tr><td>Hoxa5:a7 dual reporter genotyping</td><td>A5.AH1.int.F/mCherry.begin.R</td><td>GACAATATAGGTGGCCCAGAAG</td><td>GTACATGAACTGAGGGGACAGG</td></tr>
<tr><td>Hoxa5:a7 dual reporter genotyping</td><td>A7.AH1.int.F/EGFP.begin.R</td><td>TCCACATCCTAGTCTGCTTGTC</td><td>GCAGATGAACTTCAGGGTCAG</td></tr>
<tr><td>Hoxa5:a7 dual reporter genotyping</td><td>mCherry.end.F/A5.out.R</td><td>CAACATCAAGTTGGACATCACC</td><td>CTCAATTCAGTCTTGCCAAATG</td></tr>
<tr><td>Hoxa5:a7 dual reporter genotyping</td><td>EGFP.end.F/A7.out.R</td><td>ACATGGTCCTGCTGGAGTTC</td><td>CCAGAGGACGCAGGAAATTAG</td></tr>
<tr><td>Hoxa5:a7 dual reporter genotyping</td><td>A5.out.F/P2A.R</td><td>TGGTACATCCTAATGGAACTGC</td><td>TCAGCAGAGAGAAGTTTGTTGC</td></tr>
<tr><td>Hoxa5:a7 dual reporter genotyping</td><td>A7.out.F/P2A.R</td><td>AATGGGGTTTGGTGTAAATCTG</td><td>TCAGCAGAGAGAAGTTTGTTGC</td></tr>
<tr><td>Hoxa5:a7 dual reporter genotyping</td><td>A5.out.F/mCherry.begin.R</td><td>TGGTACATCCTAATGGAACTGC</td><td>GTACATGAACTGAGGGGACAGG</td></tr>
<tr><td>Hoxa5:a7 dual reporter genotyping</td><td>A7.out.F/EGFP.begin.R</td><td>AATGGGGTTTGGTGTAAATCTG</td><td>GCAGATGAACTTCAGGGTCAG</td></tr>
<tr><td>Hoxa5:a7 dual reporter genotyping</td><td>pBluescript.out.F1/mCherry.begin.R</td><td>CAAGGCGATTAAGTTGGGTAAC</td><td>GTACATGAACTGAGGGGACAGG</td></tr>
<tr><td>Hoxa5:a7 dual reporter genotyping</td><td>pBluescript.out.F1/EGFP.begin.R</td><td>CAAGGCGATTAAGTTGGGTAAC</td><td>GCAGATGAACTTCAGGGTCAG</td></tr>
<tr><td>FLAG-CTCF knock-in cell line genotyping</td><td>CTCF.CFtag.F1/R1</td><td>AAAAAGGAGCCAGATGCCGA</td><td>GCCGTTTAAACACAGCCCAA</td></tr>
<tr><td>CTCF binding site deletion (Hoxa5|6)</td><td>5|6.494.new2.f/r</td><td>CACCCTTGCACAATTTATGATGA</td><td>GGATACAAAGCCGGGGAAATAA</td></tr>
<tr><td>CTCF binding site deletion (Hoxa6|7)</td><td>HOX6|7.PCR.530.f/r</td><td>TGTACAAACAGTCTCCATGGTG</td><td>GTTCCCTGGCTATGGTTCTTTT</td></tr>
<tr><td>MAZ knock-out</td><td>Maz_ex2_g2_F1/R1</td><td>AAGCGCATCCGGAAGAATCA</td><td>CAGTGGGAGCAGTTGTAGGG</td></tr>
<tr><td>MAZ knock-out</td><td>Maz_ex2_g2_F2/R2</td><td>TCGGGTGCTATGAAGATGCC</td><td>CAGTGGGAGCAGTTGTAGGG</td></tr>
<tr><td>MAZ binding site deletion (Hoxa5|6)</td><td>Maz.a56.geno.F1/R1</td><td>TGACTGGGACATGTACTCGG</td><td>TGGGCTGTAACCTCAATTCGA</td></tr>
<tr><td>MAZ binding site deletion (Hoxa5|6)</td><td>Maz.a56.geno.F2/R2</td><td>TCGGTTCCCTCCTACGTAGG</td><td>GCTCGAGTCCGACTGAACG</td></tr>
<tr><td>MAZ binding site deletion (Hoxd4|8)</td><td>Maz.d48.geno.F1/R1</td><td>TTTCGGTTGTCTGGAGCTTT</td><td>CGGACAAGTGATCACACCAC</td></tr>
<tr><td>MAZ binding site deletion (Hoxd4|8)</td><td>Maz.d48.geno.F2/R2</td><td>GGACTCCTTTTTGCCTCTCC</td><td>CGGACAAGTGATCACACCAC</td></tr>
</table>

<table>
<tr><td colspan="2">Oligos/Donor Plasmid</td></tr>
<tr><td colspan="2">Hoxa5-P2A-mCherry knock-in (pBlueScript SK+ containing AH1-P2A-mCherry-AH2)</td></tr>
<tr><td>A5 arm of homology 1 (AH1)</td><td>2004 bp</td></tr>
<tr><td>P2A.mCherry.cassette</td><td>GGATCCGGCGCAACAAACTTCTCTCTGCTGAAACAAGCCGGAGATGTCGAAGAGAATCCCGGTCCTATGGTGAGCAAGGGCGAGGAGGATAAC
ATGGCCATCATCAAGGAGTTCATGCGCTTCAAGGTGCACATGGAGGGCTCCGTGAACGGCCACGAGTTCGAGATCGAGGGCGAGGGCGAGGGC
CGCCCCTACGAGGGCACCCAGACCGCCAAGCTGAAGGTGACCAAGGGTGGCCCCCTGCCCTTCGCCTGGGACATCCTGTCCCCTCAGTTCATGT
ACGGCTCCAAGGCCTACGTGAAGCACCCCGCCGACATCCCCGACTACTTGAAGCTGTCCTTCCCCGAGGGCTTCAAGTGGGAGCGCGTGATGAA
CTTCGAGGACGGCGGCGTGGTGACCGTGACCCAGGACTCCTCCCTGCAGGACGGCGAGTTCATCTACAAGGTGAAGCTGCGCGGCACCAACTTC
CCCTCCGACGGCCCCGTAATGCAGAAGAAGACCATGGGCTGGGAGGCCTCCTCCGAGCGGATGTACCCCGAGGACGGCGCCCTGAAGGGCGAG
ATCAAGCAGAGGCTGAAGCTGAAGGACGGCGGCCACTACGACGCTGAGGTCAAGACCACCTACAAGGCCAAGAAGCCCGTGCAGCTGCCCGGC
GCCTACAACGTCAACATCAAGTTGGACATCACCTCCCACAACGAGGACTACACCATCGTGGAACAGTACGAACGCGCCGAGGGCCGCCACTCC
ACCGGCGGCATGGACGAGCTGTACAAGGAATTCTAA</td></tr>
<tr><td>A5 arm of homology 2 (AH2)</td><td>2225 bp</td></tr>
<tr><td colspan="2">Hoxa7-P2A-eGFP knock-in (pBlueScript SK+ containing AH1-P2A-eGFP-AH2)</td></tr>
<tr><td>A7 arm of homology 1 (AH1)</td><td>1698 bp</td></tr>
<tr><td>P2A.eGFP.cassette</td><td>GGATCCGGCGCAACAAACTTCTCTCTGCTGAAACAAGCCGGAGATGTCGAAGAGAATCCCGGTCCTGTGAGCAAGGGCGAGGAGCTGTTCACC
GGGGTGGTGCCCATCCTGGTCGAGCTGGACGGCGACGTAAACGGCCACAAGTTCAGCGTGTCCGGCGAGGGCGAGGGCGATGCCACCTACGGC
AAGCTGACCCTGAAGTTCATCTGCACCACCGGCAAGCTGCCCGTGCCCTGGCCCACCCTCGTGACCACCCTGACCTACGGCGTGCAGTGCTTCAG
CCGCTACCCCGACCACATGAAGCAGCACGACTTCTTCAAGTCCGCCATGCCCGAAGGCTACGTCCAGGAGCGCACCATCTTCTTCAAGGACGAC
GGCAACTACAAGACCCGCGCCGAGGTGAAGTTCGAGGGCGACACCCTGGTGAACCGCATCGAGCTGAAGGGCATCGACTTCAAGGAGGACGGC
AACATCCTGGGGCACAAGCTGGAGTACAACTACAACAGCCACAACGTCTATATCATGGCCGACAAGCAGAAGAACGGCATCAAGGTGAACTTC
AAGATCCGCCACAACATCGAGGACGGCAGCGTGCAGCTCGCCGACCACTACCAGCAGAACACCCCCATCGGCGACGGCCCCGTGCTGCTGCCC
GACAACCACTACCTGAGCACCCAGTCCGCCCTGAGCAAAGACCCCAACGAGAAGCGCGATCACATGGTCCTGCTGGAGTTCGTGACCGCCGCCG
GGATCACTCTCGGCATGGACGAGCTGTACAAGGAATTCTAA</td></tr>
<tr><td>A7 arm of homology 2 (AH2)</td><td>2323 bp</td></tr>
<tr><td colspan="2">CTCF C-terminus FLAG tag knock-in (ssDNA oligo)</td></tr>
<tr><td>CTCF.Cftag.oligo</td><td>GCTCAGGCAGCCACCACAGACGCCCCCAACGGAGACCTCACGCCTGAGATGATCCTCAGCATGATGGACCGGGGATCCGCTGACTACAAGGAT
GACGACGATAAGTGATGCTGGGGCCTTGCTCGGCACCAGGACTATTGGGCTGTGTTTAAACGGCCCAAATCTTAATTTTTCTCTTTTTTTTCTTTG
GCTTTGGGAAC</td></tr>
</table>

Table S1. List of CRISPR sgRNAs, donors, and genotyping primers

TABLE S2

OLIGOS, ANTIBODIES

RT-qPCR Primers

Primer Name	Forward Sequence	Reverse Sequence
Hoxa1	ACCAAGAAGCCTGTCGTTCC	TAGCCGTACTCTCCAACTTTCC
Hoxa2	CCTGGATGAAGGAGAAGAAGG	GTTGGTGTACGCGGTTCTCA
Hoxa3	TCAAGGCAGAACACTAAGCAGA	ATAGGTAGCGGTTGAAGTGGAA
Hoxa4	TGTACCCCTGGATGAAGAAGAT	AAGACTTGCTGCCGGGTATAG
Hoxa5	TGTACGTGGAAGTGTTCCTGTC	GTCACAGTTTTCGTCACAGAGC
Hoxa6	ACCGACCGGAAGTACACAAG	AGGTAGCGGTTGAAGTGGAAT
Hoxa7	GAAGCCAGTTTCCGCATCTAC	CTTCTCCAGTTCCAGCGTCT
Hoxa9	TCCCTGACTGACTATGCTTGTG	ATCGCTTCTTCCGAGTGGAG
Hoxa10	GAAGAAACGCTGCCCTTACAC	TTTCACTTGTCTGTCCGTGAG
Hoxa11	CGAGAGTTCTTCTTCAGCGTCT	TGGAGCCTTAGAGAAGTGGATT
Hoxa13	GCGGTGTCCATGTACTTGTC	GCTGCCCTACGGCTACTTC
Hoxd3	CTACCCTTGGATGAAGAAGGTG	TCAGACAGACACAGGGTGTGA
Hoxd4	CTACCCTTGGATGAAGAAGGTG	TTCTAGGACTTGCTGTCTGGTG
Hoxd8	GCTCGTCTCCTTCTCAAATGTT	GCGACTGTAGGTTTGTCTTCCT
Hoxd9	CAGCAACTTGACCCAAACAAC	TGGTATTTGGTGTAGGGACAGC
Hoxc4	AGCAAGCAACCCATAGTCTACC	GCGGTTGTAATGAAACTCTTTCTC
Hoxc5	CACAGATTTACCCGTGGATGAC	CTTTCTCGAGTTCCAGGGTCT
Hoxc6	TAGTTCTGAGCAGGGCAGGA	CGAGTTAGGTAGCGGTTGAAGT
Hoxc8	GTAAATCCTCCGCCAACACTAA	CGCTTTCTGGTCAAATAAGGAT
Hoxc9	GCAAGCACAAAGAGGAGAAGG	CGTCTGGTACTTGGTGTAGGG
Gapdh	CAAGCTCATTTCCTGGTATGAC	CTCCTGTTATTATGGGGGTCTG
Pou5f1	CACTCACATCGCCAATCAGC	GGGCAGAGGAAAGGATACAG
Gfp	AGCTGACCCTGAAGTTCATCTG	GGACTTGAAGAAGTCGTGCTG
18SrRNA	GTAACCCGTTGAACCCCATT	CCATCCAATCGGTAGTAGCG
Hb9	GAACACCAGTTCAAGCTCAACA	CTCTTCCGTCTTCTCCTCACTG
Lhx3	CGTAGCCTCTAAATGCGAGA	TGGCAAAGGTGTCTGTTCAC
Isl1	GTTGGAGAAAGTGGGAAATGAC	TAGAACAGACTTCATGCGCTTC

* Primer designs of *HoxA* and *HoxC* clusters are from the previous study [13].

ChIP-qPCR Primers

Primer Name	Forward Sequence	Reverse Sequence
Ch.Dm.Ubx.TSS (spike-in)	CTGAGACGGGCTAAAAGTCG	AGCACAGAAAGCGAGGAAAC
Maz.Hoxd48.chip	TTTCTAGGGCTGGTGGTGTC	CACCCACCCTTGTCTGATG
Maz.chipqpcr.cntrl	CAAAGGTCCCCCATACCCAC	CTAGTCGGCCATCACTGCAA

Antibodies

Antibody	Company	Catalogue #	Application	Dilutions
CTCF	Millipore	07-729	ChIP, CUT&RUN, Western Blot (WB)	ChIP: 5ul/100ug chromatin, CUT&RUN: 2ug/200K cells, WB: 1:1000
MAZ	Abcam	ab85725	ChIP, Western Blot (WB)	ChIP: 8ug/100ug chromatin, WB: 1:1000
H3K27me3	Cell Signalling	9733	ChIP, CUT&RUN	ChIP: 5ug/50ug chromatin, CUT&RUN: 2ug/200K cells
H3K4me3	Abcam	ab8580	ChIP, CUT&RUN	ChIP: 5ug/50ug chromatin, CUT&RUN: 2ug/200K cells
RAD21	Abcam	ab992	ChIP, CUT&RUN	ChIP: 5ug/100ug chromatin, CUT&RUN: 2ug/200K cells
RAD21	Abcam	ab154769	Western Blot (WB)	WB: 1:1000
CAS9	Millipore	MAC133-clone 7A9	Western Blot (WB)	WB: 1:1000
HB9	Developmental Hybridoma Bank	N/A	Western Blot (WB)	WB: 1:150
GAPDH	Cell Signalling	D16H11-5174	Western Blot (WB)	WB: 1:1000
B-TUBULIN	Abcam	ab6046	Western Blot (WB)	WB: 1:10000
FLAG	Sigma	F3165	Western Blot (WB)	WB: 1:1000
ISL1/2	Developmental Hybridoma Bank	N/A	Western Blot (WB)	WB: 1:250
LHX3	Developmental Hybridoma Bank	N/A	Western Blot (WB)	WB: 1:250
Histone H3	Abcam	ab1791	Western Blot (WB)	WB: 1:5000
SMC1	Bethyl Laboratories	A300-055A	Western Blot (WB)	WB: 1:1000
H2Av	Active Motif	39715	ChIP	0.2ul/ ug Drosophila chromatin per ChIP
STAG1 / SA1	Abcam	ab4457	Western Blot (WB)	WB: 1:1000
VINCULIN	Cell Signaling	13901	Western Blot (WB)	WB: 1:1000

Table S2. List of oligos and antibodies

TABLE S3

CRISPR LIBRARY PREPARATION PRIMERS, PLASMIDS

**Primers for CRISPR Library Preparation**	
**PCR1 primers for amplification of gRNAs from genomic DNA***	
**Primer Name**	**Sequence**
lentiCRISPR.F1	AATGGACTATCATATGCTTACCGTAACTTGAAAGTATTTCG
lentiCRISPR.R1	CTTTAGTTTGTATGTCTGTTGCTATTATGTCTACTATTCTTTCC
**PCR2 primers for deep sequencing***	
**Primer Name**	**Sequence**
IlluP5.1.bar.F1	AATGATACGGCGACCACCGAGATCTACACTCTTTCCCTACACGACGCTCTTCCGATCTtAAGTAGAGtcttgtggaaaggacgaaacaccg
IlluP5.2.bar.F2	AATGATACGGCGACCACCGAGATCTACACTCTTTCCCTACACGACGCTCTTCCGATCTatACACGATCtcttgtggaaaggacgaaacaccg
IlluP5.3.bar.F3	AATGATACGGCGACCACCGAGATCTACACTCTTTCCCTACACGACGCTCTTCCGATCTgatCGCGCGGTtcttgtggaaaggacgaaacaccg
IlluP5.4.bar.F4	AATGATACGGCGACCACCGAGATCTACACTCTTTCCCTACACGACGCTCTTCCGATCTcgatCATGATCGtcttgtggaaaggacgaaacaccg
IlluP5.5.bar.F5	AATGATACGGCGACCACCGAGATCTACACTCTTTCCCTACACGACGCTCTTCCGATCTtcgatCGTTACCAtcttgtggaaaggacgaaacaccg
IlluP5.6.bar.F6	AATGATACGGCGACCACCGAGATCTACACTCTTTCCCTACACGACGCTCTTCCGATCTatcgatTCCTTGGTtcttgtggaaaggacgaaacaccg
IlluP5.7.bar.F7	AATGATACGGCGACCACCGAGATCTACACTCTTTCCCTACACGACGCTCTTCCGATCTgatcgatAACGCATTtcttgtggaaaggacgaaacaccg
IlluP5.8.bar.F8	AATGATACGGCGACCACCGAGATCTACACTCTTTCCCTACACGACGCTCTTCCGATCTcgatcgatACAGGTATtcttgtggaaaggacgaaacaccg
IlluP5.9.bar.F9	AATGATACGGCGACCACCGAGATCTACACTCTTTCCCTACACGACGCTCTTCCGATCTacgatcgatAGGTAAGGtcttgtggaaaggacgaaacaccg
IlluP5.1.bar.F10	AATGATACGGCGACCACCGAGATCTACACTCTTTCCCTACACGACGCTCTTCCGATCTtAACAATGGtcttgtggaaaggacgaaacaccg
IlluP5.2.bar.F11	AATGATACGGCGACCACCGAGATCTACACTCTTTCCCTACACGACGCTCTTCCGATCTatACTGTATCtcttgtggaaaggacgaaacaccg
IlluP5.3.bar.F12	AATGATACGGCGACCACCGAGATCTACACTCTTTCCCTACACGACGCTCTTCCGATCTgatAGGTCGCAtcttgtggaaaggacgaaacaccg
IlluminaP7.R	CAAGCAGAAGACGGCATACGAGATGTGACTGGAGTTCAGACGTGTGCTCTTCCGATCTtctactattctttcccctgcactgt

Blue: Illumina adaptors including P5 and P7 regions

Red: Stagger to increase library complexity

Black: Barcode for multiplexing

Green: Priming sites

* Primer designs are based on the previous study [14].

**Plasmids/CRISPR Libraries**	
**Plasmid/Library Name**	**Addgene Numbers**
lentiCas9-blast	Addgene: 52962
lentiGuide-puro	Addgene: 52963
pSpCas9(BB)-2A-GFP (PX458)	Addgene: 48138
GeCKO mouse library v2 (A, B)	Addgene: 1000000053
CbetaS-pA (CβF)	Addgene: 32104

Table S3. List of primers for CRISPR library preparation and plasmids

TABLE S4

CUT-OFF VALUES FOR TRANSCRIPTION FACTOR ACTIVITY AT HI-C LOOP ANCHORS

**Cut-off Values for Transcription Factor Activity at Hi-C Loop Anchors**		
	**Embryonic Stem Cells (ESCs)**	**Motor Neurons (MNs)**
CTCF ChIP-seq	30	40
MAZ ChIP-seq	7.5	8

Table S4. Cut-off values for transcription factor activity at Hi-C loop anchors in ESCs and MNs

TABLE S5

OLIGOS FOR EMSA PROBES

EMSA Probes	
**Primer Name**	**Sequence**
maz.hoxa56.WT.emsa.F	GGAGTTGGGTGGAGGCAAGAGGGGTGGCGCGCGCCCTG
maz.hoxa56.WT.emsa.R	CAGGGCGCGCGCGCCACCCCTCTTGCCTCCACCCAACTCC
maz.hoxa56.MUT.emsa.F	GGAGTTGGGTATTCGCAAGAATTCTGGCGCGCGCCCTG
maz.hoxa56.MUT.emsa.R	CAGGGCGCGCGCGCCAGAATTCTTGCGAATACCCAACTCC
maz.hoxd48.emsa.F	CCTGGGTGGGAGGGGAATGGGAGGACACAAGAGCCACCCGTTCTTCTGCCCCGCCTTCCACCTCAG
maz.hoxd48.emsa.R	CTGAGGTGGAAGGCGGGGCAGAAGAACGGGTGGCTCTTGTGTCCTCCCATTCCCCTCCCACCCAGG
maz.hoxd48.MUT.emsa.F	CCTGGGTGGGAATCCAATGGGAATTCACAAGAGCCACCCGTTCTTCTGATTCGCCTTCCACCTCAG
maz.hoxd48.MUT.emsa.R	CTGAGGTGGAAGGCGAATCAGAAGAACGGGTGGCTCTTGTGAATTCCCATTGGATTCCCACCCAGG

Table S5. List of oligos used as EMSA probes

TABLE S6

4C PRIMERS

4C PRIMERS	
**Primer Name**	**Sequence**
4C-pre5.2-F1	AATGATACGGCGACCACCGAGATCTACACTCTTTCCCTACACGACGCTCTTCCGATCTTCGCTTAGTATCTGTGAATGCAGATC
4C-pre5.2-F2	AATGATACGGCGACCACCGAGATCTACACTCTTTCCCTACACGACGCTCTTCCGATCTAGAAGTAGTATCTGTGAATGCAGATC
4C-pre5.2-F3	AATGATACGGCGACCACCGAGATCTACACTCTTTCCCTACACGACGCTCTTCCGATCTAGCGACAGTATCTGTGAATGCAGATC
4C-pre5.2-F4	AATGATACGGCGACCACCGAGATCTACACTCTTTCCCTACACGACGCTCTTCCGATCTCCAATTAGTATCTGTGAATGCAGATC
4C-pre5.2-F5	AATGATACGGCGACCACCGAGATCTACACTCTTTCCCTACACGACGCTCTTCCGATCTCCGTAGAGTATCTGTGAATGCAGATC
4C-pre5.2-F6	AATGATACGGCGACCACCGAGATCTACACTCTTTCCCTACACGACGCTCTTCCGATCTGGACTTAGTATCTGTGAATGCAGATC
4C-pre5.2-F7	AATGATACGGCGACCACCGAGATCTACACTCTTTCCCTACACGACGCTCTTCCGATCTTATAGCAGTATCTGTGAATGCAGATC
4C-pre5.2-F8	AATGATACGGCGACCACCGAGATCTACACTCTTTCCCTACACGACGCTCTTCCGATCTAACTAGAGTATCTGTGAATGCAGATC
4C-pre5.2-F9	AATGATACGGCGACCACCGAGATCTACACTCTTTCCCTACACGACGCTCTTCCGATCTCATGACAGTATCTGTGAATGCAGATC
4C-pre5.2-F10	AATGATACGGCGACCACCGAGATCTACACTCTTTCCCTACACGACGCTCTTCCGATCTGTGTATAGTATCTGTGAATGCAGATC
4C-pre5.2-F11	AATGATACGGCGACCACCGAGATCTACACTCTTTCCCTACACGACGCTCTTCCGATCTATCTGCAGTATCTGTGAATGCAGATC
4C-pre5.2-F12	AATGATACGGCGACCACCGAGATCTACACTCTTTCCCTACACGACGCTCTTCCGATCTTTGGTCAGTATCTGTGAATGCAGATC
4C-pre5.2-R	CAAGCAGAAGACGGCATACGAGATAGGTCGCAGTGACTGGAGTTCAGACGTGTGCTCTTCCGATCTAGGCGGGATTCTGAAGTT

Red: Illumina adapter

Black: Barcodes

Green: Primers

*4C-seq primer designs are based on the previous study [13].

Table S6. List of primers for 4C-seq

Supplementary Dataset Captions

Supplementary Dataset 1. (separate file)

Essential genes in ESCs (**Supplementary Dataset 1a**) and essential/differentiation related genes in MNs (**Supplementary Dataset 1b**) identified via genome-wide CRISPR screens

Supplementary Dataset 2. (separate file)

Candidates identified to influence CTCF boundary in genome-wide CRISPR screens

Supplementary Dataset 3. (separate file)

Common candidates identified to influence CTCF boundary in independent sub-library screens

Supplementary Dataset 4. (separate file)

Peptide counts in native FLAG-CTCF ChIP-MS in ESCs and MNs

Supplementary Dataset 5. (separate file)

Common candidates identified to influence CTCF boundary in genome-wide CRISPR screens and ChIP-mass spectrometry

Supplementary Dataset 6. (separate file)

List of sgRNAs in the custom library

Supplementary Dataset 7. (separate file)

List of genes identified in secondary screens in WT background

Supplementary Dataset 8. (separate file)

List of genes identified in secondary screens in CTCF site deletion background

Supplementary Dataset 9. (separate file)

List of genes uniquely identified in secondary screens in WT background compared to CTCF site deletion background

Supplementary Dataset 10. (separate file)

RNA-seq expression values in WT vs MAZ KO ESCs for differentially expressed genes

Supplementary Dataset 11. (separate file)

RNA-seq expression values in WT vs MAZ KO MNs for differentially expressed genes

Supplementary Dataset 12. (separate file)

RT-qPCR data and analysis in WT, MAZ KO, and CTCF (Δ5|6:6|7) ESCs and MNs

Supplementary Dataset 13. (separate file)

RNA-seq expression values in WT vs MAZ (Δ5|6) ESCs for differentially expressed genes

Supplementary Dataset 14. (separate file)

RNA-seq expression values in WT vs MAZ (Δ5|6) MNs for differentially expressed genes

References

1. Duboule, D. & Morata, G. Colinearity and Functional Hierarchy among Genes of the Homeotic Complexes. *Trends in Genetics* **10**, 358-364 (1994).
2. Kmita, M. & Duboule, D. Organizing axes in time and space; 25 years of colinear tinkering. *Science* **301**, 331-3 (2003).
3. Doench, J.G. *et al.* Optimized sgRNA design to maximize activity and minimize off-target effects of CRISPR-Cas9. *Nat Biotechnol* **34**, 184-191 (2016).
4. Kagey, M.H. *et al.* Mediator and cohesin connect gene expression and chromatin architecture. *Nature* **467**, 430-5 (2010).
5. Zuin, J. *et al.* A cohesin-independent role for NIPBL at promoters provides insights in CdLS. *PLoS Genet* **10**, e1004153 (2014).
6. Song, J. *et al.* Two consecutive zinc fingers in Sp1 and in MAZ are essential for interactions with cis-elements. *J Biol Chem* **276**, 30429-34 (2001).
7. Busslinger, G.A. *et al.* Cohesin is positioned in mammalian genomes by transcription, CTCF and Wapl. *Nature* **544**, 503-507 (2017).
8. Xiao, T., Li, X. & Felsenfeld, G. The Myc-associated zinc finger protein (MAZ) works together with CTCF to control cohesin positioning and genome organization. *Proc Natl Acad Sci U S A* **118**(2021).
9. Thiecke, M.J. *et al.* Cohesin-Dependent and -Independent Mechanisms Mediate Chromosomal Contacts between Promoters and Enhancers. *Cell Rep* **32**, 107929 (2020).
10. Wellik, D.M. Hox genes and kidney development. *Pediatr Nephrol* **26**, 1559-65 (2011).
11. Di-Poï, N., Zákány, J. & Duboule, D. Distinct Roles and Regulations for Hoxd Genes in Metanephric Kidney Development. *PLoS Genetics* **preprint**(2005).

12. Cavalheiro, G.R., Pollex, T. & Furlong, E.E. To loop or not to loop: what is the role of TADs in enhancer function and gene regulation? *Curr Opin Genet Dev* **67**, 119-129 (2021).

13. Narendra, V.R., P.P. ; An, D.; Raviram, R.; Skok, J.A.; Mazzoni,E.O.; Reinberg, D. CTCF establishes discrete functional chromatin domains at the Hox clusters during differentiation. *Science* **347**, 1017-21 (2015).

14. Shalem, O. *et al.* Genome-scale CRISPR-Cas9 knockout screening in human cells. *Science* **343**, 84-7 (2014).
