# Supplementary material for: CRISPR and biochemical screens identify MAZ as a cofactor in CTCF-mediated insulation at Hox clusters
Source: Nat Genet. 2022 Feb 10;54(2):202–12. doi: 10.1038/s41588-021-01008-5 (PMC8837555; doi:10.1038/s41588-021-01008-5)
Supplement: Supplementary file 2 — Reporting Summary [file 41588_2021_1008_MOESM2_ESM.pdf]

nature research

Corresponding author(s): Danny Reinberg
Last updated by author(s): 2021/11/20

Reporting Summary

Nature Research wishes to improve the reproducibility of the work that we publish. This form provides structure for consistency and transparency in reporting. For further information on Nature Research policies, see our Editorial Policies and the Editorial Policy Checklist.

Statistics

For all statistical analyses, confirm that the following items are present in the figure legend, table legend, main text, or Methods section.

n/a	Confirmed	
☐	☒	The exact sample size (*n*) for each experimental group/condition, given as a discrete number and unit of measurement
☐	☒	A statement on whether measurements were taken from distinct samples or whether the same sample was measured repeatedly
☐	☒	The statistical test(s) used AND whether they are one- or two-sided<br>*Only common tests should be described solely by name; describe more complex techniques in the Methods section.*
☒	☐	A description of all covariates tested
☐	☒	A description of any assumptions or corrections, such as tests of normality and adjustment for multiple comparisons
☐	☒	A full description of the statistical parameters including central tendency (e.g. means) or other basic estimates (e.g. regression coefficient) AND variation (e.g. standard deviation) or associated estimates of uncertainty (e.g. confidence intervals)
☐	☒	For null hypothesis testing, the test statistic (e.g. *F*, *t*, *r*) with confidence intervals, effect sizes, degrees of freedom and *P* value noted<br>*Give P values as exact values whenever suitable.*
☒	☐	For Bayesian analysis, information on the choice of priors and Markov chain Monte Carlo settings
☒	☐	For hierarchical and complex designs, identification of the appropriate level for tests and full reporting of outcomes
☒	☐	Estimates of effect sizes (e.g. Cohen's *d*, Pearson's *r*), indicating how they were calculated

Our web collection on statistics for biologists contains articles on many of the points above.

Software and code

Policy information about availability of computer code

Data collection	Next-generation sequencing have been acquired by using Illumina instrumentation and software (HiSeq, Novaseq, Nextseq, and MiSeq) as detailed in GEO database for each experiment (CRISPR screens, ChIPseq, RNAseq, 4C, CUT&RUN, and HiC).
Data analysis	Data analysis tools or softwares used in this study has been published in the literature as described in the methods. The codes will be available upon reasonable request.<br><br>For CRISPR screen analysis, MaGeCK tools (version 0.5.7) were used as described in Methods. PANTHER database was used for Gene Ontology (GO) analysis. Venn diagrams in CRISPR screens were generated by using web-tools at http://genevenn.sourceforge.net were used.<br><br>For RNA-seq analysis, Bowtie 2 (version 2.3.4.1) was utilized for mapping and DEseq2 (version 1.26.0) was used for differential gene expression analysis as described in Methods. PANTHER database was used for Gene Ontology (GO) analysis.<br><br>For ChIP-seq analysis, Bowtie 2 (version 2.3.4.1) was utilized for mapping. Quality filtering and removal of PCR duplicates were performed by using SAMtools (version 1.9). After normalization with Drosophila spike-in counts, Integrative Genomics Viewer (IGV) version 2.4.1477 was used for visualization. MACS (version 1.4.2) was used for narrow peak calling, heat maps were generated using deepTools in R (version 3.1.2), and 'ChIPpeakAnno' package (version 3.20.1) from Bioconductor was used to draw Venn diagrams to visualize overlaps. In addition, BEDTools (version 2.27.1) were also used for the assessment of overlaps. ChIP-seq "bed" file coordinates were converted into "fasta" by using fetch sequences tool within Regulatory Sequence Analysis Tools (RSAT); MEME (version 5.4.1) was used for motif analysis of MAZ in ESCs and MNs, SpaMo (version 5.4.1) was used for distance analysis between CTCF and MAZ motifs in ESCs and MNs, and Tomtom (version 5.4.1) was used as a motif comparison tool.<br><br>For 4C-seq analysis, 4C-ker (version 0.0.0.9000) pipeline was used and near-bait analysis was generated in R by using 4C-ker tools.<br><br>For Hi-C analysis, hic-bench platform (version 0.1) was utilized. For the compartment analysis, the Hi-C interaction bins were divided into A

and B compartments using the first principal component values from HOMER's runHiCpca. Significantly enriched chromatin loops were called using FitHi-C (version 2.0.7). To characterize the loops by CTCF and MAZ ChIP-seq levels, aggregate peak analysis (APA) software was used. The genome sequence that matched the transcription factor motifs of mouse CTCF and MAZ from the Catalog of Inferred Sequence Binding Preferences (CIS-BP) was found from PWMScan. Visualization of Hi-C and associated ChIP-seq data was made with pyGenomeTracks (version 3.5).

For motif analysis at Hi-C loop anchors, the motifFinder feature of Juicer (version 1.5) and Find Individual Motif Occurrences (FIMO) of MEME suite (version 5.2.0) were utilized as described in Methods.

For FACS data processing analysis, FlowJo (version 8.7) was used. GraphPad Prism (version 9.2.0) was used for statistical analysis and bar plot generation.

For RT-qPCR data analysis and ChIP-qPCR data processing, GraphPad Prism (version 9.2.0) was used. In addition, an R package “pcr” has been used in Extended Data Figure 4e-g for statistical analysis.

For manuscripts utilizing custom algorithms or software that are central to the research but not yet described in published literature, software must be made available to editors and reviewers. We strongly encourage code deposition in a community repository (e.g. GitHub). See the Nature Research guidelines for submitting code & software for further information.

Data

Policy information about availability of data

All manuscripts must include a data availability statement. This statement should provide the following information, where applicable:

- Accession codes, unique identifiers, or web links for publicly available datasets
- A list of figures that have associated raw data
- A description of any restrictions on data availability

Sequencing data has been deposited at Gene Expression Omnibus (GSE157139). We used the following publicly available datasets in Fig. 4f and Fig S11h-i pertaining to CTCF-degron ESCs: GEO GSE98671 and GSE156868. The list of differentially expressed genes in CTCF-degron ESCs used in Fig. 2k was reported (42). Proteomics data has been deposited to PRIDE under accession number..... Source data are provided with this paper.

Field-specific reporting

Please select the one below that is the best fit for your research. If you are not sure, read the appropriate sections before making your selection.

☒ Life sciences ☐ Behavioural & social sciences ☐ Ecological, evolutionary & environmental sciences

For a reference copy of the document with all sections, see nature.com/documents/nr-reporting-summary-flat.pdf

Life sciences study design

All studies must disclose on these points even when the disclosure is negative.

Sample size	Sample sizes used in each experiment has been indicated in the figures, figure legends, tables, main text or supplementary materials. When it is possible, minimum of three biological replicates have been used, standard deviation and significance levels have been shown. Sample sizes for each method (i.e. CRISPR screens, RNAseq, ChIPseq, 4C, HiC, CUT&RUN...etc.) are determined by general variability of the data in literature and the samples sizes used are sufficient to draw conclusions. Additionally, we note that our conclusions were evaluated through multiple methods for a given hypothesis.
Data exclusions	Data processing has been described in the supplementary materials. Data exclusion is not performed.
Replication	The number replicates per experiment has been described in the figures, figure legends, tables, main text or supplementary materials. When it is possible, the results have been reproduced as biological triplicates. If it is not possible/applicable, it has been indicated. In the case of less replicates, experiments were successful and the results were sufficient to draw the conclusions for a particular method in accordance with literature. In addition, we assured using multiple methods to assess a given hypothesis in the cases where less replicates are reported.
Randomization	Randomization is applicable throughout genetic screen processes particularly to keep the representation of each single guide RNA within the cell population in an unbiased manner. The methods have been detailed in Supplementary Materials. In other parts of this study, randomization is not applicable as we evaluate wild type cells versus specific genotypes. Nevertheless, any specimen preparation or selection from a large pool of cells/DNA/RNA has always been randomized.
Blinding	Blinding is not applicable during most of the experimental setups especially for MAZ or CTCF motif deletions at specific Hox loci. As the genotypes of the cells are known during the cell culture and initial sample preparation, blinding is not applicable yet all cells with different genotypes and the samples derived from cells were treated simultaneously in each experiment. When possible, blinding is applied during data processing and outcome has been evaluated unbiasedly. Blinding was applied during mice experiments throughout the staining and analysis.

Reporting for specific materials, systems and methods

We require information from authors about some types of materials, experimental systems and methods used in many studies. Here, indicate whether each material, system or method listed is relevant to your study. If you are not sure if a list item applies to your research, read the appropriate section before selecting a response.

Materials & experimental systems

n/a	Involved in the study
☐	☒ Antibodies
☐	☒ Eukaryotic cell lines
☒	☐ Palaeontology and archaeology
☐	☒ Animals and other organisms
☒	☐ Human research participants
☒	☐ Clinical data
☒	☐ Dual use research of concern

Methods

n/a	Involved in the study
☐	☒ ChIP-seq
☐	☒ Flow cytometry
☒	☐ MRI-based neuroimaging

Antibodies

Antibodies used

Antibodies used are listed in Table S2 in Supplementary Materials (also see below):

Antibody, Company, Catalogue Number, Application, Clonal Information
CTCF, Millipore, 07-729, ChIP & Western Blot, rabbit polyclonal antibody
MAZ, Abcam, ab85725, ChIP & Western Blot, rabbit polyclonal antibody
H3K27me3, Cell Signalling, 9733, ChIP, rabbit polyclonal antibody
H3K4me3, Abcam ab8580, ChIP, rabbit polyclonal antibody
RAD21, Abcam, ab992, ChIP, rabbit polyclonal antibody
RAD21, Abcam, ab154769, Western Blot, rabbit polyclonal antibody
CAS9, Millipore, MAC133-clone 7A9, Western Blot, mouse monoclonal antibody (clone 7A9)
HB9, Developmental Hybridoma Bank, N/A, Western Blot, mouse monoclonal antibody (81.5C10)
GAPDH, Cell Signalling, D16H11-5174, Western Blot, rabbit monoclonal antibody (5174)
B-TUBULIN, Abcam, ab6046, Western Blot, rabbit polyclonal antibody
FLAG, Sigma, F3165, Western Blot, mouse monoclonal antibody (clone M2)
ISL1/2, Developmental Hybridoma Bank, N/A, Western Blot, mouse monoclonal antibody (39.4D5)
LHX3, Developmental Hybridoma Bank, N/A, Western Blot, mouse monoclonal antibody (67.4E12)
Histone H3, Abcam, ab1791, Western Blot, rabbit polyclonal antibody
SMC1, Bethyl Laboratories, A300-055A, Western Blot, rabbit polyclonal antibody
H2Av, Active Motif, 39715, ChIP, rabbit polyclonal antibody
STAG1 / SA1, Abcam, ab4457, Western Blot, goat polyclonal antibody
VINCULIN, Cell Signaling, 13901, Western Blot, rabbit monoclonal antibody (E1E9V)

Validation

Each primary antibody has been validated through manufacturer's information, previous publications referring to the use of antibody for a ChIP or Western blot, and independently by our studies as follows when applicable:

1. If possible, knock-out or degron of target protein has been used to ensure the specificity of protein for ChIP or Western blot (i.e. MAZ, CTCF, RAD21, SMC1). For MAZ, the knock-out lines generated in this manuscript ensure the validation of MAZ antibody, indicating the loss of two isoforms on western blot in MAZ KO and the loss of ChIP-seq signal in MAZ KO. CTCF, RAD21 and SMC1 antibodies were validated with the degron cell lines generated for each in the literature (CTCF degron: Nora et al. 2017; Cohesin degron: Rao et al. 2017). Although a different antibody was used in CTCF degron study, we additionally confirmed the validity of CTCF antibody in the same degron cell lines (not shown).

2. When applicable, different cell types/lines expressing the protein or not have been used for the specificity of signal (i.e. HB9, ISL1/2, LHX3, FLAG, CAS9). HB9, ISL1/2 and LHX3 antibodies for motor neuron markers were validated by western blot using the differentiation of ESCs into MNs, where ESCs do not express them while MNs express (not shown). In addition, they were validated in literature by immunofluorescence and immunohistochemistry as indicated by the manufacturer (Tanabe et al. 1998; Ericson et al. 1997; Ericson et al. 1992; Narendra et al. 2015). The specificity of FLAG antibody is validated with untagged and tagged cell lines through western blot in this manuscript in addition to manufacturer's validation. CAS9 antibody was similarly validated with clones that expressed CAS9 or not in this manuscript in addition to manufacturer's validation.

3. ChIPseq results have been independently checked for de-novo motif discovery to see whether reported motifs for each factor can be retrieved (i.e. MAZ, CTCF, RAD21, SMC1). This is reported in this manuscript for CTCF, MAZ and RAD21.

4. ChIPseq results in comparison to previously published data indicating the patterns of ChIPseq has been informative in validation (i.e. H3K27me3, H3K4me3, CTCF, MAZ, RAD21...etc.). RAD21 antibody (Abcam, ab992) has been validated before (Nora et al. 2020). CTCF, H3K27me3 and H3K4me3 antibodies have been validated in previous publications (Narendra et al. 2015; Narendra et al. 2016). MAZ antibody has also been validated for ChIPseq in several studies (Van Bortle et al. 2014).

5. Purified proteins or protein complexes (if available) could serve for the validation for several antibodies (i.e. MAZ, CTCF, RAD21...etc). MAZ and CTCF proteins purified from 293FT cells, and co-IP experiments related to FLAG-CTCF and FLAG-MAZ also ensure the validation of antibodies.

6. Evaluation of different cell types, manufacturer's information, and previous publications were taken as reference for loading controls and others (i.e. GAPDH, B-TUBULIN, Histone H3, VINCULIN, H2Av). They correspond to the expected sizes indicated by the manufacturer in western blots performed in ESCs, MNs or 293FTs.

Eukaryotic cell lines

Policy information about cell lines

Cell line source(s)	E14 mouse embryonic stem cells (mESCs), Source: ATCC, Identifier: CRL-1821<br>293FTs, Source: Thermo Fisher Scientific, Identifier: R70007<br><br>Cell lines generated in this study have been described in detail in Supplementary Materials (listed below):<br>- Hoxa5-mCherry/Hoxa7-GFP dual-reporter E14 mESCs, Source: This paper<br>- Hoxa5-mCherry/Hoxa7-GFP dual-reporter-Cas9-clone3 E14 mESCs, Source: This paper<br>- MAZ knock-out E14 mESCs, Source: This paper<br>- Hoxa5|6 MAZ motif deleted E14 mESCs, Source: This paper<br>- Hoxd4|8 MAZ motif deleted E14 mESCs, Source: This paper<br>- Hoxa5|6 and Hoxa6|7 CTCF motif deleted mESCs, Source: This paper<br>- Hoxa5|6 and Hoxa6|7 CTCF motif deleted & MAZ knock-out E14 mESCs, Source: This paper<br>- Hb9-GFP reporter E14 mESCs, Source: This paper
Authentication	Identity of 293FTs, mESCs and generated cell lines have been frequently checked by morphological features, qPCRs for cell markers during differentiation from mESCs into MNs, RNAseq expression profiling, or FACS analysis for cell-marker reporters (when applicable), but they have not been authenticated.
Mycoplasma contamination	All cell lines are negative for mycoplasma contamination and they are regularly tested throughout the study.
Commonly misidentified lines<br>(See ICLAC register)	No commonly misidentified cell line was used.

Animals and other organisms

Policy information about studies involving animals; ARRIVE guidelines recommended for reporting animal research

Laboratory animals	C57BL/6 mice (Wild-type versus MAZ Hoxa5|6 motif deleted), postnatal day 0.5, both males and females.
Wild animals	No wild animals were used in this study.
Field-collected samples	No field-collected samples were used in this study.
Ethics oversight	NYU/NYULMC Institutional Biosafety Committee

Note that full information on the approval of the study protocol must also be provided in the manuscript.

ChIP-seq

Data deposition

☒ Confirm that both raw and final processed data have been deposited in a public database such as GEO.

☒ Confirm that you have deposited or provided access to graph files (e.g. BED files) for the called peaks.

Data access links<br>*May remain private before publication.*	We are in the process of deposition of ChIPseq data to GEO database. Currently, GEO accession number (GSE157139) for RNAseq and HiC data is available upon request with a reviewer token. The GEO accession numbers will be provided for ChIPseq similarly asap upon request with a reviewer token.
Files in database submission	ChIPseq data related to each figure with raw and processed files will be available before publication.
Genome browser session<br>(e.g. UCSC)	Not possible to provide at the moment as we utilized Integrative Genomics Viewer (IGV) instead.

Methodology

Replicates	Replicates for each experiment have been described in the figure legend, main text, or supplementary information. When possible, triplicates or duplicates have been used.
Sequencing depth	The following summarized information will be available at GEO for each ChIPseq experiment:<br>Single-end ChIPseq<br>50bp read length<br>~20-30 million total reads per ChIPseq replicate<br>~10-20 million uniquely mapped reads per ChIPseq replicate
Antibodies	Antibodies used are listed in Table S2 in Supplementary Materials, and they will be also indicated in GEO.

Peak calling parameters	Bowtie2 Mapping and Removal of Duplicates with Samtools (mouse):<br>map <- paste(c("/gpfs/share/apps/bowtie2/2.3.4.1/bin/bowtie2 --local --threads 16 -x /gpfs/share/apps/iGenomes/Mus_musculus/UCSC/mm10/Sequence/Bowtie2Index/genome -U " , fastq_path,filelist[[j]], " | ","/gpfs/share/apps/samtools/1.9/bin/samtools view -q 30 -@ ", 16 ," -Sb - > ", bam_path,filelist[[j]],".bam"),collapse="")<br>system(map)<br>sort <- paste(c("/gpfs/share/apps/samtools/1.9/bin/samtools sort -o ", bam_path,filelist[[j]],"_sorted.bam ", bam_path,filelist[[j]],".bam"),collapse = "")<br>system(sort)<br>filt <- paste(c("/gpfs/share/apps/samtools/1.9/bin/samtools rmdup -s ", bam_path,filelist[[j]], "_sorted.bam ", bam_path,filelist[[j]], "_filt.bam"),collapse = "")<br>system(filt)<br><br>Bowtie2 Mapping (Drosophila spike-in):<br>map <- paste(c("/gpfs/share/apps/bowtie2/2.3.4.1/bin/bowtie2 --local --threads 16 -x /gpfs/share/apps/iGenomes/Drosophila_melanogaster/UCSC/dm3/Sequence/Bowtie2Index/genome -U " , fastq_path,filelist[[j]], " | ","/gpfs/share/apps/samtools/1.9/bin/samtools view -q 30 -@ ", 16 ," -Sb - > ", bam_path,filelist[[j]],"_dm.bam"),collapse="")<br>system(map)<br><br>MACS peak calling (default parameters, 5% FDR):<br>bam_t="/gpfs/data/HOK_ChIPseq_1.fastq.gz.bam"<br>bam_c="/gpfs/data/HOK_ChIPseq_input.bam"<br>macs2 callpeak -t $bam_t -c $bam_c -f BAM -g mm -n HOK_"$i"_narrow
Data quality	The methods have been described in Supplementary Materials. For ChIPseq analysis, sequence reads were mapped to mm10 reference genome with Bowtie 2 using default parameters. After normalization with the spike-in Drosophila read counts, normalized ChIP-seq read densities were visualized in Integrative Genomics Viewer (IGV). MACS has been used for peak calling (FDR cut-off: 5%), and total peak numbers for each ChIPseq has been included in the figures or main text. Heat maps were generated using deepTools in R. 'ChIPpeakAnno' package from Bioconductor was used to draw Venn diagrams to visualize the overlap among ChIP-seq samples. The replicates of ChIPseq were assessed similarly by visualizing at IGV, generating heat maps, and comparing peak numbers.
Software	Analysis tools and software have been published in the literature and described in Supplementary Materials (also see above). Bowtie2 (v2.3.4.1), SAMtools (v1.9), BEDTools (v2.27.1), Macs (v1.4.2), deepTools (v3.1.2), ChIPpeakAnno (v3.20.1), IGV (v2.4.14). MEME (v5.4.1), SpaMo (v5.4.1) and Tomtom (v5.4.1) are available at https://meme-suite.org/meme/index.html.<br>RSAT is available at http://pedagogix-tagc.univ-mrs.fr/rsat/fetch-sequences_form.php.

Flow Cytometry

Plots

Confirm that:

☒ The axis labels state the marker and fluorochrome used (e.g. CD4-FITC).

☒ The axis scales are clearly visible. Include numbers along axes only for bottom left plot of group (a 'group' is an analysis of identical markers).

☒ All plots are contour plots with outliers or pseudocolor plots.

☒ A numerical value for number of cells or percentage (with statistics) is provided.

Methodology

Sample preparation	Cells were trypsinized, filtered, and stained with 4,6-diamidino-2-phenylindole (DAPI, Sigma) to eliminate dead cells during analysis of Hoxa5:a7 reporters in ESCs and MNs. Hoxa5:a7 dual fluorescent reporter cells in WT versus other backgrounds were assessed by using single color fluorescent reporters as controls in the same cell type as analyzed (i.e. MNs). Hb9-T2A-GFP reporter cells (not shown) were used as GFP control in MNs. All cell lines have been grown, differentiated, and analyzed at FACS at the same time. For cell cycle analysis, ESCs were fixed in 75% Ethanol, and DNA was stained with propidium iodide (Thermo Fisher Scientific) after RNase A (Thermo Fisher Scientific) treatment.
Instrument	LSRIIUV for the analysis of dual-reporter cell lines, FACS calibur for cell cycle analysis
Software	FlowJo 8.7 was used for all FACS analysis
Cell population abundance	A population of minimum 10 000 cells or 20 000 cells were acquired for each sample, and overall cell population was determined based on forward and side-scattering. The abundances are as below:<br>Hoxa5-mCherry or Hoxa7-GFP: Not detected in ESCs<br>Hoxa5-mCherry positive motor neurons: ~95-100%<br>Hoxa5-mCherry/Hoxa7-GFP dual positive motor neurons: ~5-15 % depending on described genetic perturbations in the study<br>Hb9-GFP positive motor neurons: ~25-30 %<br>Cell cycle analysis: Propidium iodide staining of all cell population

Gating strategy	Gating strategy involved the following for the assessment of dual-reporter sequentially: The selection of single cells, elimination of DAPI positive dead cells to limit the analysis to alive cells, assessing of single-color controls in motor neurons and setting the gates based on them, and finally assessment of the dual-reporter cells in motor neurons (see Fig. S1c).

☒ Tick this box to confirm that a figure exemplifying the gating strategy is provided in the Supplementary Information.
