# Supplementary figures and images for: CRISPR and biochemical screens identify MAZ as a cofactor in CTCF-mediated insulation at Hox clusters
Source: Nat Genet. 2022 Feb 10;54(2):202–12. doi: 10.1038/s41588-021-01008-5 (PMC8837555; doi:10.1038/s41588-021-01008-5)

Figure 5

Figure 5a (Replicate 1)

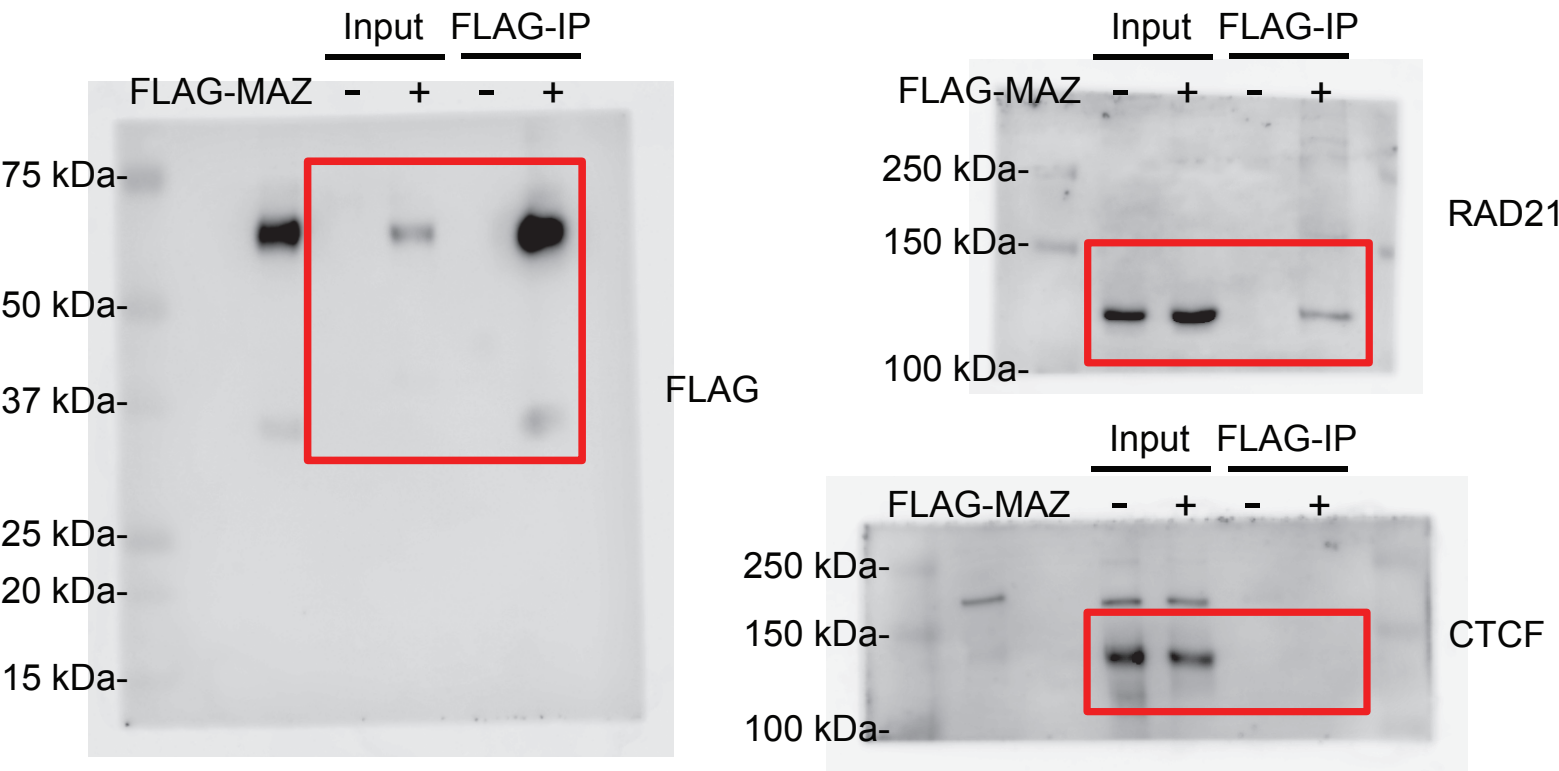

Figure 5a (Replicate 2)

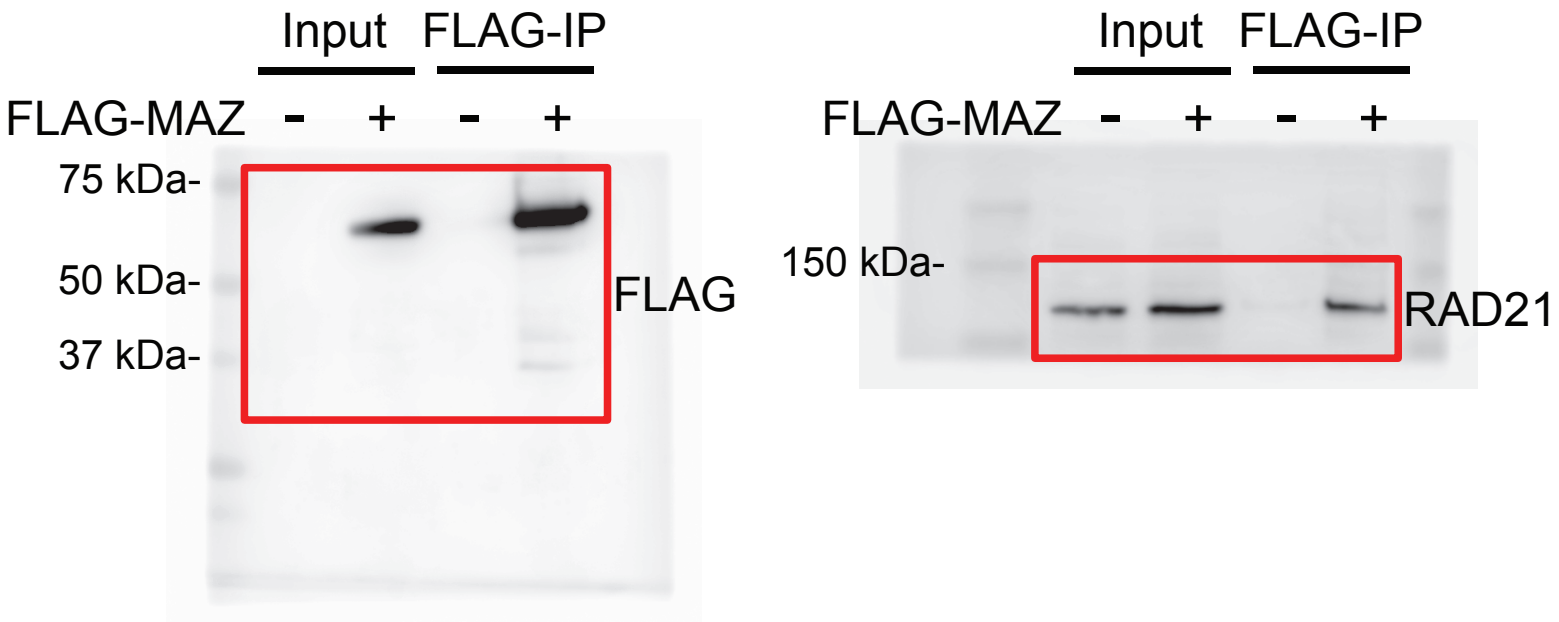

Supplement: Source Data Fig. 5 — Uncropped western blots. [file 41588_2021_1008_MOESM4_ESM.pdf]

Extended Data Figure 1

Extended Data Figure 1e

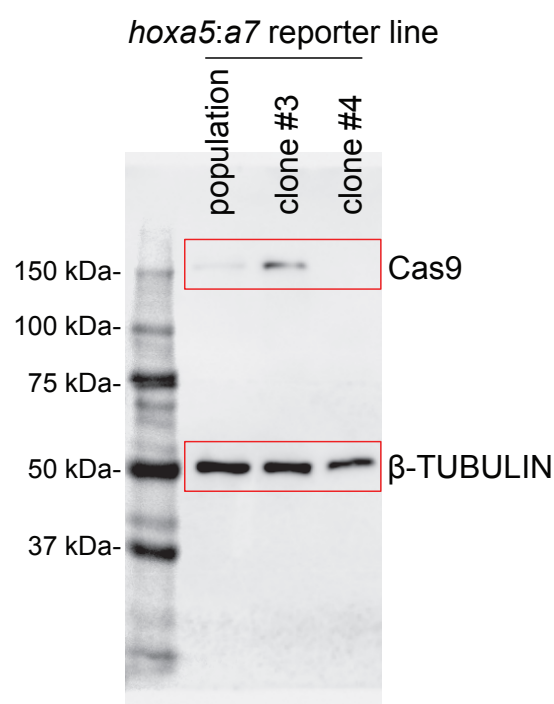

Supplement: Source Data Extended Data Fig. 1 — Uncropped western blots. [file 41588_2021_1008_MOESM5_ESM.pdf]

Extended Data Figure 3

Extended Data Figure 3f

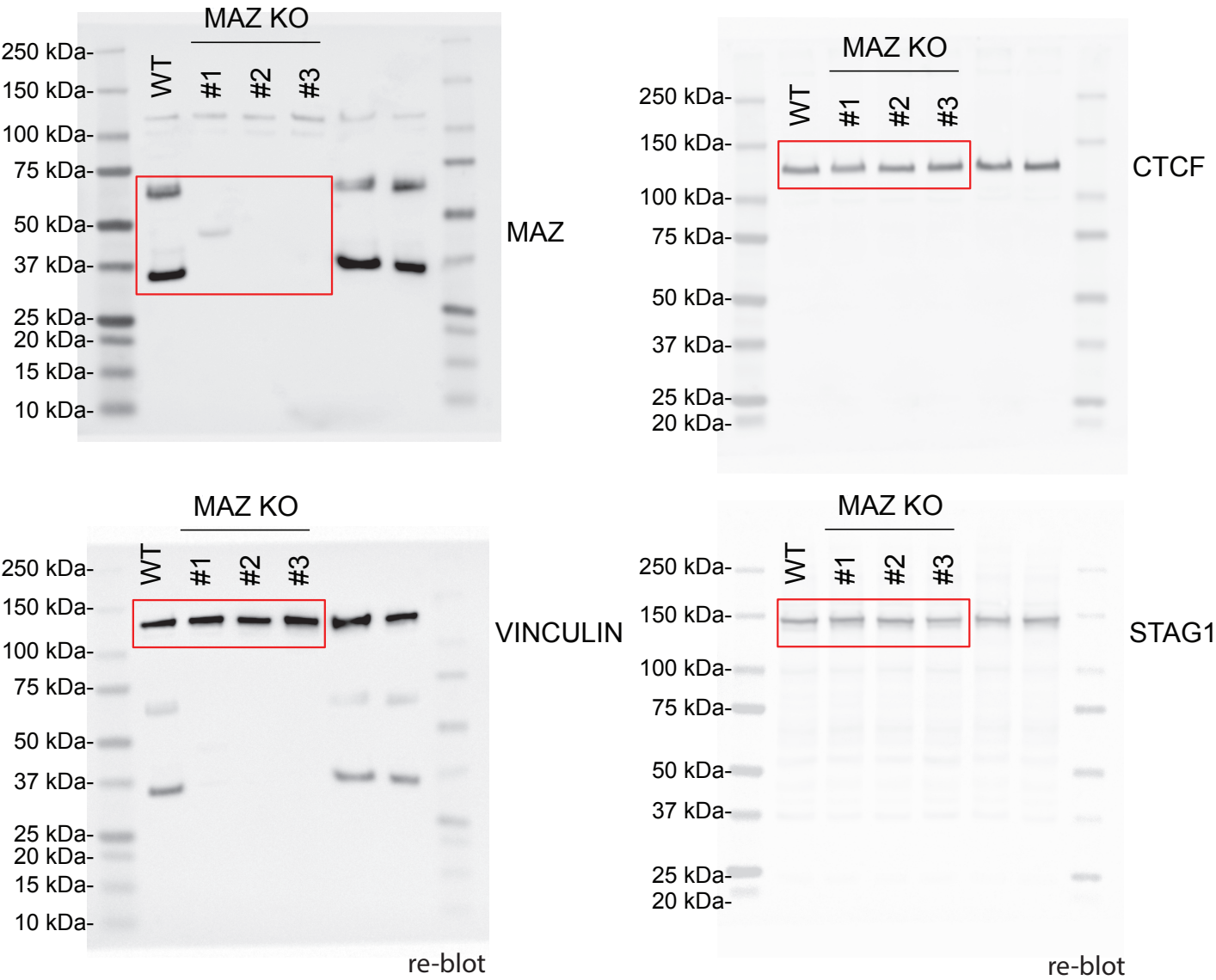

Supplement: Source Data Extended Data Fig. 3 — Uncropped western blots. [file 41588_2021_1008_MOESM7_ESM.pdf]

Extended Data Figure 6

Extended Data Figure 6a

Replicate 1

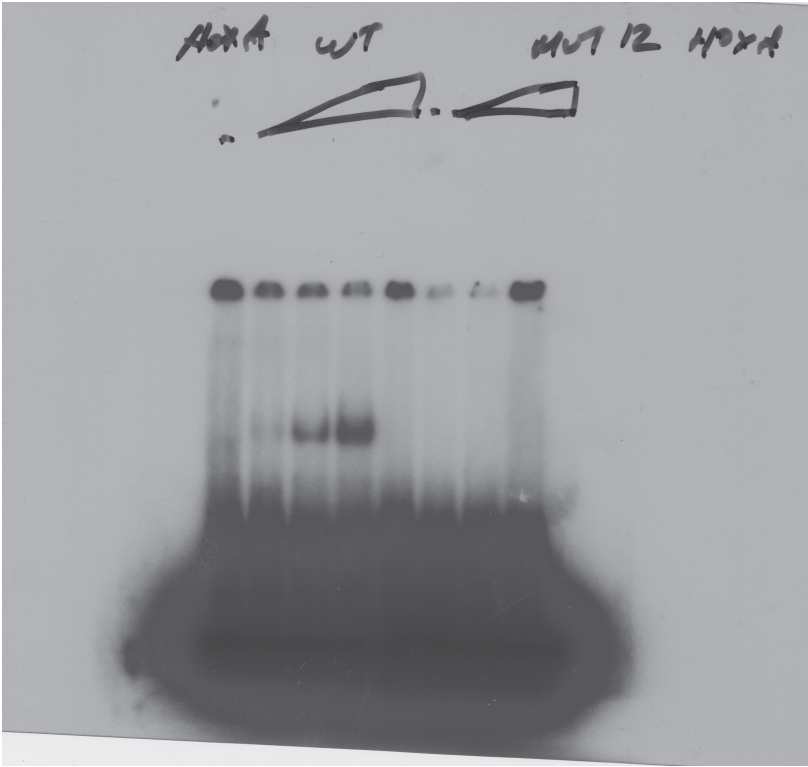

Replicate 2

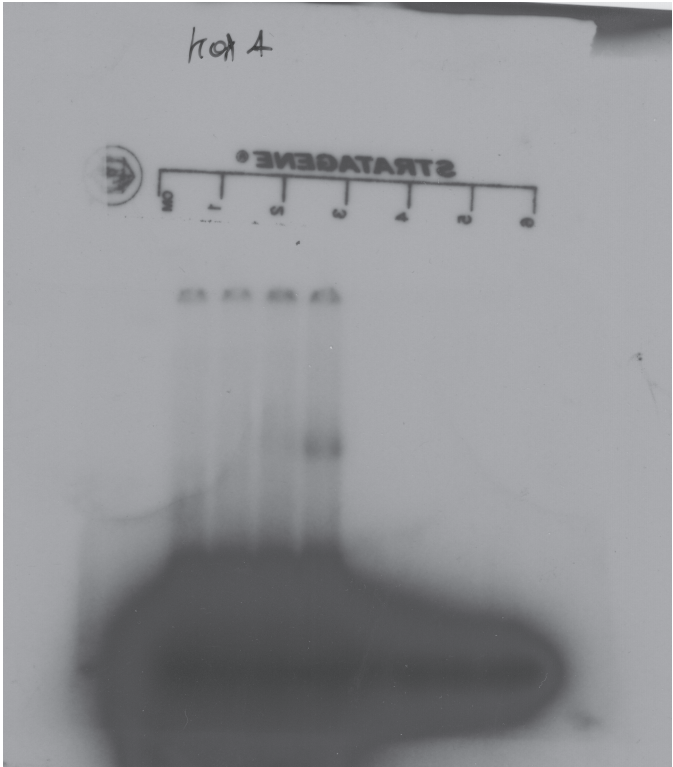

Replicate 3

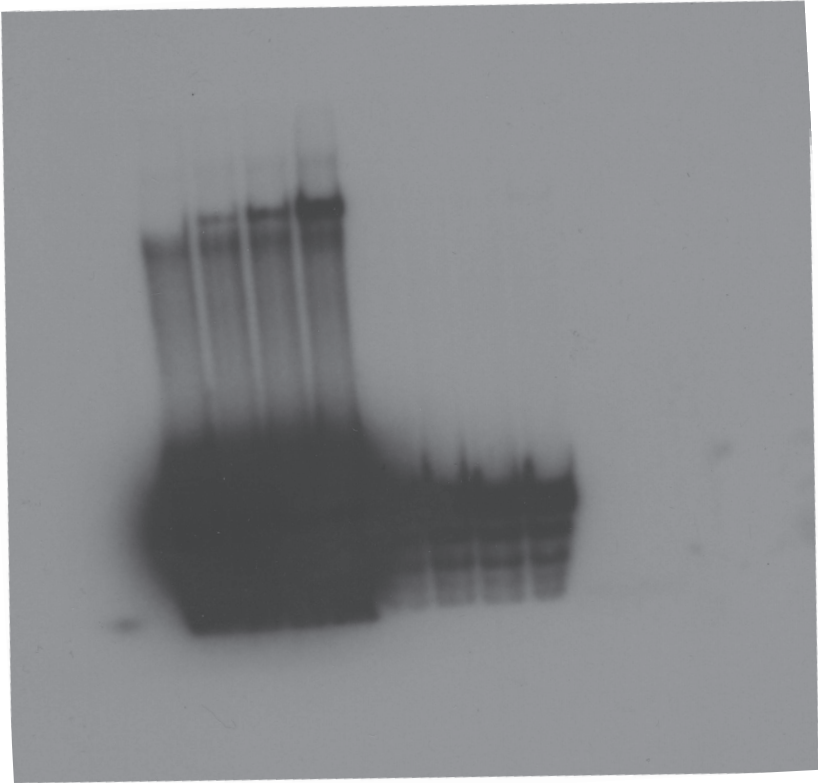

Supplement: Source Data Extended Data Fig. 6 — Uncropped EMSA blots. [file 41588_2021_1008_MOESM8_ESM.pdf]

Extended Data Figure 7

Extended Data Figure 7a

Replicate 1

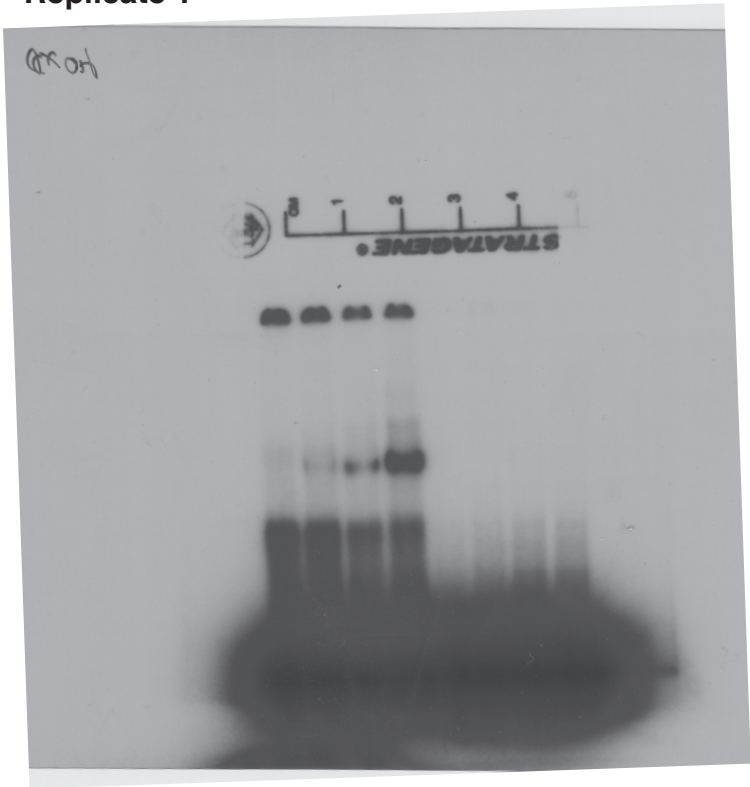

Replicate 2

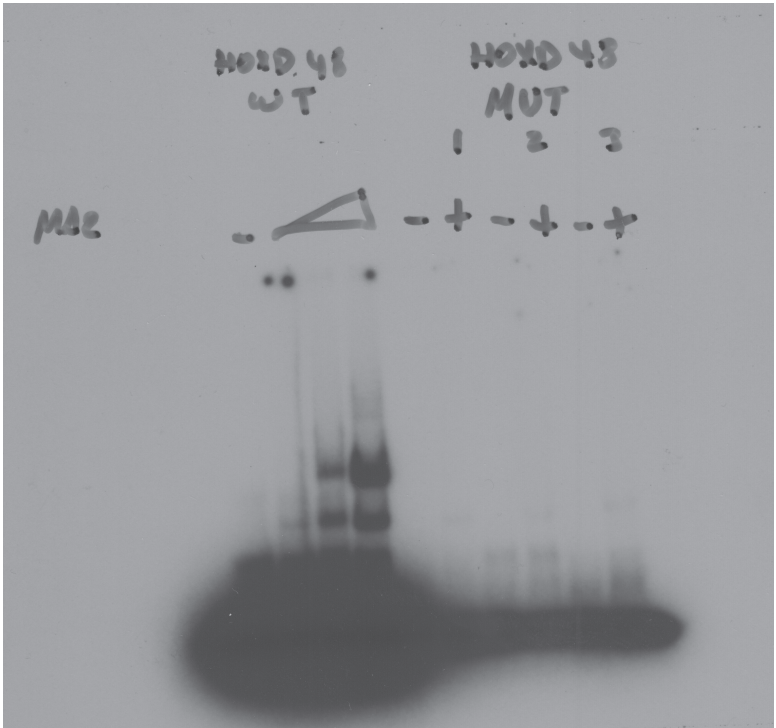

Replicate 3

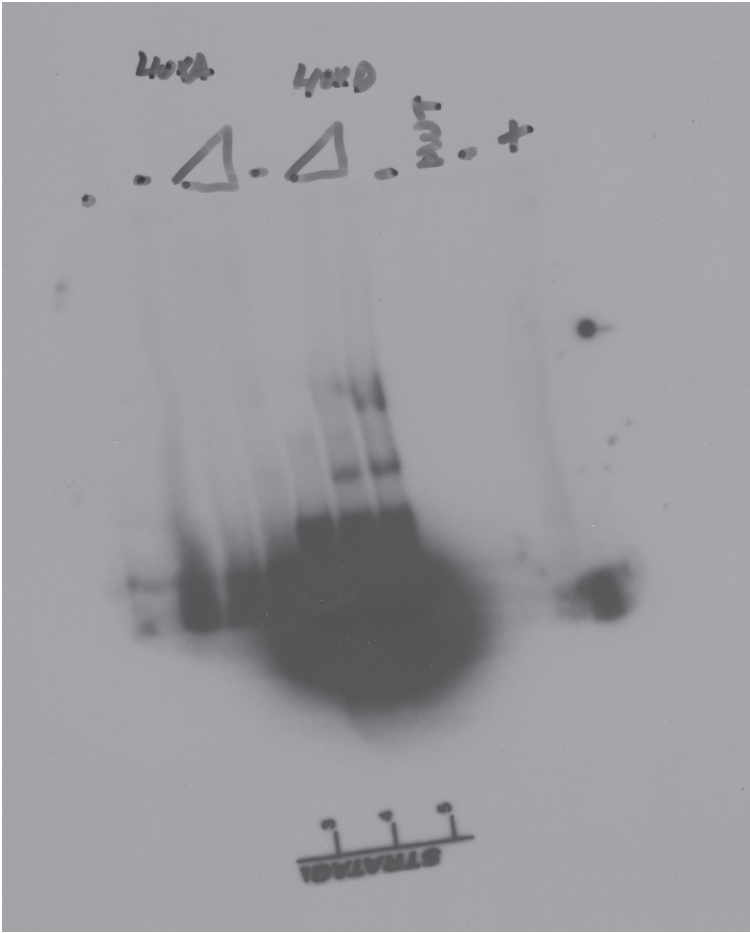

Supplement: Source Data Extended Data Fig. 7 — Uncropped EMSA blots. [file 41588_2021_1008_MOESM9_ESM.pdf]
