# Supplementary material for: CRISPR and biochemical screens identify MAZ as a cofactor in CTCF-mediated insulation at Hox clusters
Source: Nat Genet. 2022 Feb 10;54(2):202–12. doi: 10.1038/s41588-021-01008-5 (PMC8837555; doi:10.1038/s41588-021-01008-5)
Supplement: Source Data Extended Data Fig. 2 — Uncropped western blots. [file 41588_2021_1008_MOESM6_ESM.pdf]

Extended Data Figure 2

Extended Data Figure S2b

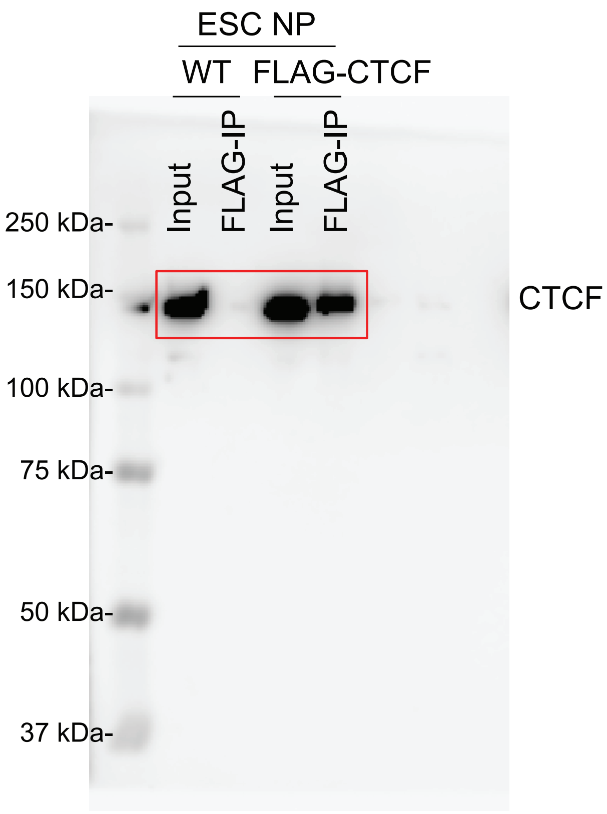

Extended Data Figure S2e

ESC blots (left)

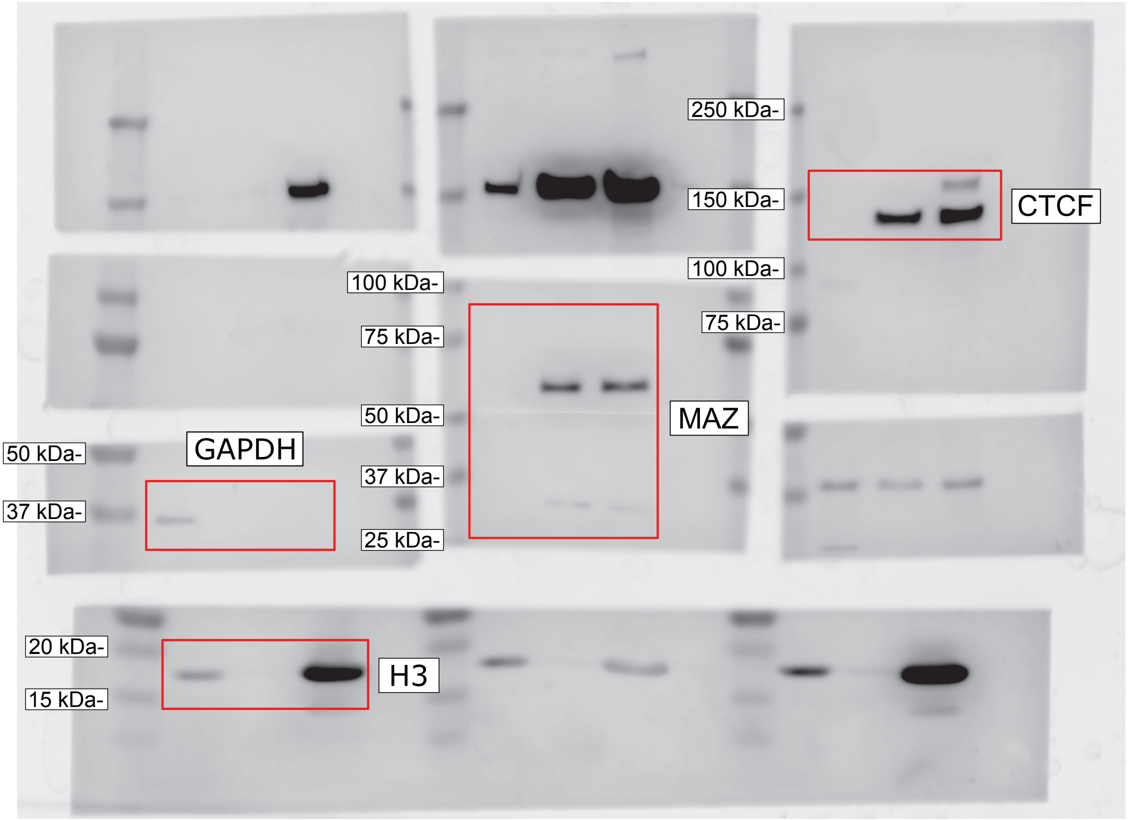

MN blots (right)

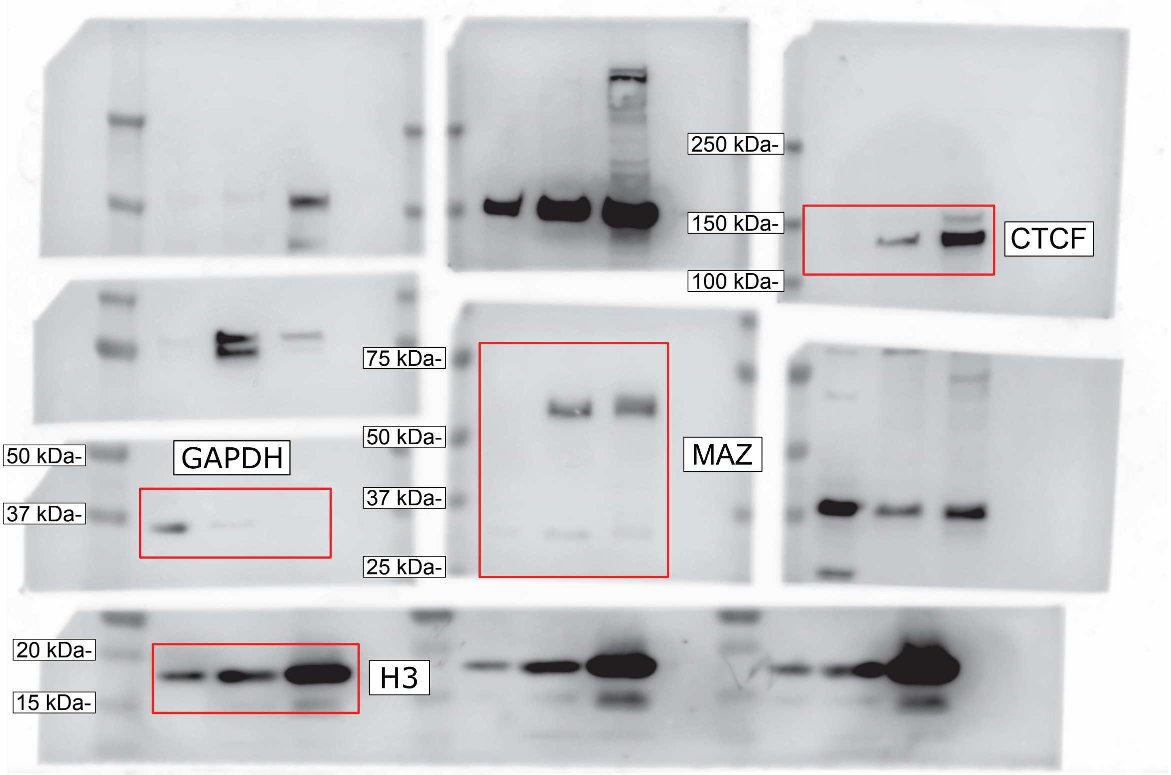

Figure S2f

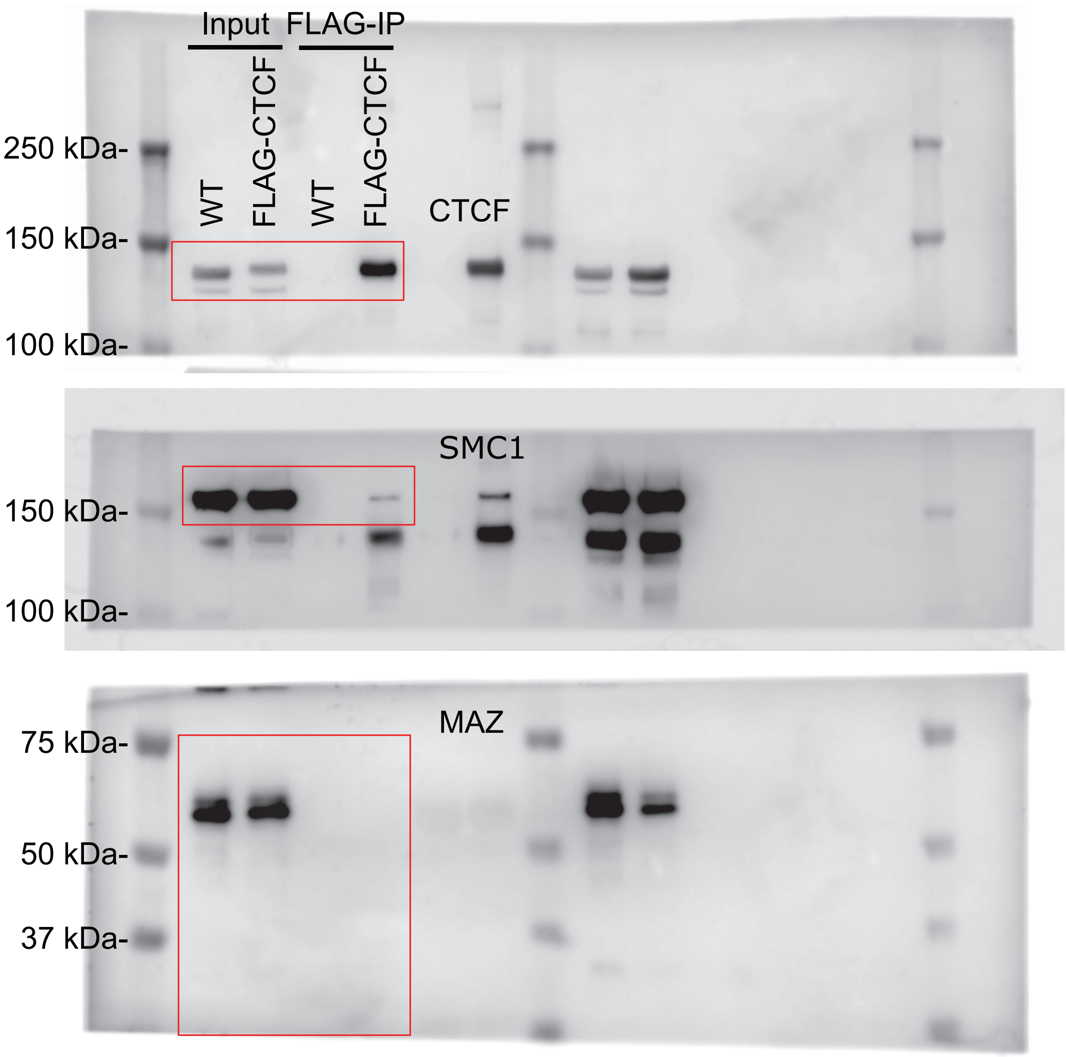

Input
FLAG-IP
WT
FLAG-CTCF
WT
FLAG-CTCF
250 kDa-
150 kDa-
100 kDa-
CTCF
SMC1
150 kDa-
100 kDa-
MAZ
75 kDa-
50 kDa-
37 kDa-

Figure S2g

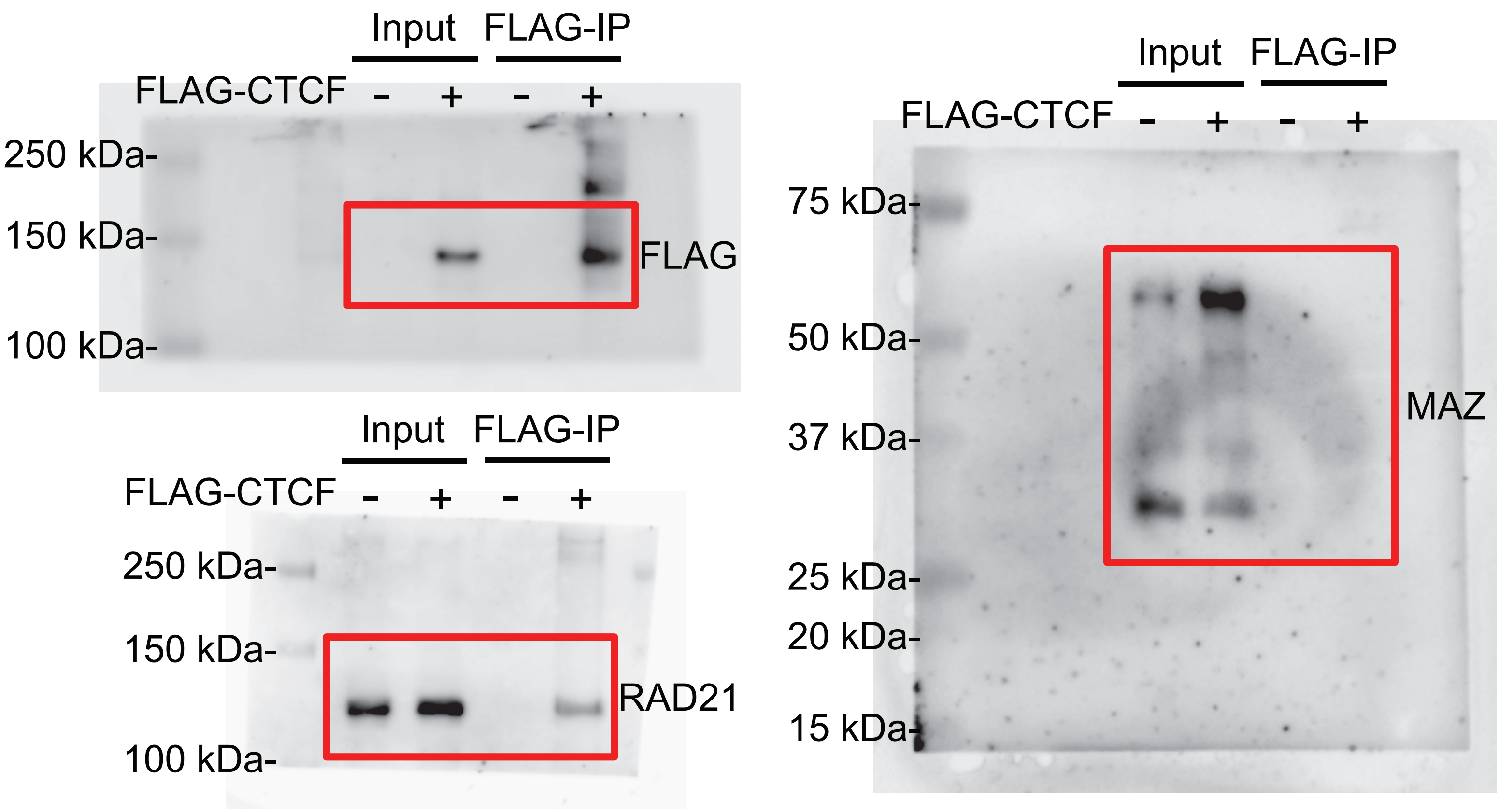
Input
FLAG-IP
FLAG-CTCF - + - +
250 kDa-
150 kDa-
100 kDa-
FLAG
Input
FLAG-IP
FLAG-CTCF - + - +
250 kDa-
150 kDa-
100 kDa-
RAD21
Input
FLAG-IP
FLAG-CTCF - + - +
75 kDa-
50 kDa-
37 kDa-
25 kDa-
20 kDa-
15 kDa-
MAZ
